# Design, synthesis, and biological profiling of fluorinated cannabidiol and cannabigerol derivatives as promising therapeutic agents

**DOI:** 10.1186/s42238-026-00403-1

**Published:** 2026-02-05

**Authors:** Ferenc Dániel Petróczi, Angéla Tótik, Miklós Bege, József Király, Erzsébet Szabó, Zsuzsanna Szabó, Nikoletta Dobos, Rasha Ghanem Kattoub, Charu Upadhyay, Eszter Ostorházi, Jan Hodek, Jan Weber, József Arany, Dorottya Ádám, Christos C. Zouboulis, Attila Oláh, István Bajza, Árpád Tósaki, Gábor Halmos, Brijesh Rathi, Pál Herczegh, Anikó Borbás, Ilona Bereczki

**Affiliations:** 1 Department of Pharmaceutical Chemistry, University of Debrecen, Egyetem tér 1, Debrecen, H-4032 Hungary; 2 Doctoral School of Pharmaceutical Sciences, University of Debrecen, Egyetem tér 1, Debrecen, H-4032 Hungary; 3 GlycOptim Kft., Egyetem tér 1, Debrecen, H-4032 Hungary; 4 Department of Biopharmacy, University of Debrecen, Rex Ferenc utca 1, Debrecen, H-4002 Hungary; 5 Department of Pharmacology, University of Debrecen, Rex Ferenc utca 1, Debrecen, H-4002 Hungary; 6 HUN-REN-DE Pharmamodul Research Group, University of Debrecen, Nagyerdei krt. 98, Debrecen, H-4032 Hungary; 7 Laboratory for Translational Chemistry and Drug Discovery, Department of Chemistry, Hansraj College, University of Delhi, Delhi, India; 8 Department of Medical Microbiology, Semmelweis University, Nagyvárad tér 4, Budapest, H-1089 Hungary; 9 Institute of Organic Chemistry and Biochemistry of the Czech Academy of Science, Prague, Prague Czech Republic; 10 Department of Physiology, Faculty of Medicine, University of Debrecen, Nagyerdei krt. 98, Debrecen, 4032 Hungary; 11 Doctoral School of Molecular Medicine, University of Debrecen, Egyetem tér 1, Debrecen, H-4032 Hungary; 12 Departments of Dermatology, Venereology, Allergology and Immunology, Staedtisches Klinikum Dessau, Brandenburg Medical School Theodor Fontane and Faculty of Health Sciences Brandenburg, Dessau, 06847 Germany; 13 National Laboratory of Virology, University of Pécs, Pécs, Ifjúság útja 20, H-7624 Hungary

**Keywords:** Cannabidiol, Cannabigerol, Mannich-type reaction, Fluorinated cannabinoids, Sebaceous lipogenesis, Malaria, Anticancer effect

## Abstract

**Background:**

Cannabidiol (CBD) and cannabigerol (CBG) are non-psychotropic phytocannabinoids that have significant, broad-spectrum therapeutic potential in a variety of pharmacological areas, but their unfavorable pharmacokinetics, such as extensive first-pass metabolism and low bioavailability, hinder their effective medical applications. Therefore, there is a great need for appropriate chemical modifications to improve their physicochemical properties. Incorporation of fluorine atom(s) at appropriate positions often improves the metabolic stability of the parent compound, increasing its bioavailability, and enhances its binding affinity to therapeutic targets, making fluorine a highly valuable element in modern drug development. Furthermore, amino functional groups may improve the water solubility and bioavailability of the compounds. Building on these principles, our strategy focused on introducing groups containing mono-, di-, and trifluoroethylamine or fluorinated aniline moieties into cannabinoids to improve their pharmacokinetic and pharmacological profiles.

**Methods:**

Mannich-type reaction was applied, using commercially available 2-fluoroethylamine, 2,2-difluoroethylamine, 2,2,2-trifluoroethylamine, 3-fluoroaniline and 4-fluoroaniline as reagents. One or two oxazine rings with fluorine-containing side chains were condensed to the aromatic core of the cannabinoids, and the formation of mono- or disubstituted derivatives was controlled by the appropriate choice of reaction conditions. The biological activity of the derivatives was investigated in various relevant fields.

**Results and conclusion:**

Our findings indicate that aliphatic modifications positively influence pharmacokinetic parameters, including absorption, in contrast to aromatic groups, which increase lipophilicity and lead to decreased bioavailability. Among the modifications, the monosubstituted derivatives containing a single oxazine ring with an aliphatic fluorine-containing side chain, especially the mono- and trifluoroethyl moieties, proved to be the most promising. These modifications appeared particularly advantageous in the CBG series compared to the properties of the CBG parent compound. This may suggest that the presence of a phenolic OH group is beneficial for biological activity. Some of the derivatives showed anticancer potential against various tumor cell lines, while others modulated sebaceous lipogenesis, and certain compounds exhibited a notable antimalarial effect.

**Supplementary Information:**

The online version contains supplementary material available at 10.1186/s42238-026-00403-1.

## Background

The prevalence of fluorine-containing medicines has seen a notable rise in recent years. This trend is clearly reflected in FDA approvals: in 2021, ten of the fifty approved drugs were fluorinated compounds (Chandra et al., 2023), while in 2023, twelve of the fifty-five approved drugs contained fluorine (Ali, et al., 2024). These figures consistently support the observation that approximately 20% of all drugs incorporate fluorine atoms. Fluorinated drug molecules are now employed across a wide array of therapeutic areas. For instance, they are used in treating cardiovascular diseases (e.g., atorvastatin and rosuvastatin), psychological disorders (fluoxetine, paroxetine), infectious diseases (ciprofloxacin and levofloxacin), diabetes (sitagliptin and gosogliptin), and tumors (5-fluorouracil and capecitabine), among many other conditions.

The replacement of H with F in a drug molecule will not change the structure and conformation drastically (Park et al. 2001). The size of a C-F unit is comparable to a C = O moiety. The dipole properties of C = O and C-F bonds are similar; therefore, they can behave similarly in dipole-dipole interactions and may act as a weak hydrogen acceptor (Park et al. 2001; Inoue et al. 2020). Fluorine atoms or a CF_3_ group can prevent metabolic transformations such as oxidation, hydroxylation, or hydrolysis increasing metabolic stability of a drug candidate (Park et al. 2001). C-F bond is more polarized than C-H bond, which alters the lipophilicity of a compound. A single hydrogen-fluorine replacement does not cause a significant change; however, a CF_3_ group can make the parent molecule more lipophilic or hydrophilic depending on the position of the substituent (Muller 1986). Certain fluorine-containing groups may enhance lipophilicity of drugs and therefore facilitate passive diffusion across membranes helping the penetration of a drug to the site of action e.g., into the brain or a tumor. Fluorine substituent in drugs can increase the strength of the effect, for example, 6α- and 9α-fluorination of glucocorticoids lead to increased glucocorticoid and mineralocorticoid activities. Moreover, fluoro-substituted steroids (e.g., dexamethasone and betamethasone) have a better skin penetration than non-fluorinated steroids (Nicolaides, 2000).

Cannabidiol (CBD) and cannabigerol (CBG) are two key non-psychotropic phytocannabinoids found in *Cannabis sativa L.* CBD has been approved for medicinal use as Epidiolex^®^ (for the treatment of two rare epilepsy disorders) (Abu-Sawwa and Stehling, 2020) and in Sativex^®^ (Nabiximols, to relieve the symptoms of multiple sclerosis) (D’hooghe et al., 2021), while CBG has not been approved for medical therapy yet. Both of them, however, show promise in a variety of therapeutic applications, and they interact with several different receptors/targets in the human body. Thus, their therapeutic potentials are currently intensively studied. They exhibit broad-spectrum activities (Lee et al. 2024; Li et al. 2024), including antioxidant, neuroprotective, antimicrobial, and anticancer properties, and possess benefits for various skin conditions, such as anti-acne treatment due to their strong anti-inflammatory and sebum-regulating properties. CBD has promising antiproliferative properties in different cancer cell lines, making it a potential candidate for cancer therapy, which is supported by some clinical trials (ClinicalTrials.gov ID. NCT06148038 2025a); ClinicalTrials.gov ID. NCT04428203 2025). While CBG is less extensively researched, it is emerging as a promising cannabinoid with similar effects; indeed, we showed that CBG and its derivatives have promising antiproliferative effects (Tósaki et al. 2024; Lőrincz et al. 2023). Furthermore, CBD may have a potential in the treatment of a range of skin conditions, such as eczema, psoriasis, acne, pruritus, hair disorders, and skin cancer (Tóth et al. 2019; Yoo and Lee 2023). Clinical trials are in progress, e.g., for the treatment of acne (ClinicalTrials.gov ID. NCT06362889 2025b), rosacea (Positive BTX) and atopic dermatitis (ClinicalTrials.gov ID. NCT06022874 2025). CBG is also investigated for atopic dermatitis, and other skin disorders, where it has demonstrated beneficial effects (Jeong et al. 2025; Yoo and Lee, 2023).

Despite their significant therapeutic potential listed above, the effective application of CBD and CBG in clinical settings is limited, mainly due to their unfavorable physicochemical properties, such as poor intestinal absorption, extensive first-pass metabolism, low bioavailability, and variable brain penetration (Lacerda et al. 2025). Considering the well-studied beneficial effects of fluorination in improving the physicochemical and biological properties of drug candidates, and the advantageous effects of introducing amino groups into the molecules mainly on water solubility based on salt formation, we planned the synthesis of new fluorine- and amino group-containing cannabinoid derivatives. Fluorinated cannabinoid compounds are rarely found in the literature, just a few fluorinated cannabidiol derivatives appear in scientific publications and patents (WO2020263888 2020). For instance, 4’-F-CBD showed more pronounced anxiolytic, antidepressant, antipsychotic and anticompulsive effect in mice than CBD (Breuer et al. 2016). A fluorinated CBD-carbamate derivative was investigated in Alzheimer’s disease as potential butyrylcholinesterase inhibitor (Jiang et al. 2021).

Given that drug molecules commonly feature either a single fluorine substituent or a trifluoromethyl group, we designed derivatives incorporating aliphatic groups with one, two, or three fluorine atoms and aromatic groups with one fluorine substituent. Previously, we successfully synthesized nitrogen containing CBD and CBG derivatives by Mannich-type reaction with various biological properties (including among others antioxidant, antiproliferative, antibacterial and antiviral activity) (Tósaki et al. 2024; Lőrincz et al. 2023; Szőke et al. 2023). As a continuation and extension of this work, we planned to prepare new derivatives via Mannich-type reaction using mono-, di-, or trifluoroethylamine, 3-fluoroaniline and 4-fluoroaniline as reagents to improve lipophilicity and metabolic stability of the compounds thereby enhancing their broad-spectrum pharmacological potential. We studied the effect of the reagent (free base or HCl salt) and solvent used on the production of mono- or disubstituted derivatives with one or two *N*-substituted oxazine rings, and optimized the reaction conditions in both directions. To validate our broad-spectrum approach and assess the improved penetration into various microbial and tumor cells, we selected biological targets that are highly relevant to the known therapeutic scope of both CBD and CBG. Therefore, we investigated the antiviral, antibacterial and antiproliferative effects of the derivatives, and also evaluated their effects on certain skin cells. Moreover, an antimalarial assay was also included, because CBD is known to have antiplasmodial activity (de Sousa et al. 2021), while such an effect of CBG is unknown. Thus, we were also interested in exploring the potential of CBG and our derivatives in this area. Our goal was to identify fluorinated candidates that possess superior potency and stability compared to the parent compounds across multiple relevant therapeutic areas. To support our design strategy and predict the in vivo efficacy of our best candidates, we also performed in silico calculations to estimate the key ADMET (absorption, distribution, metabolism, excretion, and toxicity) properties.

## Methods

### Chemical synthesis

#### General methods (Lőrincz et al. 2023)

CBD and CBG were purchased from CBDepot.eu. Amines were purchased from Merck and BLDpharm.

TLC was performed on Kieselgel 60 F_254_ (Merck) with detection by immersing into ammonium molybdate-sulfuric acid solution followed by heating. Flash column chromatography was carried out using Silica gel 60 (Merck 0.040–0.063 mm).

^1^H NMR (400 and 500 MHz), ^13^C NMR (101 and 126 MHz), ^19^F NMR (659 MHz) and 2D NMR spectra were recorded with a Bruker DRX-400, Bruker Avance II 500 and Bruker Avance Neo 700 spectrometers at 298 K. Chemical shifts are referenced to Me_4_Si (0.00 ppm for ^1^H) and to the solvent residual signals. NMR spectra and the numbering of CBD and CBG derivatives can be found in Supporting Information.

ESI-QTOF MS measurements were carried out by a maXis II UHR ESI-QTOF MS instrument (Bruker), the following parameters were applied for the electrospray ion source in positive ionization mode: capillary voltage: 3.5 kV; end plate offset: 500 V; nebulizer pressure: 0.8 bar; dry gas temperature: 200 °C and dry gas flow rate: 4.5 l/min. Constant background correction was applied for each spectrum, the background was recorded before each sample by injecting the blank sample matrix (solvent). Na-formate calibrant was injected after each sample, which enabled internal calibration during data evaluation. Mass spectra were recorded by otofControl version 4.1 (build: 3.5, Bruker) and processed by Compass DataAnalysis version 4.4 (build: 200.55.2969).

Synthetic procedures and compound characterization can be found in the Supporting Information.

### Biological studies

#### Cell culturing of human sebocytes

Human immortalized SZ95 sebocytes, originated from human facial sebaceous glands (Zouboulis et al. 1999) were provided by Prof. Christos C. Zouboulis, and were cultured in Sebomed^®^ Basal Medium (Cat. No. F8205; Sigma-Aldrich) supplemented with 10% FBS (Gibco Thermo Fisher Scientific), 1 mM CaCl_2_ (Cat. No. C7902; Sigma-Aldrich), 5 ng/ml human epidermal growth factor (Cat. No. E9644; Sigma-Aldrich), Amphotericin B (1.128 µg/ml; Gibco; Cat. No. 15290026), and MycoZap™ Plus-CL (1:500; Lonza). The medium was changed every other day, and cells were subcultured at 60–70% confluence. SZ95 sebocytes were regularly checked for *Mycoplasma* contamination by using MycoAlert™ PLUS *Mycoplasma* Detection Kit (Cat. No. LT07-710; Lonza), and every assessment yielded negative result.

#### Determination of intracellular lipids (Oláh et al. 2014; Oláh et al. 2016)

For quantitative measurement of sebaceous (neutral) lipid content, SZ95 sebocytes (20,000 cells/well) were cultured in 96-well “black well/clear bottom” plates (Cat. No. 655090; Greiner Bio-One, Frickenhausen, Germany), and were treated with compounds in the presence or absence of arachidonic acid (AA; Cat. No. 90010; Cayman Chemical Company, Ann Arbor, MI, USA) or its vehicle (absolute ethanol) as indicated. Subsequently, supernatants were discarded, cells were washed twice with phosphate-buffered saline (PBS; 115 mM NaCl, 20 mM Na_2_HPO_4_, pH 7.4; all from Sigma-Aldrich), and 100 µl of a 1 µg/ml Nile Red (Cat. No. N3013; Sigma-Aldrich) solution in PBS was added to each well. The plates were then incubated at 37 °C for 30 min, and fluorescence was measured on FlexStation 3 multimode microplate reader (Molecular Devices, San Francisco, CA, USA). Results, measured in relative fluorescence units, are expressed as percentage of the vehicle (absolute ethanol; Cat. No. 20821.296; VWR International)-treated control regarded as 100%, using 485 nm excitation and 565 nm emission wavelengths for neutral (sebaceous) lipids.

Data were analyzed by GraphPad Prism 10.4.1 (627) (GraphPad Software, LLC, San Diego, CA, USA) using two-way ANOVA followed by Dunnett’s multiple comparisons test, and *P* ≤ 0.05 values were regarded as significant differences. Graphs were plotted using GraphPad Prism 10.4.1 (627) (GraphPad Software, LLC).

#### Cell culturing of RCC cell lines

Human renal carcinoma cell lines, A-498 and CAKI-2 obtained from the American Type Culture Collection (ATCC, Rockville, MD, USA) were cultured in Iscove’s Modified Dulbecco’s Medium (IMDM) supplemented with 10% Fetal Bovine Serum (FBS) and antibiotics (100 U/ml penicillin and 100 µg/ml streptomycin) and maintained at 37 °C in a humidified atmosphere under 5% CO_2_/95% air.

#### Study of cytotoxic effects of CBD and CBG derivatives on human kidney cancer cells

CAKI-2 and A-498 human RCC cells were used for the experiments. The cytotoxic effects of newly synthesized CBD and CBG derivatives were assessed with Cell Titer Blue Assay (Promega, Madison, WI, USA) (Tósaki et al. 2024). The assay is based on the ability of living cells to convert a redox dye (resazurin) into a fluorescent product (resorufin), detailed description can be found in Tósaki et al. 2024.

Cells were seeded into 96-well (6 × 10^3^ cells/well) plates applying their complete growth media for 24 h before the treatments. To test the effect of CBG and CBD compounds on the cell proliferation activity of these two cell lines the new compounds were applied on the cells at 10, 30 µM and 60 µM over 24 to 72 h at 37 °C in the cell culture incubator with 5% CO_2_. Dimethyl sulfoxide (DMSO) was used as a vehicle, thus the control group of the cells was treated with equal volume of DMSO only. Parent compounds, CBD and CBG were applied for comparisons.

Following the treatments at each 24 h the growth media was removed from the cells and replaced with fresh media (100 µl/well). 20 µl of Cell-Titer Blue reagent from the kit (Promega, Madison, WI, USA) was added to each well of the cells and, thus, the plates were incubated for 2 h at 37 °C in the cell culture incubator, afterwards the fluorescence intensity was measured at 560/590 nm using the BioTek Plate Reader system (BioTek, Winooski, VT, USA).

#### Statistical analysis

Three independent experiments were carried out and then the data obtained from the Cell Titer Blue Assays were analyzed using the unpaired Student’s t-test using GraphPad Prism software (version 5.01; San Diego, CA). A *p* value ≤ 0.05 was set as the threshold for statistical significance.

#### Cell culturing of uveal melanoma and human breast cancer cell lines

OCM-1 and OCM-3 human primary uveal melanoma (UM) cell lines were kindly provided by the Department of Biophysics and Cell Biology, University of Debrecen. MDA-MB-231 and MCF-7 human breast cancer cell lines were obtained from the American Tissue Culture Collection (ATCC). The cells were cultured in RPMI 1640 medium supplemented with L-glutamine, 10% fetal bovine serum (FBS), and 1% penicillin/streptomycin or in DMEM medium supplemented with 10% fetal bovine serum (FBS), and 1% penicillin/streptomycin, respectively. All cells were cultured under humidified conditions (95%) in 5% CO_2_ at 37 °C. Cells were subcultured every 3 days using a standard trypsinization procedure.

#### Treatment of uveal melanoma and breast cancer cells

OCM-1, OCM-3, MDA-MB-231 and MCF-7 cells were seeded on 96 well plates (10.000 cells/well) and grown overnight. Medium was removed the following day, and replaced with assay medium containing complete growth medium RPMI-1640 (OCM-1, OCM-3) or DMEM (MDA-MB-231, MCF-7) and either 10 or 30 µM CBD- and CBG-derivatives dissolved in DMSO. Cells were treated for 24 and 48 h. Control cells were either incubated in culture medium or in DMSO with the concentration reflecting the CBD- and CBG-derivative treatments. 1% Triton X-100 treatment served as the positive control. All treatments were carried out in biological replicates (*n* = 8*–*10).

#### MTT cell viability assay.

24 and 48 h after CBD- and CBG-derivative treatments cell viability was determined by the colorimetric MTT (3-(4,5-dimethylthiazol-2-yl-)2,5-diphenyltetrazolium bromide) assay as described previously (Mosmann 1983). A total of 100 µl MTT (5 mg/ml MTT in PBS, pH 7.2) solution was added to each well of a 96 well plate and incubated at 37 °C for 2*–*4 h in dark. After incubation, 100 µl of DMSO was added to each well and mixed thoroughly to dissolve the dark blue/purple formazan crystals. The absorbance of each well was determined after 15 min of incubation with an automated ELISA plate reader (FLUOstar Optima, BMG Labtech) at 560 nm with a reference filter at 660 nm. The relative viability of the cell was expressed as a percentage of viable cells compared to untreated control cells. All experiments were carried out in biological replicates (*n* = 8*–*10).

#### Statistical analysis (MTT assay)

All experiments were conducted in biological replicates (*n* = 8*–*10). Two-way analysis of variance (ANOVA) and Dunnett’s post hoc test as implemented in GraphPad Prism 8 was used to calculate statistical significance using all experimental values. Data are expressed as mean of the biological replicates ± standard error of the mean (SEM).

#### In vitro parasite cultivation

Following the methods described by Trager and Jensen, *Pf*3D7 was grown (Trager and Jensen 1976). Briefly, a complete medium [RPMI 1640 (by Merck), 0.2% NaHCO_3_, and 0.5% AlbuMax II, supplemented with the 0.1 mM hypoxanthine and 0.5 ml of gentamycin (used stock: 50 mg/ml gentamicin] was used to develop parasites in human O^+^ RBCs. Parasites were cultivated in the presence of a gas mixture (5% O_2_, 5% CO_2_, and 90% N_2_) and maintained at 37 °C. The morphology of the parasite was monitored using the Giemsa-stained blood method (Upadhyay et al. 2022). Sorbitol treatment was performed to synchronise the culture (Read 1992).

#### Antimalarial activity in the erythrocytic stage

Parasite growth inhibition assays were performed on synchronized parasites cultured in sterile 96-well plates at a total of 100 µl volume per well, 0.5% initial parasitaemia, and 2% haematocrit. Plates were placed in an airtight chamber flushed with 5% O_2_, 5% CO_2_, and 90% N_2_ for 48 h. After 48 h, SYBR Green solution (100 µl of 0.2 µl SYBR Green per ml lysis buffer) was then added to each well and the plates were shaken in the dark at room temperature for 1 h. Then, using excitation at 490 nm and emission at 530 nm, fluorescence was captured in a SpectraMax Mini plate reader.

#### Drug-likeness properties and in-silico ADMET

The molecular properties of the monosubstituted CBD derivatives (**6M** and **8M**) and CBG derivative **13M**, along with their parent molecules, were calculated using Accelrys Discovery Studio 3.5, then filtered based on Lipinski’s Rule of Five (Lipinski et al. 1997).

The absorption, distribution, metabolism, excretion, and toxicity of the selected compounds were predicted using ADMET descriptors in Accelrys Discovery Studio 3.5. This software employs six mathematical models to quantitatively assess these properties. The models predict human intestinal absorption (HIA) following oral administration, aqueous solubility at 25 ˚C, blood-brain barrier (BBB) penetration following oral administration, cytochrome P450 2D6 (CYP2D6) enzyme inhibition, hepatotoxicity, and plasma protein binding (PPB). The models evaluating intestinal absorption and blood-brain penetration incorporate 95% and 99% confidence ellipses in the ADMET_AlogP98 vs. ADMET_PSA 2D plane. Additionally, selected compounds were evaluated for AMES mutagenicity using Accelrys Discovery Studio 3.5.

## Results and discussion

### Synthesis

**Scheme 1:**
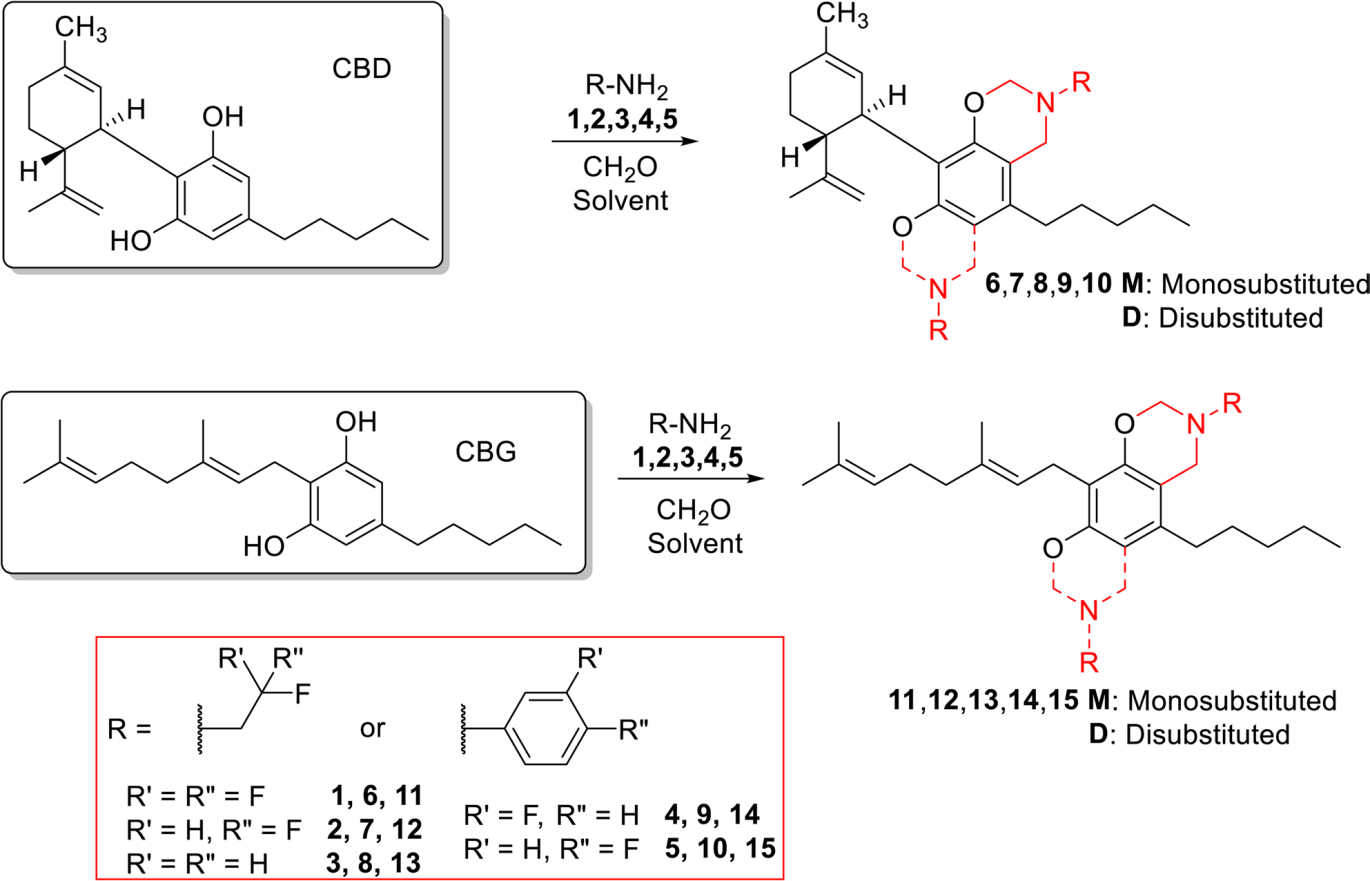
Synthesis of fluorinated CBD and CBG derivatives by Mannich-type reaction. For reaction conditions and yields, see Tables 1 and 2

The Mannich-type reaction of CBD and CBG (Scheme 1) was studied using different fluorine-containing aliphatic and aromatic amines, including 2,2,2-trifluoroethylamine (**1**), 2,2-difluoroethylamine (**2**), 2-fluoroethylamine (**3**), 3-fluoroaniline (**4**) and 4-fluoroaniline (**5**). Some of them are available as free bases, some as HCl salts, and some in both forms.

**Table 1:**
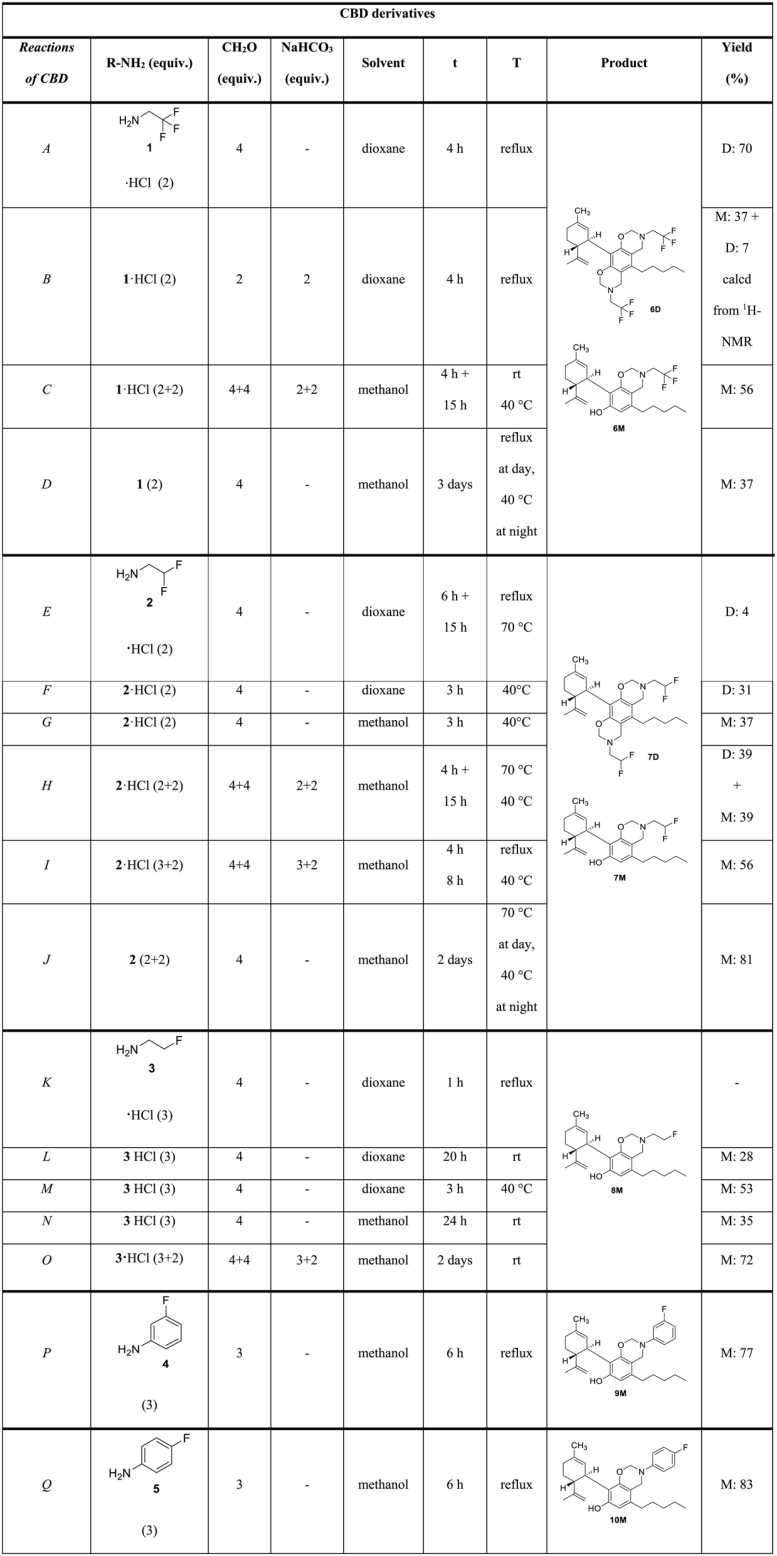
Optimalization of the reactions of CBD with fluorinated alkyl- and arylamines

*equiv. *equivalents, *t *reaction time, *T *temperature, *M *monosubstituted product, *D *disubstituted product, *rt *room temperature

**Table 2:**
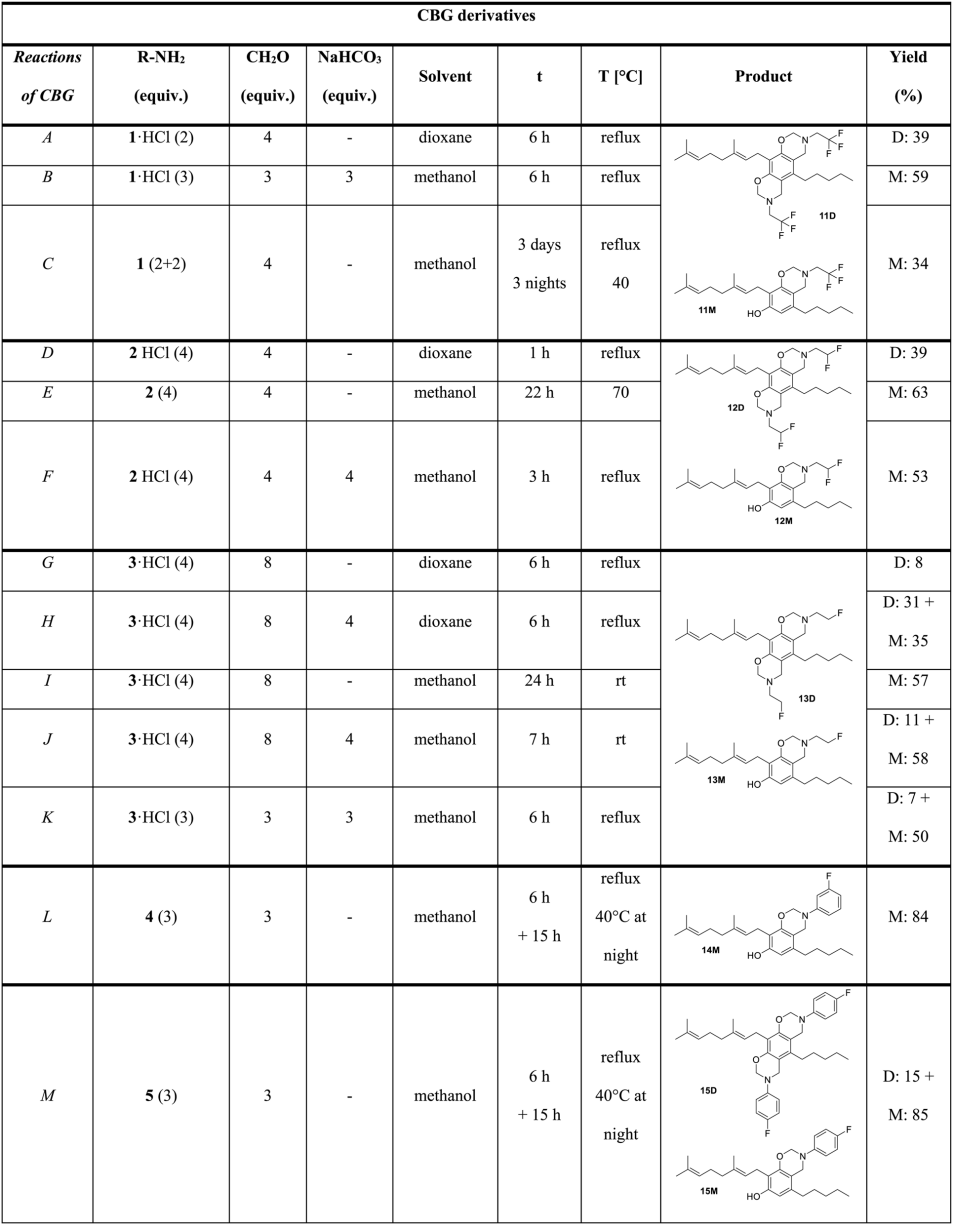
Reactions of CBG with fluorinated alkyl- and arylamines

*equiv.* equivalents, *t *reaction time, *T *temperature, *M *monosubstituted product, *D *disubstituted product, *rt *room temperature

First, the reaction of CBD with 2,2,2-trifluoroethylamine (**1**) HCl salt and formaldehyde in dioxane at reflux temperature resulted in the disubstituted product with good yield of 70% (**6D**, Table 1, Reaction *A*). It was surprising, as previously performed Mannich-type reactions with non-fluorinated amines resulted in monosubstituted CBD derivatives exclusively (Lőrincz et al. 2023). Then we attempted to improve the yield of this reaction by the addition of NaHCO_3_ to liberate the free base from 2,2,2-trifluoroethylamine·HCl. In this case, unexpectedly the monosubstituted derivative was produced with higher yield (**6M** 37%, **6D** 7%,). Since the yield was low, the solvent was changed to methanol and the monosubstituted compound was formed with a higher yield of 56% (**6M**, Reaction *C*). As the free base form is available of 2,2,2-trifluoroethylamine, it was used in the reaction with CBD, which also resulted in the monosubstituted product (**6M**) but in a lower yield of 37%.

The reaction with 2,2-difluoroethylamine (**2**) hydrochloride and formaldehyde in dioxane under the previously applied reflux conditions yielded a complex reaction mixture. At lower temperature using the HCl salt form of the reagent in dioxane resulted in the disubstituted **7D**, while in methanol, the monosubstituted derivative (**7M**) is produced (Table 1, Reactions *F* and *G*). By the liberation of the free base 2,2-difluoroethylamine (**2**) with NaHCO_3_, the monosubstituted derivative (**7M**) was produced as expected, however, at lower temperature, both the mono- and disubstituted products were observed in 1:1 ratio (Reactions *H* and *I*). Using free base reagent, predictably the monosubstituted derivative was produced with a high yield of 81% (Reaction *J*).

2-Fluoroethylamine (**3**) is only available as HCl salt. Surprisingly, under all reaction conditions tested with this reagent, both in dioxane and methanol, only the monosubstituted product was obtained (**8M**, Table 1, Reactions *K*-*O*).

Fluorinated aromatic amines 3-fluoroaniline (**4**) and 4-fluoroaniline (**5**) were used as free base forms (HCl salts are not available commercially). Since methanol seemed to be better solvent, the reactions were performed in methanol. After 6 h at reflux temperature monosubstituted Mannich-type products were formed with good yields (**9M** 77% and **10M** 83%).

Overall, it can be concluded that using the HCl salt form of the amines in dioxane eventuates disubstituted derivatives, while the use of the free base forms of the reagents and methanol as a solvent favors the formation of monosubstituted products.

The Mannich-type reactions of CBG were also performed with the previously mentioned aliphatic and aromatic amines 1,2,3,4,5 (Scheme 1, Table 2). In the reaction with 2,2,2-trifluoroethylamine (**1**) hydrochloride using dioxane as solvent the disubstituted derivative (**11D**, Table 2, reaction *A*) was formed, as it was observed in the case of CBD. In methanol, if the free base was used or it was liberated by NaHCO_3_ the expected monosubstituted product (**11M**) was formed in a predictable manner (Reactions *B* and *C*).

If CBG was reacted with 2,2-difluoroethylamine (**2**), as previously seen, the use of the HCl salt reagent in dioxane led to the formation of disubstituted product (**12D**, Table 4, Reaction *D*), while the reaction with 2-fluoroethylamine (**3**) in dioxane resulted in a complex reaction mixture with low yield of the disubstituted product (**13D**, Reaction *G*). However, the use of methanol as solvent and the presence of the free base favored the formation of the monosubstituted products in both cases (**12M**, **13M**, Reactions *E*-*F* and *H*-*K*).

In the case of 3- and 4-fluoroaniline (**4** and **5**), the reactions were carried out in methanol and, as expected, the monosubstituted products (**14M** and **15M**) were formed in high yields of 84 and 85%, respectively. In the case of 4-fluoroaniline (**5**), in addition to the monosubstituted derivative, the disubstituted (**15D**) was surprisingly also formed in 15% yield unexpectedly (Reactions *L* and *M*).

### CBD- and CBG-derivatives exert differential effects on human sebocytes

**Fig. 1:**
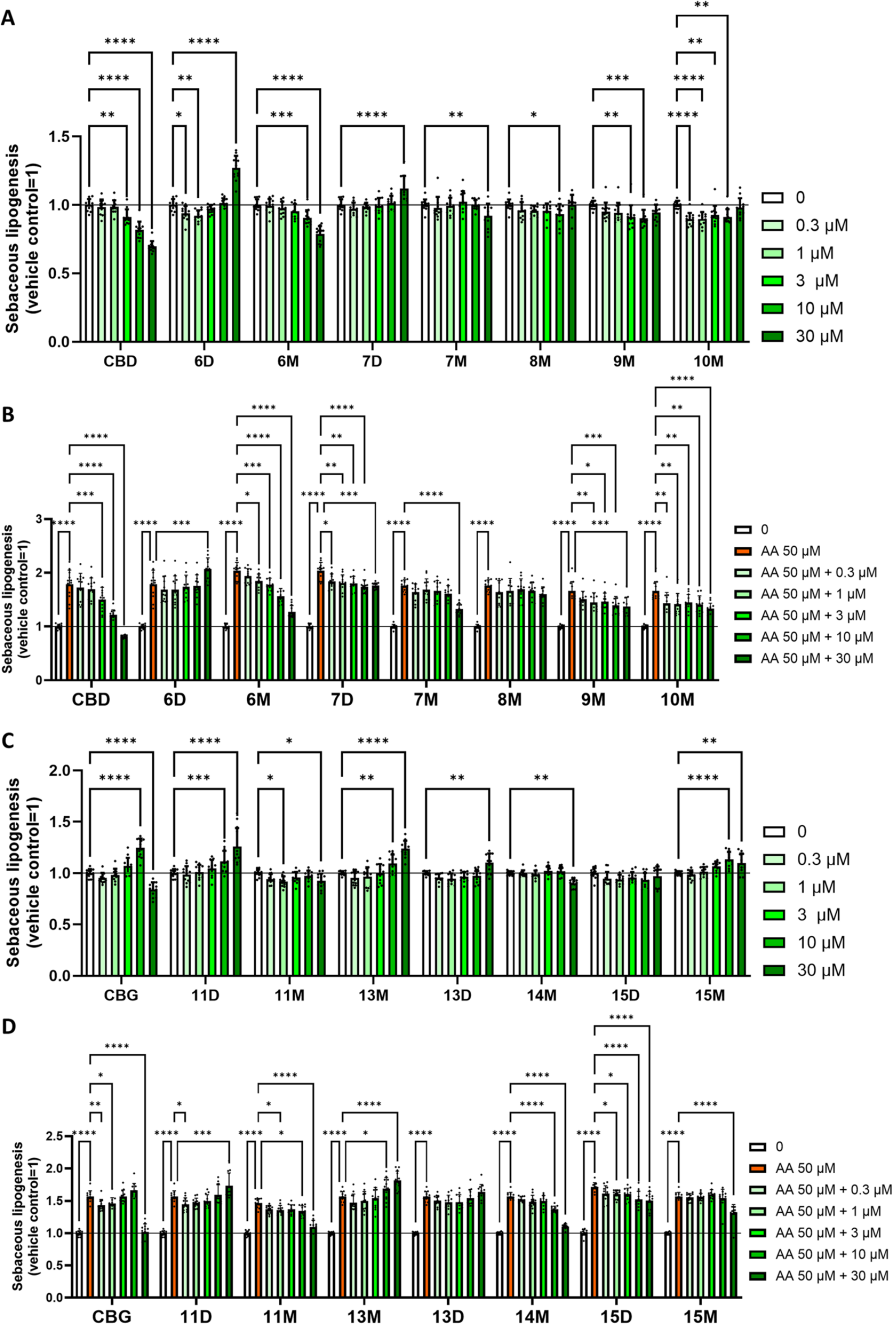
CBD- and CBG-derivatives differentially modulate sebaceous lipogenesis. Nile Red labeling for neutral (sebaceous) lipids. Lipogenesis of SZ95 sebocytes was assessed following the indicated 24 h treatments. (**A**: CBD and CBD-derivatives alone; **B**: CBD and CBD-derivatives together with AA; **C**: CBG and CBG-derivatives alone; **D**: CBG and CBG-derivatives together with AA). Mean of the vehicle-treated control group is regarded as 1. Data are presented as mean ± SD of *N* = 12 biological replicates. *, **, ***, and ****P ≤ 0.05, 0.01, 0.001, and 0.0001, respectively, as indicated. ns: not significant. AA: arachidonic acid

Phytocannabinoids have been shown to exert various effects on human sebocytes (Tóth et al. 2019). Indeed, while CBD exerted complex anti-acne activity (Oláh et al. 2014), CBG behaved in an “endocannabinoid-like” manner, and promoted sebaceous lipogenesis (Oláh et al. 2016). Hence, we assessed how the newly synthesized CBD- and CBG-derivatives influence sebaceous lipogenesis of human sebocytes. We found that CBD (≥ 3 µM) suppressed both spontaneous (Fig. 1A) as well as arachidonic acid (AA)-induced, “acne mimicking” sebaceous lipogenesis (Fig. 1B) in a concentration-dependent manner. While the actions of compound **6M** resembled to the ones of CBD, other CBD-derivatives (compounds **7M**, **8M**, **9M**, **10M**) exhibited less pronounced effects. Interestingly, instead of suppressing it, two derivatives (compounds **6D** [more efficient] and **7D** [less efficient]) were found to rather promote sebaceous lipogenesis, indicating that monosubstitution (leading to the presence of one phenolic OH group) of CBD may preserve the lipostatic actions, whereas disubstitution (leading to the lack of OH groups) results in a more “endocannabinoid-like” or “CBG-like” behavior.

While assessing the effects of the CBG-derivatives, we found that (albeit with lower potency) **11D**, **13M**, **13D**, and **15M** preserved the lipogenic effect of the mother molecule (Fig. 1C). Interestingly, **11M** and **14M** appeared to slightly, but significantly decrease sebaceous lipogenesis, whereas **15D** had no measurable effect on this experimental endpoint (Fig. 1C). Interestingly, in the case of CBG, no obvious conclusions can be drawn regarding structure-effect relationships (Fig. 1C-D).

### Antiproliferative effect against cancer cell lines

The antiproliferative potential of cannabinoids has gained significant research interest due to their abilities to inhibit or slow down the growth and division of certain tumor cells. Previously, we have synthesized several nitrogen containing derivatives with significant antiproliferative effects: two tricyclic CBG derivatives proved to be more effective against the investigated breast, ovarian and uterine cell lines than the parent compound CBG (Lőrincz et al. 2023), and one of them effectively and selectively inhibited the proliferation of human cutaneous melanoma cell lines (WM35, A2058, WM3000) (Tósaki et al. 2024). Building on the insights gained from the previous findings, we decided to investigate the effects of our synthesized compounds on various cancer cell lines.

#### Effect on the proliferation of human renal cell carcinoma cell lines

**Fig. 2:**
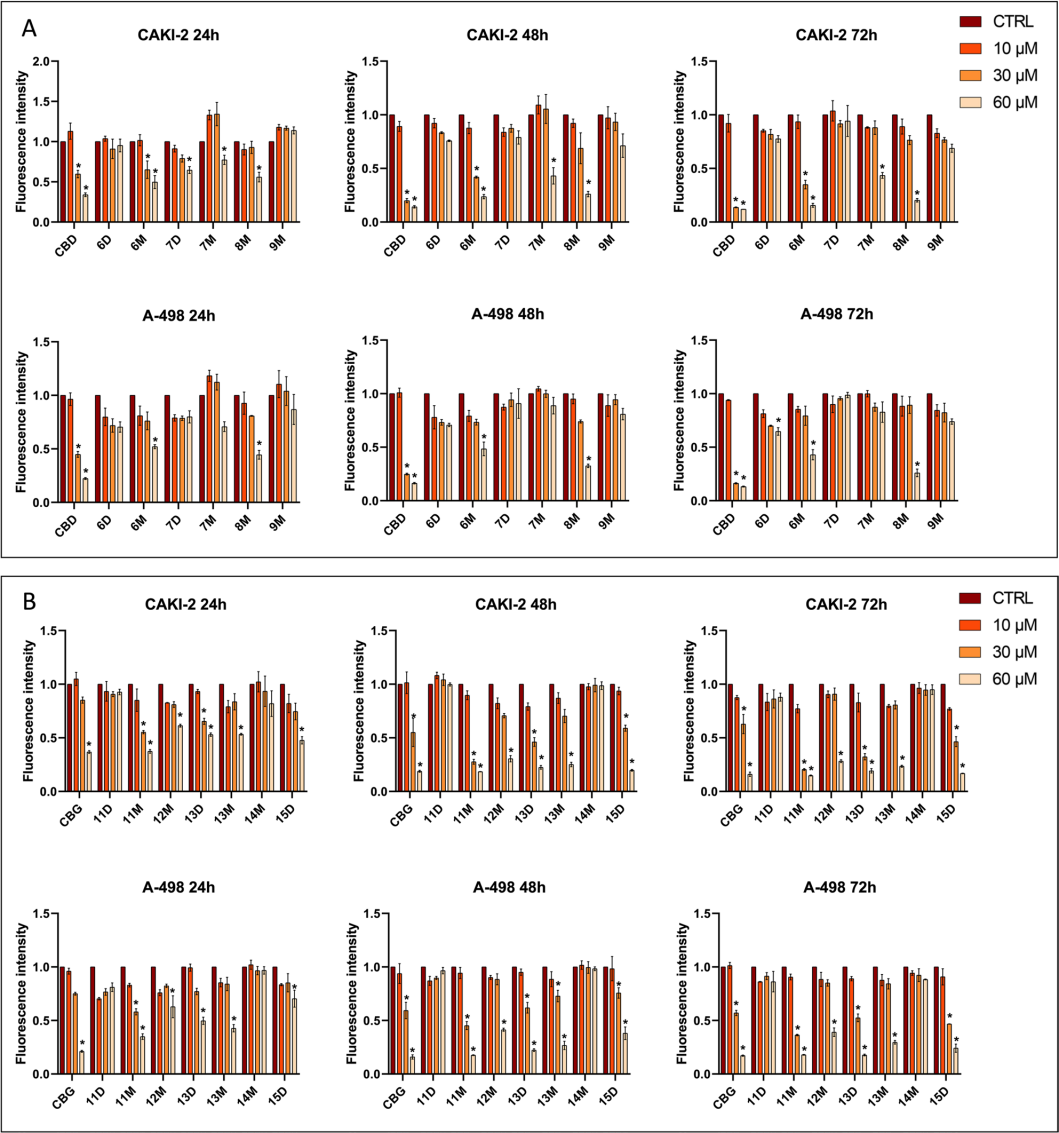
Inhibitory effects of CBD (**A**) and CBG derivatives (**B**) on human renal cancer cells. A-498 and CAKI-2 cell lines plated with 6 × 10^3^ density were treated with 10, 30 and 60 µM of the examined compounds, respectively, and fluorescence intensities of living cells were measured to determine the cytotxic effect of the compounds. The intensity of treated samples was normalized to the untreated control samples. Value of 0.05 was set as the threshold for statistical significance. The figure represents mean ± SD

Human renal cell carcinoma (RCC) cell lines, CAKI-2 and A-498, were utilized to assess the cell proliferation inhibitory effects of the newly synthesized CBD and CBG derivatives. The CBD derivatives **6D**, **6M**, **7D**, **7M**, **8M** and **9M** and CBG derivates **11D**, **11M**, **12M**, **13D**, **13M**, **14M** and **15D** were tested at 10, 30 and 60 µM concentrations and the effects on the treated cells were compared with the vehicle-treated control group of cells (DMSO treated control group, CTRL) and with the effect of the parent compounds CBD and CBG. Cell proliferation was monitored over a period of 24 to 72 h using the Cell Titer-Blue Assay (Fig. 2).

As Fig. 2A illustrates, the parent compound (CBD) significantly inhibited the proliferation of RCC cell lines, CAKI-2 and A-498, leading to a notable reduction of cell viability after 48 h of treatment at a concentration of 30 µM, compared to control groups. Similarly, the CBD derivatives **6M** and **8M** also inhibited the proliferation of both cell lines after 72 h of treatment at the concentration of 60 µM. In contrast, compound **7M** significantly inhibited the proliferation of CAKI-2 cells, but exerted no significant effect on A-498 cells, indicating a potential cell-line-specific response to this derivative (see the consolidated potency table in the Supporting Information, Table S5). While the other derivatives (**6D**, **7D** and **9M**) showed no detectable effect on either CAKI-2 or A-498 cell proliferation, suggesting that these specific modifications affected moieties presence of which are crucial for the development of the antiproliferative activity of the parent compound (CBD). The lack of activity from these derivatives could be due to structural properties that impair their ability to interact with key molecular targets involved in cell growth regulation. Compounds **6D** and **7D** are bulky tricyclic disubstituted derivatives and **9M**, although a monosubstituted derivative, contains a sterically large aromatic substituent. In contrast, the effective derivatives (**6M**, **7M**, **8M**) are all monosubstituted with one oxazine ring, in which one of the aromatic hydroxyl groups of the parent compound remains intact.

When examining cannabigerol and its derivatives, we observed that CBG demonstrated a significant antiproliferative effect on renal cell carcinoma (RCC) cell lines. CBG was found to inhibit RCC proliferation by around 60% at a concentration of 30 µM, with a markedly stronger inhibition observed at 60 µM. However, the antiproliferative activity of CBG appeared to be less potent when compared to that of cannabidiol (Fig. 2). While examining CBG derivatives, we identified that specific compounds, including **11M**, **13D**, and **15D**, exhibited a significant reduction in cell proliferation in both the CAKI-2 and A-498 RCC cell lines after 72 h of treatment at a concentration of 30 µM. These derivatives displayed a level of efficacy that seems to be greater than the parent compound (CBG), suggesting that they may hold potential as more effective alternatives in therapeutic strategies targeting renal carcinoma. Furthermore, compound **12M** and **13M** proved to be similarly active against both cell lines at a higher concentration of 60 µM. While other CBG derivatives, such as **11D**, and **14M** revealed a lack of significant antiproliferative effects on both cell lines. As it was also seen in the studies conducted on skin cells, while a structure-effect relationship can be established in the case of CBD derivatives, the antiproliferative effects of CBG derivatives do not appear to follow any consistent structural pattern. Although the monosubstituted derivatives containing mono-, di-, and trifluoroalkyl groups also demonstrated outstanding activity within the CBG series.

These findings suggest that certain CBD and CBG derivatives may serve as promising candidates for further research and development in targeted therapies for RCC, potentially offering advantages over the parent compounds, CBD and CBG.

#### Effects on the cell viability of human breast cancer cell lines

**Fig. 3:**
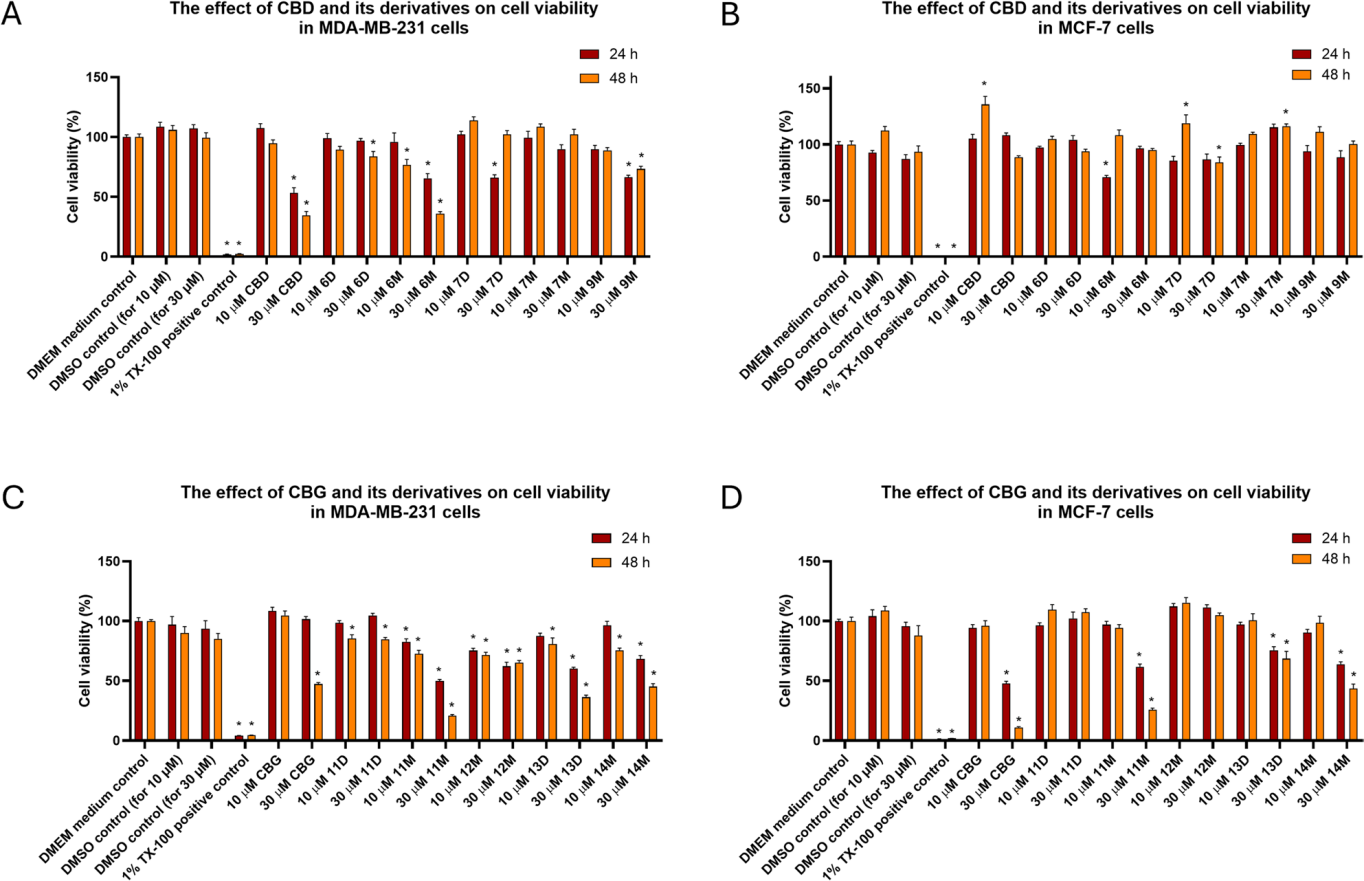
The effect of CBD, CBG and their derivatives on cell viability in breast cancer cells. MDA-MB-231 and MCF-7 cell lines were treated with CBD, CBG, or their derivatives. Cell survival was assessed following 24 and 48 h treatments using MTT assay. Values represent mean ± SEM, *P ≤ 0.05. **A** is the effect of CBD and its derivatives on cell viability in MDA-MB-231 cells; **B** is the effect of CBD and its derivatives on cell viability in MCF-7 cells; **C** is the effect of CBG and its derivatives on cell viability in MDA-MB-231 cells; **D** is the effect of CBG and its derivatives on cell viability in MCF-7 cells

We also investigated the inhibitory effect of the newly synthesized CBD (**6D**, **6M**, **7D**, **7M** and **9M**) and CBG (**11D**, **11M**, **12M**, **13D** and **14M**) derivatives on the viability of MDA-MB-231 and MCF-7 breast cancer cell lines. Their effects at 10 and 30 µM concentrations were compared to appropriate control groups (DMSO solvent, DMEM medium and 1% Triton X-100 treatment serving as a positive control), as well as to the respective parent compounds. Statistical analysis was only performed between the treatment groups and DMEM medium control. Cell survival was monitored over a period of 24 to 48 h following the exposure to the compounds using MTT assay (Fig. 3).

As shown on Fig. 3A, CBD and its derivatives (except **7D** and **7M**) significantly reduced the viability of MDA-MB-231 cells compared to DMEM medium control. Treatment with 1% Triton X-100 was used as a reliable positive control. Furthermore, a dose-dependent and time-dependent cell killing effect is discernible: the 30 µM concentration of the tested compounds consistently showed a more prominent effect in reducing cell viability compared to the 10 µM treatment. The graph illustrates a cumulative effect between the 24-hour and 48-hour treatments for some compounds. This indicates an increased loss of cell activity when the compounds were allowed to act on these cells for an extended period of time. Among the tested compounds, the parent CBD and its derivatives, **6M** and **9M** (albeit to a lesser extent), emerged as the three most effective, all at 30 µM concentrations. Both **6M** and **9M** are monosubstituted derivatives with a single oxazine ring and a free hydroxyl group.

Interestingly, the reduction in cell viability in comparison with the controls was less obvious in case of the estrogen receptor-positive (ER+) MCF-7 cells as compared to the triple-negative breast cancer cell line, MDA-MB-231 (Fig. 3B). Moreover, some compounds (e.g. CBD and **7D**) appeared to rather increase the signal intensity when applied at 10 µM (Fig. 3B). In some studies, it has been suggested that estrogen receptor-negative (ER˗) cells are more sensitive to cannabinoids than estrogen receptor-positives (ER+). This increased sensitivity of ER˗ (estrogen receptor-negative) cancers to cannabinoids might be attributed to the overexpression of CB_2_ receptors in these specific cancer types (Caffarel et al. 2012).

Next, we assessed the effects of CBG and its derivatives on the viability of the MDA-MB-231 and the MCF-7 cell lines. Here the three most prominent compounds are the parent compound CBG, and the monosubstituted **11M** and **14M** at the concentration of 30 µM. Strong similarity was observed in the percentage of cell viability decrease across both cell lines, indicating a clear concentration- and time-dependent killing effect. Both of these derivatives also demonstrated significant activity against human renal cancer cell lines.

#### Effects on the cell viability of human uveal melanoma cell lines

**Fig. 4:**
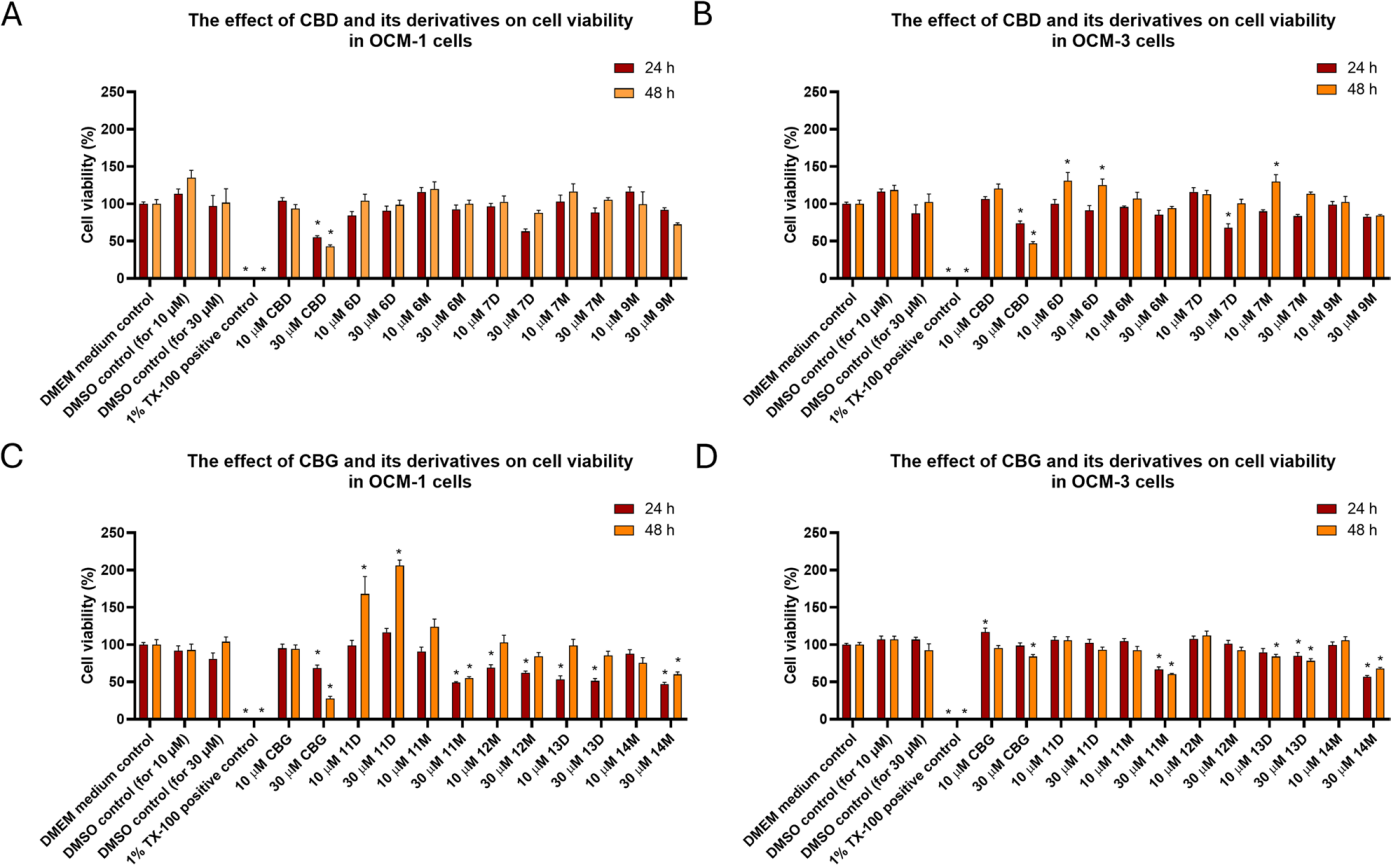
The effect of CBD, CBG and their derivatives on cell viability in uveal melanoma cells. OCM-1 and OCM-3 cell lines were treated with CBD, CBG or their derivatives. Cell survival was assessed following 24 and 48 h treatments using MTT assay. Values represent mean ± SEM, *P ≤ 0.05. **A** is the effect of CBD and its derivatives on cell viability in OCM-1cells; **B** is the effect of CBD derivatives on cell viability in OCM-3 cells; **C** is the effect of CBG and its derivatives on cell viability in OCM-1cells; **D** is the effect of CBG derivatives on cell viability in OCM-3 cells

Uveal melanoma is the most common primary intraocular malignancy in adults. OCM-1 and OCM-3 cell lines were used for the investigations. The cells were treated with the compounds at concentrations of 10 and 30 µM and their effects on cell survival were then monitored over a 24 to 48-hour period using MTT assay (Fig. 4). Effects of the derivatives were compared to appropriate control (DMSO [vehicle] or DMEM [culture medium]; positive control: 1% Triton X-100), as well as to the respective parent compounds.

CBD effectively inhibited cell viability in OCM-1 and OCM-3 cells at 30 µM concentration for 24 and 48 h. A significant, though not overwhelming, effect was also found at 30 µM treatment of **7D** after 24 h. While compounds **6D** and **7M** slightly, but increased cell proliferation of OCM-3 cells (Fig. 4A and B).

CBG effectively inhibited cell viability in OCM-1, but, interestingly not of OCM-3 cells at 30 µM concentration for 24 and 48 h (Fig. 4C and D). In OCM-1 cells, a significant, though not overwhelming, effect was also found following 30 µM treatment of **11M** and **14M** after 24 and 48 h. Interestingly, compound **11D** increased cell proliferation of OCM-1 cells at 10 and 30 µM after 48 h. In OCM-3 cell line a significant effect was also found after 30 µM treatment of **11M**, **13D** (also 10 µM after 48 h was effective) and **14M** after 24 and 48 h. Interestingly, 10 µM CBG slightly increased the signal intensity, i.e., it might have increased the cell count after 24 h (Fig. 4C and D).

Considering all the antiproliferative results, it can be stated that the monosubstituted CBD and CBG derivatives containing a single oxazine ring and a trifluoroethyl side chain proved to be the most effective modifications (see the details in the consolidated potency table in Supporting Information, Table S5). The data also suggest that the modifications of CBG proved to be more advantageous and effective compared to the corresponding compounds synthesized from CBD. Although sometimes negative and sometimes positive effects on cell proliferation observed with synthetic cannabinoid derivatives may seem contradictory, these results confirm the literature findings that present a very mixed picture regarding the antiproliferative effects of cannabinoids. While many studies show beneficial results, demonstrating that (at certain concentrations) cannabinoids can inhibit cancer cell growth, other studies found no significant effects, and in some instances, even tumor-promoting effects have been observed (Cridge and Rosengren 2013). These varied outcomes underscore the complex nature of cannabinoid interactions, which depend predominantly on factors such as the specific cannabinoid type, its dosage, the cancer type being studied, and the experimental model system used. For example, while cannabinoids show promising effects in inhibiting the growth of certain breast cancer cells, other studies also indicate that they can increase cell viability in specific scenarios, especially at lower concentrations or when interacting with particular cell types (Almeida et al. 2021; Malfitano et al. 2011; Fonseca et al. 2017).

Therefore, further research is necessary to fully understand the role of natural and semi-synthetic cannabinoids in the regulation of cell viability in different contexts. Moreover, special attention should be paid to investigate their effects on the anti-tumor immune response as well as on their putative interactions with the metabolism of conventional chemotherapeutic agents (Oláh et al. 2017).

### Antiplasmodial activity

#### In vitro screening of novel compounds against *PfC580Y* strain

The emergence of artemisinin resistance in *Plasmodium falciparum (Pf)* particularly associated with the C580Y mutation in the Kelch13 gene, necessitates the discovery of new chemotherapeutic agents (Azmi et al. 2023). Intriguingly, the antimalarial activity of cannabinoids has recently garnered significant attention, particularly concerning THC (tetrahydrocannabinol), though CBD has also been noted (de Sousa et al. 2021). Cannabis is traditionally used against malaria, which is mentioned in the world’s oldest pharmacopoeia from about 5000 years ago. THC, the main psychotropic phytocannabinoid in *Cannabis sativa*, was proven to inhibit β-haematin formation (synthetic haemozoin) and malaria parasite growth. In this study, CBD was also tested, because it lacks the psychotropic effects of THC, and it inhibited β-haematin formation, but showed only a mild antimalarial activity (de Sousa et al. 2021). This inspired us to evaluate a set of nine CBD and CBG derivatives for their antiplasmodial activity against the *PfC580Y* strain. Each compound was tested at three concentrations (in triplicate): 100 µM, 10 µM, and 1 µM, with a positive control DHA (dihydroartemisinin) at 10 nM (74.8% inhibition) and CQ (chloroquine) at 1 µM (100% inhibition). The % inhibition of parasite growth was assessed using standardized in vitro assays, and the values are mentioned in Table 3.

**Table 3:** In vitro percentage Inhibition of synthesized compounds against PfC580Y strain

Compounds	% Inhibition (C580Y)		
	100 µM	10 µM	1 µM
CBD	73.1 ± 4.0	58.1 ± 0.8	**43.3** ± 4.0
**6D**	59.6 ± 7.5	47.5 ± 0.9	**44.2** ± 3.9
**6M**	82.4 ± 7.2	61.9 ± 3.3	34.1 ± 5.1
**8M**	67.7 ± 0.2	54.5 ± 2.9	**48.8** ± 4.1
**10M**	76.0 ± 4.2	54.4 ± 5.0	**49.0** ± 3.4
CBG	76.8 ± 2.4	52.7 ± 3.2	8.5 ± 2.9
**11D**	42.9 ± 5.0	39.8 ± 1.3	33.8 ± 3.0
**11M**	78.2 ± 1.9	57.5 ± 3.3	26.6 ± 4.1
**13M**	71.1 ± 3.0	55.7 ± 3.9	36.7 ± 4.6
**15D**	49.1 ± 7.0	22.4 ± 5.7	7.9 ± 0.8
**15M**	57.9 ± 7.7	8.2 ± 3.4	12.9 ± 2.0

Compounds derived from CBD exhibited stronger activity at 1 µM against *PfC580Y strain*. Notably, **8M** and **10M** showed the highest inhibitory effects (48.8% and 49.0%, respectively), surpassing the parent CBD itself (43.3%). Similarly, **6D** also retained activity (44.2%). These results suggest that structural modifications of CBD enhance low-dose efficacy. In contrast, CBG and its analogues showed markedly lower efficacy at 1 µM. The parent CBG exhibited only 8.5% inhibition, and most derivatives, including **15D** and **15M**, displayed < 15% activity. Only **13M** (36.7%) showed moderate inhibition, but still lower than the best-performing CBD derivatives.

The comparative analysis highlights that CBD and its derivatives are significantly more potent against *PfC580Y* strain at 1 µM than CBG-based compounds, suggesting that the CBD scaffold provides a superior chemical framework for low-dose antiplasmodial (*PfC580Y* strain*)* activity. This could be attributed to favorable pharmacophoric features of the CBD core, which may enhance binding interactions with parasite biomolecular targets compared to the CBG scaffold. Thus, CBD-derived analogues, particularly **8M** and **10M**, represent promising leads for further optimization in antimalarial drug discovery.

A heatmap is generated based on the percentage inhibition values to visualize and prioritize compounds for downstream evaluations (Figure S1 in Supporting Information).

### Evaluation of the antibacterial and antiviral activity

Our previous research has demonstrated the antiviral and antibacterial effects of both CBD and CBG (Lőrincz et al. 2023). Their Mannich-type products showed lower antibacterial activity, however, some of the derivatives proved to be highly effective against SARS-CoV-2. Therefore, as a part of this study, we investigated the fluorine containing derivatives against a panel of bacteria (*Bacillus subtilis*, methicillin-susceptible *Staphylococcus aureus*, methicillin-resistant *Staphylococcus aureus*, *Staphylococcus epidermidis* biofilm, *Enterococcus faecalis* 29 212, 15 376 VanA and 51299 VanB) and SARS-CoV-2 as well, but the derivatives have not shown a significant efficacy against these pathogens. Some of the derivatives demonstrated moderate activity against *Enterococcus faecalis* strains, with minimal inhibitory concentrations of 32–128 µg/ml (Table S4), while other derivatives showed activity against SARS-CoV-2 with low micromolar IC_50_ values, however, they also proved to be cytotoxic within the same low concentration range (Table S3). For further details, please see the Supporting Information file.

### Drug-likeness properties and in Silico ADMET analysis

Drug-likeness and in silico ADMET (absorption, distribution, metabolism, excretion, and toxicity) analysis are very useful in modern drug discovery. They help researchers quickly find the best potential drug candidates, speeding up development and increasing the chances of creating safe and effective new medicines. The oral bioavailability of a drug is influenced by its solubility and ability to cross the intestinal wall, both of which are related to its physicochemical properties. The molecular properties of the most promising monosubstituted CBD derivatives (**6M** with a trifluoromethyl group and **8M** with one fluorine) and the CBG derivative **13M** with one fluorine, along with their parent compounds, were calculated and then filtered based on Lipinski’s Rule of Five (Table S3 in Supporting Information), which predicts that poor absorption or permeation is more likely if a compound is characterized by two or more of the following properties: more than 5 hydrogen bond donors, more than 10 hydrogen bond acceptors, a molecular weight greater than 500 g/mol, or a calculated LogP (CLogP) greater than 5 (or MlogP > 4.15) (Lipinski et al. 1997). All compounds met Lipinski’s rule with one violation (LogP > 5). The Log*P* values were calculated for all of the compounds. The results demonstrate that the derivatives, containing aliphatic side chains have lower lipophilicity, than the parent CBD and CBG, suggesting enhanced absorption and ultimately, bioavailability; whereas those with a fluor-containing aromatic moiety exhibit the highest Log*P* values (see the consolidated potency table in the Supporting Information, Table S5).

**Fig. 5:**
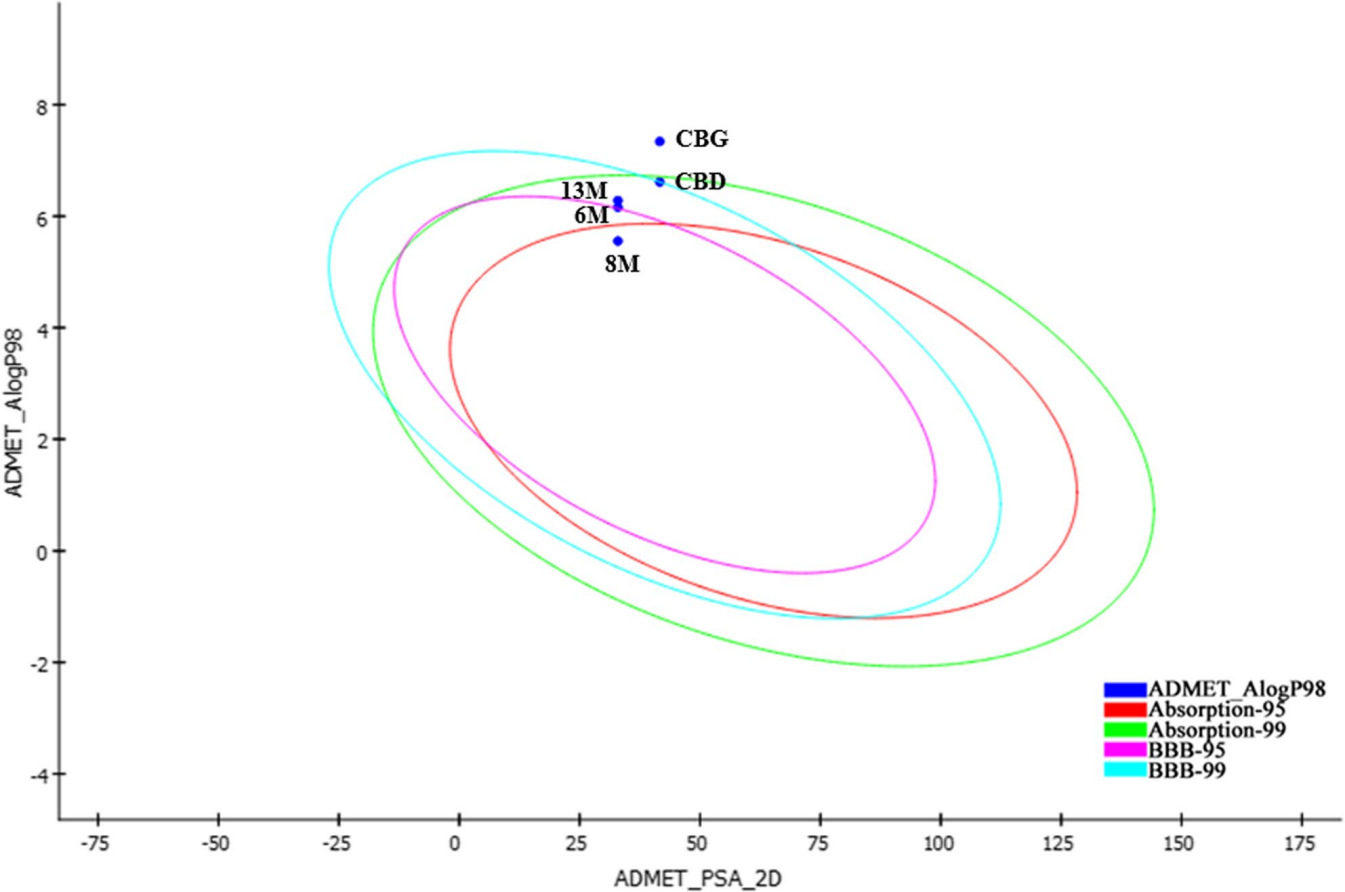
Calculated ADMET_ALogP98 vs. ADMET_PSA 2D. The figure shows 95% and 99% confidence ellipses corresponding to human intestinal absorption (HIA) and blood-brain barrier (BBB) penetration models for CBD, CBG, **6M**, **8M**, and **13M**

Polar surface area (PSA) is a well-known molecular descriptor used to evaluate cellular permeability, including intestinal absorption and blood-brain barrier (BBB) penetration. Compounds with a PSA of 140 Å^2^ or lower are generally expected to have good oral bioavailability. PSA values together with AlogP can be utilized to accurately predict the intestinal permeability of drug candidates (Egan and Lauri, 2002). In this context, an AlogP98 and PSA_2D model was developed, featuring a binary plot with 95% and 99% confidence ellipses. As demonstrated in Fig. 5, all three newly synthesized candidates fall within the 99% confidence interval of human intestinal absorption (HIA), thereby supporting the model predictions.

ADMET predictions showed that most of the tested compounds maintained similar solubility levels as their parent compounds while exhibiting enhanced absorption (**8M** compared to CBD, and notably, **13M** compared to CBG (Table S4 in Supporting Information). This may result from the balance achieved through the inclusion of an amine group, which promotes salt formation, along with the presence of a fluorinated alkyl side chain. Absorption levels were predicted for all of the derivatives, and those with fluorine-containing aromatic groups showed markedly poor absorption (see the consolidated potency table in Supporting Information, Table S5).

The cytochrome P450 2D6 (CYP2D6) enzyme is involved in the metabolism of 20–25% of drugs used in clinical settings, and its activity can be significantly influenced by enzyme inhibition during drug-drug interactions (DDIs) (Rüdesheim et al. 2025). The results predict that the derivatives do not inhibit the CYP2D6 enzyme, whereas the parent compounds do, thereby indicating a more favorable profile. Moreover, similar to CBD and CBG, our newly synthesized compounds are expected to be non-hepatotoxic and non-mutagenic (Table S4 in Supporting Information).

## Conclusions

In consequence of our synthetic efforts, we successfully prepared novel fluorinated cannabidiol and cannabigerol derivatives. This was achieved under optimized conditions via Mannich-type reaction, utilizing mono-, di-, or trifluoroethylamine, along with 3- and 4-fluoroaniline reagents. We successfully controlled the outcome of the reactions. By carefully selecting the solvent and whether to use the reagent in its salt or free base form, we were able to influence the degree of substitution. Specifically, using the HCl salt form of the reagent in dioxane favored the formation of disubstituted derivatives with two oxazine rings. In contrast, using methanol as solvent and the free base form of the reagent promoted the creation of monosubstituted derivatives with one oxazine ring. Thereafter, our goal was to comprehensively map the biological activity of these derivatives and their parent compounds across relevant biological areas. We systematically evaluated their antiviral, antibacterial, antiproliferative, and antimalarial effects, as well as their impact on certain skin cells.

Furthermore, we performed computational calculations to corroborate our hypothesis that the fluorine modification and the introduction of an amino group would enhance the compounds’ pharmacokinetic profile and overall bioavailability. The results showed that derivatives containing aliphatic side chains exhibited lower lipophilicity compared to the parent CBD and CBG compounds, which correlated with better absorption. Conversely, those derivatives containing fluorine-containing aromatic groups displayed markedly higher lipophilicity and consequently, poor absorption. For some selected derivatives, we predicted the ADMET properties, providing a comprehensive assessment of their pharmacokinetic and pharmacodynamic suitability. All of these compounds met Lipinski’s rule with one violation (LogP > 5). That means the derivatives exhibit generally suitable physicochemical properties for oral bioavailability. Polar surface area calculations also indicated enhanced intestinal absorption and BBB penetration. Moreover, the selected derivatives showed no inhibition of CYP2D6, whereas the parent compounds did. This suggests that the derivatives presumably have a significantly reduced risk of drug-drug interactions and an improved safety profile.

Concerning the biological data, CBD effectively suppressed sebaceous lipogenesis, a key action for acne treatment. Certain monosubstituted CBD derivatives mimicked this action, while disubstituted ones sometimes promoted it, highlighting the role of the degree of substitution and the number of phenolic hydroxyl groups. Among the CBD derivatives, the **6M** had the most CBD-like effect. CBG derivatives showed less clear structure-activity relationships for lipogenesis, but some of them preserved the parent’s effect while others slightly decreased it.

In the case of renal cell carcinoma cell lines, our findings revealed a clear structure-activity relationship among CBD derivatives with the monosubstituted compounds **6M**, **7M** and **8M** showing high potency, while the antiproliferative effects of CBG derivatives did not exhibit a consistent pattern. For human breast cancer and uveal melanoma cell lines, the most potent cannabinoids were the parent compounds (CBD and CBG), alongside their monosubstituted derivatives (**6M**, **9M**, **11M**, and **14M**). These particularly active compounds all share a common monosubstituted, bicyclic structural motif with a free phenolic hydroxyl group, highlighting their potential as key candidates for future antitumor therapeutic development.

Although these findings are very promising, the proliferation-inducing effects observed in certain cases together with other potential challenges (e.g., impact on the anti-tumor immune response, or possible interaction with the metabolism of conventional chemotherapeutic agents), highlight the need for further investigations before considering the possibility of the clinical application of such compounds.

In antimalarial studies, CBD derivatives demonstrated comparable or even superior potency compared to the parent CBD against the *PfC580Y* strain, even at low concentrations. Moreover, while the parent CBG proved to be ineffective against malaria, the non-aromatic CBG derivatives showed remarkable antiplasmodial activity.

The findings indicate that the aliphatic modifications are preferred over the aromatic ones in terms of both bioavailability and biological activity, and the chemical modifications proved more successful in improving the biological activities of CBG derivatives compared to CBD compounds. Monosubstituted CBD and CBG derivatives with one oxazine ring bearing a monofluorinated or a trifluorinated aliphatic side chain generally prove more effective or similarly potent than their parent compounds across various therapeutic areas with lower lipophilicity and enhanced absorption level. This is particularly significant given the low water solubility, absorption, and bioavailability of native CBD and CBG. The presence of an amine group in our synthesized derivatives allows for salt formation, a well-established method to significantly boost water solubility and, consequently, bioavailability. This improvement could lead to enhanced therapeutic efficacy and allow for more effective, lower-dose administration in potential clinical applications. These significant physicochemical and ADMET advantages ˗ especially the improved absorption and metabolic stability ˗ are expected to translate into substantially greater benefits in vivo than those observed in the in vitro assays performed in this study. This differential advantage is critical to the success of any drug candidate, as it directly impacts effective dosing and systemic exposure.

Therefore, these fluorinated cannabinoid derivatives, especially the highly active monosubstituted compounds with a mono- or trifluoroethyl side chain and a phenolic OH group, represent promising candidates for the development of new and more bioavailable therapeutic agents for conditions including certain tumors, malaria, and skin disorders.

## Supplementary Information

Supplementary Material 1.

## Data Availability

The datasets used and/or analysed during the current study are included in the published article and its supplementary information file or available from the corresponding author on reasonable request.

## Abbreviations

A2058: Human cutaneous melanoma cell line
A-498: Human renal cell carcinoma cell line
AA: Arachidonic acid
ADMET: Absorption, distribution, metabolism, excretion, and toxicity
ATCC: American Tissue Culture Collection
BBB: Blood-brain barrier
CAKI-2: Human renal cell carcinoma cell line
CB_2_: Cannabinoid type 2 receptor
CBD: Cannabidiol
CBG: Cannabigerol
CTRL: Control group
DDI: Drug-drug interaction
DHA: Dihydroartemisinin
DMEM: Dulbecco′s Modified Eagle′s Medium
DMSO: Dimethyl sulfoxide
ELISA: Enzyme-Linked Immunosorbent Assay
equiv.: Equivalent
ER˗: Estrogen receptor-negative
ER+: Estrogen receptor-positive
ESI-QTOF MS: Electrospray ionization quadrupole time-of-flight mass spectrometry
FBS: Fetal Bovine Serum
FDA: U.S. Food and Drug Administration
h: Hour
HIA: Human intestinal absorption
IMDM: Iscove’s Modified Dulbecco’s Medium
LogP: Logarithm of the partition coefficient (P)
MCF-7: Michigan Cancer Foundation-7, breast cancer cell line
MDA-MB-231: MD Anderson-Metastatic Breast-231 Cells, breast cancer cell line
MTT: (3-(4, 5-dimethylthiazolyl-2)-2, 5-diphenyltetrazolium bromide)
NMR: Nuclear Magnetic Resonance
ns: not significant
O^+^ RBCs: O positive red blood cells
OCM-1: Ocular Choroidal Melanoma cell line
OCM-3: Ocular Choroidal Melanoma cell line
PBS: Phosphate-buffered saline
Pf: Plasmodium falciparum
PSA: Polar surface area
RCC: Human renal cell carcinoma
R_f_: Retention factor
RPMI 1640: Medium developed at Roswell Park Memorial Institute
SARS-CoV-2: Severe acute respiratory syndrome coronavirus 2
SD: Standard Deviation
SEM: Standard Error of the Mean
SZ95: Immortalized Human Sebaceous Gland Cell Line
T: Temperature
t: Time
THC: Tetrahydrocannabinol
TLC: Thin-Layer Chromatography
UM: Uveal melanoma
WM3000: Human cutaneous melanoma cell line
WM35: Human cutaneous melanoma cell line
