# Supplementary material for: Design, synthesis, and biological profiling of fluorinated cannabidiol and cannabigerol derivatives as promising therapeutic agents
Source: J Cannabis Res. 2026 Feb 5;8:34. doi: 10.1186/s42238-026-00403-1 (PMC12964675; doi:10.1186/s42238-026-00403-1)

**Supporting information**

**Design, Synthesis, and Biological Profiling of Fluorinated Cannabidiol and Cannabigerol Derivatives as Promising Therapeutic Agents**

Ferenc Dániel Petróczi, Angéla Tótik, Miklós Bege, József Király, Erzsébet Szabó, Zsuzsanna Szabó, Nikoletta Dobos, Rasha Ghanem Kattoub, Charu Upadhyay, Eszter Ostorházi, Jan Hodek, Jan Weber, József Arany, Dorottya Ádám, Christos C. Zouboulis, Attila Oláh, István Bajza, Árpád Tósaki, Gábor Halmos, Brijesh Rathi, Pál Herczegh, Anikó Borbás, Ilona Bereczki

Table of content

[Chemical Synthesis 2](#_Toc216424825)

[Antimalarial results 22](#_Toc216424826)

[Antiviral tests 23](#_Toc216424827)

[Antibacterial assay 25](#_Toc216424828)

[Drug-likeness properties and *in silico* ADMET analysis 26](#_Toc216424829)

[**Table S5.** Consolidated potency table. 27](#_Toc216424830)

[NMR spectra of the CBD and CBG derivatives 28](#_Toc216424831)

# Chemical Synthesis

***General methods*** *(Lőrincz et al. 2023)*

CBD and CBG were purchased from CBDepot.eu. Amines were purchased from Merck and BLDpharm.

TLC was performed on Kieselgel 60 F254 (Merck) with detection by immersing into ammonium molybdate-sulfuric acid solution followed by heating. Flash column chromatography was carried out using Silica gel 60 (Merck 0.040-0.063 mm).

^1^H NMR (400 and 500 MHz), ^13^C NMR (101 and 126 MHz), ^19^F NMR (659 MHz) and 2D NMR spectra were recorded with a Bruker DRX-400, Bruker Avance II 500 and Bruker Avance Neo 700 spectrometers at 298 K. Chemical shifts are referenced to Me_4_Si (0.00 ppm for 1H) and to the solvent residual signals.

ESI-QTOF MS measurements were carried out by a maXis II UHR ESI-QTOF MS instrument (Bruker), the following parameters were applied for the electrospray ion source in positive ionization mode: capillary voltage: 3.5 kV; end plate offset: 500 V; nebulizer pressure: 0.8 bar; dry gas temperature: 200 °C and dry gas flow rate: 4.5 l/min. Constant background correction was applied for each spectrum, the background was recorded before each sample by injecting the blank sample matrix (solvent). Na-formate calibrant was injected after each sample, which enabled internal calibration during data evaluation. Mass spectra were recorded by otofControl version 4.1 (build: 3.5, Bruker) and processed by Compass DataAnalysis version 4.4 (build: 200.55.2969).

Ref: Lőrincz EB, Tóth G, Spolárics J, Herczeg M, Hodek J, Zupkó I, Minorics R, Ádám D, Oláh A, Zouboulis CC, Weber J, Nagy L, Ostorházi E, Bácskay I, Borbás A, Herczegh P, Bereczki I. Mannich-type modifications of (-)-cannabidiol and (-)-cannabigerol leading to new, bioactive derivatives. *Sci Rep.* 2023;13(1):19618. DOI: [10.1038/s41598-023-45565-7](https://doi.org/10.1038/s41598-023-45565-7)

***Chemical synthesis***

*General reaction pathway I*

Fluorine containing reagent in hydrochloride salt form and formaldehyde (36% in water) were dissolved in the specified solvent. The mixture was stirred for 1 hour, and then cannabidiol or cannabigerol was added. The mixture was stirred at a specified temperature for a certain period of time, then the solvent was evaporated and the residue was purified by flash column chromatography.

*General reaction pathway II*

Fluorine containing reagent in hydrochloride salt form and NaHCO_3_ were dissolved in the specified solvent. The mixture was stirred for 30 minutes, and then formaldehyde (36% in water) was added. It was stirred for 30 minutes, then cannabidiol (157 mg, 0.5 mmol) was added. The mixture was stirred at a specified temperature for a certain period of time, then the solvent was evaporated and the residue was purified by flash column chromatography.

*General reaction pathway III*

Fluorine containing reagent (base form) and formaldehyde (36% in water) were dissolved in the specified solvent. The mixture was stirred for 1 hour, and then cannabidiol or cannabigerol was added. The mixture was stirred at a specified temperature for a certain period of time, then the solvent was evaporated and the residue was purified by flash column chromatography.

***Synthesis of CBD derivatives***

**Compounds 6D and 6M**

Reaction route *A*

The reaction was carried out via *reaction pathway I*.

Reagents: 2,2,2-trifluoroethylamine hydrochloride (135 mg, 1 mmol), formaldehyde (167 μl, 2 mmol); solvent: dioxane (20 ml); cannabidiol (157 mg, 0.5 mmol); reaction time and temperature: 4 hours at reflux temperature; column chromatography: hexane/acetone 98:2; yielded **6D** (197 mg, 70%) as a yellowish syrup.

Reaction route *B*

The reaction was carried out via *reaction pathway II*.

Reagents: 2,2,2-trifluoroethylamine hydrochloride (135 mg, 1 mmol), NaHCO_3_ (84 mg, 1 mmol), formaldehyde (84 μl, 1 mmol); solvent: dioxane (10 ml); cannabidiol (157 mg, 0.5 mmol); reaction time and temperature: 4 hours at reflux temperature; column chromatography: hexane/ethyl acetate 98:2; yielded **6M** (162 mg, 37%) and **6D** (38 mg, 7%) as yellowish syrups.

Reaction route *C*

The reaction was carried out via *reaction pathway II*.

Reagents: 2,2,2-trifluoroethylamine hydrochloride (2x135 mg, 2 mmol), NaHCO_3_ (2x84 mg, 2 mmol), formaldehyde (2x167 μl, 4 mmol); solvent: methanol (5 ml); cannabidiol (157 mg, 0.5 mmol); reaction time and temperature: 4 hours at room temperature and 15 hours at 40 °C; column chromatography: hexane/dichloromethane 85:15; yielded **6M** (123 mg, 56%) as yellowish syrup.

Reaction route *D*

The reaction was carried out via *reaction pathway III*.

Reagents: 2,2,2-trifluoroethylamine (78 μl, 1 mmol), formaldehyde (167 μl, 2 mmol); solvent: methanol (20 ml); cannabidiol (157 mg, 0.5 mmol); reaction time and temperature: 3 days at reflux temperature during the days and 40 °C at nights; column chromatography: hexane/acetone 98:2; yielded **6M** (80 mg, 37%) as a yellowish syrup.

**6D:** R_f_ = 0.71 (hexane/acetone 9:1); ^1^H NMR (400 MHz, CDCl_3_) δ 5.20 (s, 1H, H-2 C*H*), 4.81 – 4.64 (m, 4H, H-B C*H*_2_), 4.51 – 4.39 (m, 2H, H-9 C*H*_2_), 4.01 (s, 4H, H-A C*H*_2_), 3.96 – 3.87 (m, 1H, H-3 C*H*), 3.33 – 3.10 (m, 4H, H-1’” C*H*_2_), 2.86 (td, 1H, *J* = 11.2, 3.5 Hz, H-4 C*H*), 2.28 – 2.09 (m, 3H, H-1” C*H*_2_ and H-6 C*H*_2_a), 2.05 – 1.93 (m, 1H, H-6 C*H*_2_b), 1.82 – 1.71 (m, 2H, H-5 C*H*_2_), 1.65 (s, 3H, H-7 C*H*_3_), 1.62 (s, 3H, H-10 C*H*_3_), 1.39 – 1.30 (m, 6H, H-2” C*H*_2_, H-3” C*H*_2_ and H-4” C*H*_2_), 0.91 (t, 3H, *J* = 6.8 Hz, H-5” C*H*_3_); ^13^C NMR (101 MHz, CDCl_3_) δ 149.6, 136.5, 131.7, 129.7, 127.0 (5C, quat.), 125.3 (1C, C-2 *C*H), 124.2, 117.8 (2C, quat.), 109.9 (1C, C-9 *C*H_2_), 82.7 (2C, C-B *C*H_2_), 53.18 (q, 2C, *J* = 31.7 Hz, C-1”’ *C*H_2_-CF_3_), 50.4 (2C, C-A *C*H_2_), 45.3 (1C, C-4 *C*H), 35.8 (1C, C-3 *C*H), 32.3 (1C, C-3” *C*H_2_), 30.8 (1C, C-6 *C*H_2_), 29.7 (1C, C-5 *C*H_2_), 29.2 (1C, C-2” *C*H_2_), 27.5 (1C, C-1” *C*H_2_), 23.5 (1C, C-7 *C*H_3_), 22.6 (1C. C-4” *C*H_2_), 19.1 (1C, C-10 *C*H_3_), 14.1 (1C, C-5” *C*H_3_); ^19^F NMR (659 MHz, CDCl_3_) δ -71.43 (t, *J* = 9.1 Hz); ESI-QTOF MS: *m/z* calcd for C_29_H_38_F_6_N_2_O_2_Na^+^: 583.2730 [M+Na]^+^; found: 583.2716 (mass error: 2.4 ppm).

**6M:** R_f_ = 0.62 (hexane/acetone 9:1); ^1^H NMR (500 MHz, CDCl_3_) δ 6.30 (s, 1H, aromatic C*H*), 5.98 (s, 1H, O*H*), 5.59 (s, 1H, H-2 C*H*), 4.75 (d, *J* = 10.1 Hz, 1H, H-B C*H*_2_a), 4.67 (d, *J* = 10.0 Hz, 1H, H-B C*H*_2_b), 4.50 (s, 1H, H-9 C*H*_2_a), 4.32 (s, 1H, H-9 C*H*_2_b), 4.04 (d, *J* = 16.6 Hz, 1H, H-A C*H*_2_a), 3.97 – 3.89 (m, 2H, H-A C*H*_2_b and H-3 C*H*), 3.30 – 3.09 (m, 2H, H-1’” C*H*_2_), 2.45 – 2.30 (m, 3H, H-4 C*H* and H-1” C*H*_2_), 2.30 – 2.19 (m, 1H, H-6 C*H*_2_a), 2.14 – 2.04 (m, 1H, H-6 C*H*_2_b), 1.85 – 1.73 (m, 5H, H-5 C*H*_2_ and H-7 C*H*_3_), 1.68 (s, 3H, H-10 C*H*_3_), 1.57 – 1.44 (m, 2H, H-2” C*H*_2_), 1.40 – 1.27 (m, 4H, H-3” C*H*_2_ and H-4” C*H*_2_), 0.90 (t, 3H, *J* = 7.0 Hz, H-5” C*H*_3_); ^13^C NMR (126 MHz, CDCl_3_) δ 154.6, 152.3, 147.5, 140.0, 126.7, 124.5 (6C, quat.), 124.3 (1C, C-2 *C*H), 114.9 (1C, quat.), 111.2 (1C, C-9 *C*H_2_), 110.0 (1C, aromatic *C*H), 108.5 (1C, quat.), 82.8 (1C, C-B *C*H_2_), 53.28 (q, 1C, *J* = 31.7 Hz, C-1”’ *C*H_2_-CF_3_), 50.7 (1C, C-A *C*H_2_), 47.0 (1C, C-4 *C*H), 35.4 (1C, C-3 *C*H), 31.8 (2C, C-3” *C*H_2_ and C-1” *C*H_2_), 30.6 (1C, C-6 *C*H_2_), 29.8 (1C, C-2” *C*H_2_), 28.2 (1C, C-5 *C*H_2_), 23.8 (1C, C-7 *C*H_3_), 22.7 (1C. C-4” *C*H_2_), 18.8 (1C, C-10 *C*H_3_), 14.1 (1C, C-5” *C*H_3_); ^19^F NMR (659 MHz, CDCl_3_) δ -71.47 (t, *J* = 9.4 Hz); ESI-QTOF MS: m/z calcd for C_25_H_34_F_3_NO_2_Na^+^: 460.2434 [M+Na]^+^; found: 460.2433 (mass error: 0.2 ppm).

**Compounds 7D and 7M**

Reaction route *E*

The reaction was carried out via *reaction pathway I*.

Reagents: 2,2-difluoroethylamine hydrochloride (118 mg, 1 mmol), formaldehyde (167 μl, 2 mmol); solvent: dioxane (10 ml); cannabidiol (157 mg, 0.5 mmol); reaction time and temperature: 6 hours at reflux temperature and 15 hours at 70 °C; column chromatography: hexane/ethyl acetate 95:5; yielded **7D** (11 mg, 4%) as a yellowish syrup.

Reaction route *F*

The reaction was carried out via *reaction pathway I*.

Reagents: 2,2-difluoroethylamine hydrochloride (235 mg, 2 mmol), formaldehyde (334 μl, 4 mmol); solvent: dioxane (30 ml); cannabidiol (314 mg, 1.0 mmol); reaction time and temperature: 3 hours at 40 °C; column chromatography: hexane/ethyl acetate 95:5; yielded **7D** (162 mg, 31%) as a yellowish syrup.

Reaction route *G*

The reaction was carried out via *reaction pathway I*.

Reagents: 2,2-difluoroethylamine hydrochloride (235 mg, 2 mmol), formaldehyde (334 μl, 4 mmol); solvent: methanol (30 ml); cannabidiol (314 mg, 1 mmol); reaction time and temperature: 3 hours at 40 °C; column chromatography: hexane/ethyl acetate 97:3; yielded **7M** (157 mg, 37%) as a yellowish syrup.

Reaction route *H*

The reaction was carried out via *reaction pathway II*.

Reagents: 2,2-difluoroethylamine hydrochloride (2x118 mg, 2 mmol), NaHCO_3_ (2x84 mg, 2 mmol), formaldehyde (2x167 μl, 4 mmol); solvent: methanol (25 ml); cannabidiol (157 mg, 0.5 mmol); reaction time and temperature: 4 hours at 70 °C and 15 hours at 40 °C; column chromatography: hexane/ethyl acetate 97:3; yielded **7M** (82 mg, 39%) **7D** (103 mg, 39%) as yellowish syrups.

Reaction route *I*

The reaction was carried out via *reaction pathway II*.

Reagents: 2,2-difluoroethylamine hydrochloride (176+118 mg, 2.5 mmol), NaHCO_3_ (126+84 mg, 2.5 mmol), formaldehyde (2x167 μl, 4 mmol); solvent: methanol (10 ml); cannabidiol (157 mg, 0.5 mmol); reaction time and temperature: 4 hours at reflux temperature and 8 hours at 40 °C; column chromatography: hexane/ethyl acetate 95:5; yielded **7M** (118 mg, 56%) as yellowish syrup.

Reaction route *J*

The reaction was carried out via *reaction pathway III*.

Reagents: 2,2-difluoroethylamine (2x70 µl, 2 mmol), formaldehyde (167 μl, 2 mmol); solvent: methanol (10 ml); cannabidiol (157 mg, 0.5 mmol); reaction time and temperature: 2 days at 70 °C during the days and 40 °C at nights; column chromatography: hexane/ethyl acetate 97:3; yielded **7M** (169 mg, 81%) as yellowish syrup.

**7D:** R_f_ = 0.37 (hexane/acetone 9:1); ^1^H NMR (500 MHz, CDCl_3_) δ 5.92 (tt, 2H, *J* = 56.0, 4.2 Hz, H-2”’ C*H*), 5.21 (s, 1H, H-2 C*H*), 4.80 – 4.66 (m, 4H, H-B C*H*_2_), 4.46 (d, 2H, *J* = 14.5 Hz, H-9 C*H*_2_), 4.05 – 3.88 (m, 5H, H-A C*H*_2_ and H-3 C*H*), 3.12 – 2.96 (m, 4H, H-1’” C*H*_2_), 2.88 (td, 1H, *J* = 11.1, 3.4 Hz, H-4 C*H*), 2.25 – 2.10 (m, 3H, H-1” C*H*_2_ and H-6 C*H*_2_a), 2.04 – 1.94 (m, 1H, H-6 C*H*_2_b), 1.80 – 1.67 (m, 2H, H-5 C*H*_2_), 1.66 (s, 3H, H-7 C*H*_3_), 1.62 (s, 6H, H-10 C*H*_3_), 1.35 (s, 6H, H-2” C*H*_2_, H-3” C*H*_2_ and H-4” C*H*_2_), 0.95 – 0.87 (m, 3H, H-5” C*H*_3_); ^13^C NMR (126 MHz, CDCl_3_) δ 149.6, 136.3, 131.5 (3C, quat.), 125.5 (1C, C-2 *C*H), 116.33 (t, 2C, *J* = 241.0 Hz, *C*HF_2_), 117.5 (1C, quat.), 110.3 (1C, quat.), 109.9 (1C, C-9 *C*H_2_), 82.7 (2C, C-B *C*H_2_), 54.13 (t, 2C, *J* = 25.1 Hz, C-1”’ *C*H_2_-CHF_2_), 49.9 (2C, C-A *C*H_2_), 45.2 (1C, C-4 *C*H), 35.8 (1C, C-3 *C*H), 32.4 (1C, C-3” *C*H_2_), 30.9 (1C, C-6 *C*H_2_), 29.8 (1C, C-5 *C*H_2_), 29.1 (1C, C-2” *C*H_2_), 27.6 (1C, C-1” *C*H_2_), 23.5 (1C, C-7 *C*H_3_), 22.6 (1C. C-4” *C*H_2_), 19.1 (1C, C-10 *C*H_3_), 14.1 (1C, C-5” *C*H_3_); ^19^F NMR (659 MHz, CDCl_3_) δ -120.12 (t, *J* = 15.3 Hz), -120.21 (t, *J* = 15.3 Hz); ESI-QTOF MS: *m/z* calcd for C_29_H_40_F_4_N_2_O_2_Na^+^: 547.2918 [M+Na]^+^; found: 547.2917 (mass error: 0.2 ppm).

**7M:** R_f_ = 0.50 (hexane/acetone 9:1); ^1^H NMR (500 MHz, CDCl_3_) δ 6.29 (s, 1H, aromatic C*H*), 6.05 – 5.79 (m, 2H, H-2”’ C*H* and O*H*), 5.59 (s, 1H, H-2 C*H*), 4.74 (d, 1H, *J* = 9.9 Hz, H-B C*H*_2_a), 4.65 (d, 1H, *J* = 10.0 Hz, H-B C*H*_2_b), 4.50 (s, 1H, s, H-9 C*H*_2_a), 4.33 (s, 1H, H-9 C*H*_2_b), 4.00 (d, 1H, *J* = 16.5 Hz, H-A C*H*_2_a), 3.96 – 3.90 (m, 1H, H-3 C*H*), 3.87 (d, 1H, *J* = 16.5 Hz, H-A C*H*_2_b), 3.12 – 2.91 (m, 2H, H-1’” C*H*_2_), 2.43 – 2.30 (m, 3H, H-4 C*H* and H-1” C*H*_2_), 2.29 – 2.18 (m, 1H, H-6 C*H*_2_a), 2.13 – 2.05 (m, 1H, H-6 C*H*_2_b), 1.83 – 1.76 (m, 5H, H-5 C*H*_2_ and H-7 C*H*_3_), 1.68 (s, 3H, H-10 C*H*_3_), 1.55 – 1.47 (m, 2H, H-2” C*H*_2_), 1.37 – 1.29 (m, 4H, H-3” C*H*_2_ and H-4” C*H*_2_), 0.90 (t, 3H, *J* = 7.0 Hz, H-5” C*H*_3_); ^13^C NMR (126 MHz, CDCl_3_) δ 154.4, 152.3, 147.4, 139.9 (4C, quat.), 124.4 (1C, C-2 *C*H), 116.28 (t, 1C, *J* = 240.8 Hz, *C*HF_2_), 114.7 (1C, quat.), 111.3 (1C, C-9 *C*H_2_), 109.8 (1C, aromatic *C*H), 108.7 (1C, quat.), 82.8 (1C, C-B *C*H_2_), 54.16 (t, 1C, *J* = 25.1 Hz, C-1”’ *C*H_2_-CHF_2_), 50.1 (1C, C-A *C*H_2_), 47.0 (1C, C-4 *C*H), 35.3 (1C, C-3 *C*H), 31.9 (2C, C-3” *C*H_2_ and C-1” *C*H_2_), 30.5 (1C, C-6 *C*H_2_), 29.7 (1C, C-2” *C*H_2_), 28.2 (1C, C-5 *C*H_2_), 23.8 (1C, C-7 *C*H_3_), 22.7 (1C. C-4” *C*H_2_), 18.8 (1C, C-10 *C*H_3_), 14.1 (1C, C-5” *C*H_3_); ^19^F NMR (659 MHz, CDCl_3_) δ -120.17 (t, *J* = 15.3 Hz), -120.25 (t, *J* = 15.3 Hz); ESI-QTOF MS: *m/z* calcd for C_25_H_35_F_2_NO_2_Na^+^: 442.2528 [M+Na]^+^; found: 442.2528 (mass error: 0 ppm).

**Compound 8M**

Reaction route *L*

The reaction was carried out via *reaction pathway I*.

Reagents: 2-fluoroethylamine hydrochloride (299 mg, 3 mmol), formaldehyde (334 μl, 4 mmol); solvent: dioxane (10 ml); cannabidiol (314 mg, 1 mmol); reaction time and temperature: 20 hours at room temperature; column chromatography: hexane/ethyl acetate 97:3; yielded **8M** (112 mg, 28%) as a yellow syrup.

Reaction route *M*

The reaction was carried out via *reaction pathway I*.

Reagents: 2-fluoroethylamine hydrochloride (299 mg, 3 mmol), formaldehyde (334 μl, 4 mmol); solvent: dioxane (30 ml); cannabidiol (314 mg, 1 mmol); reaction time and temperature: 3 hours at 40 °C; column chromatography: hexane/ethyl acetate 97:3; yielded **8M** (212 mg, 53%) as a yellow syrup.

Reaction route *N*

The reaction is carried out via *reaction pathway I*.

Reagents: 2-fluoroethylamine hydrochloride (299 mg, 3 mmol), formaldehyde (334 μl, 4 mmol); solvent: methanol (30 ml); cannabidiol (314 mg, 1 mmol); reaction time and temperature: 1 day at room temperature; column chromatography: hexane/ethyl acetate 97:3; yielded **8M** (140 mg, 35%) as a yellow syrup.

Reaction route *O*

The reaction was carried out via *reaction pathway II*.

Reagents: 2-fluoroethylamine hydrochloride (149+100 mg, 2.5 mmol), NaHCO_3_ (126+84 mg, 2.5 mmol), formaldehyde (2x167 μl, 4 mmol); solvent: methanol (10 ml); cannabidiol (157 mg, 0.5 mmol); reaction time and temperature: 2 days at room temperature; column chromatography: hexane/ethyl-acetate 97:3; yielded **8M** (145 mg, 72%) as a yellow syrup.

**8M:** R_f_ = 0.16 (hexane/acetone 95:5); ^1^H NMR (500 MHz, CDCl_3_) δ 6.27 (s, 1H, aromatic C*H*), 5.92 (s, 1H, O*H*), 5.58 (s, 1H, H-2 C*H*), 4.79 (d, 1H, *J* = 9.9 Hz, H-B C*H*_2_a), 4.70 (d, 1H, *J* = 9.9 Hz, H-B C*H*_2_b), 4.63 (t, 1H, *J* = 5.0 Hz, H-2”’ C*H*_2_a), 4.54 (t, 1H, *J* = 5.0 Hz, H-2”’ C*H*_2_b), 4.48 (s, 1H, H-9 C*H*_2_a), 4.33 (s, 1H, H-9 C*H*_2_b), 3.99 (d, 1H, *J* = 16.3 Hz, H-A C*H*_2_a), 3.96 – 3.91 (m, 1H, H-3 C*H*), 3.87 (d, 1H, *J* = 16.5 Hz, H-A C*H*_2_b), 3.08 – 2.90 (m, 2H, H-1’” C*H*_2_), 2.44 – 2.29 (m, 3H, H-4 C*H* and H-1” C*H*_2_), 2.28 – 2.18 (m, 1H, H-6 C*H*_2_a), 2.13 – 2.05 (m, 1H, H-6 C*H*_2_b), 1.83 – 1.73 (m, 5H, H-5 C*H*_2_ and H-7 C*H*_3_), 1.67 (s, 3H, H-10 C*H*_3_), 1.56 – 1.47 (m, 2H, H-2” C*H*_2_), 1.38 – 1.28 (m, 4H, H-3” C*H*_2_ and H-4” C*H*_2_), 0.89 (t, 3H, *J* = 7.0 Hz, H-5” C*H*_3_); ^13^C NMR (126 MHz, CDCl_3_) δ 154.1, 152.3, 147.5, 139.8 (4C, quat.), 124.5 (1C, C-2 *C*H), 114.6 (1C, quat.), 111.2 (1C, C-9 *C*H_2_), 109.6 (1C, aromatic *C*H), 108.9 (1C, quat.), 82.75 (d, 1C, *J* = 168.5 Hz, *C*H_2_F), 82.2 (1C, C-B *C*H_2_), , 51.70 (d, 1C, *J* = 19.8 Hz, C-1”’ *C*H_2_-CH_2_F), 49.0 (1C, C-A *C*H_2_), 47.0 (1C, C-4 *C*H), 35.3 (1C, C-3 *C*H), 31.9 (2C, C-3” *C*H_2_ and C-1” *C*H_2_), 30.5 (1C, C-6 *C*H_2_), 29.7 (1C, C-2” *C*H_2_), 28.2 (1C, C-5 *C*H_2_), 23.8 (1C, C-7 *C*H_3_), 22.7 (1C. C-4” *C*H_2_), 18.8 (1C, C-10 *C*H_3_), 14.1 (1C, C-5” *C*H_3_); ^19^F NMR (659 MHz, CDCl_3_) δ -220.92 (tt, *J* = 48.3, 27.6 Hz); ESI-QTOF MS: *m/z* calcd for C_25_H_36_FNO_2_Na^+^: 424.2622 [M+Na]^+^; found: 424.2622 (mass error: 0 ppm).

**Compound 9M**

Reaction route *P*

The reaction was carried out via *reaction pathway III*.

Reagents: 3-fluoroaniline (144 µl, 1.5 mmol), formaldehyde (125 μl, 1.5 mmol); solvent: methanol (10 ml); cannabidiol (157 mg, 0.5 mmol); reaction time and temperature: 6 hours at reflux temperature; column chromatography: hexane/ethyl-acetate 98:2; yielded **9M** (174 mg, 77%) as a yellow syrup.

**9M:** R_f_ = 0.40 (hexane/ethyl acetate 9:1); ^1^H NMR (500 MHz, CDCl_3_) δ 7.20 (q, 1H, *J* = 8.2 Hz, aromatic C*H*), 6.82 (d, 1H, *J* = 8.2 Hz, aromatic C*H*), 6.76 (d, 1H, *J* = 11.7 Hz, aromatic C*H*), 6.67 – 6.58 (m, 1H, aromatic C*H*), 6.33 (s, 1H, aromatic C*H*), 5.97 (s, 1H, O*H*), 5.57 (s, 1H, H-2 C*H*), 5.24 (d, 1H, *J* = 10.3 Hz, H-B C*H*_2_a), 5.14 (d, 1H, *J* = 10.3 Hz, H-B C*H*_2_b), 4.54 – 4.46 (m, 2H, H-A C*H*_2_), 4.35 (s, 1H, H-9 C*H*_2_a), 4.27 (s, 1H, H-9 C*H*_2_b), 3.98 – 3.87 (m, 1H, H-3 C*H*), 2.47 – 2.40 (m, 2H, H-1” C*H*_2_), 2.40 – 2.31 (m, 1H, H-4 C*H*), 2.30 – 2.17 (m, 1H, H-6 C*H*_2_a), 2.15 – 2.03 (m, 1H, H-6 C*H*_2_b), 1.83 – 1.75 (m, 5H, H-5 C*H*_2_ and H-7 C*H*_3_), 1.63 – 1.57 (m, 5H, H-10 C*H*_3_ and H-2” C*H*_2_), 1.41 – 1.35 (m, 4H, H-3” C*H*_2_ and H-4” C*H*_2_), 0.93 (t, 3H, *J* = 7.0 Hz, H-5” C*H*_3_); ^13^C NMR (126 MHz, CDCl_3_) δ 164.7, 162.7, 154.4, 152.7, 150.7, 146.9, 139.9, 139.0 (8C, quat.), 130.24 (d, 1C, *J* = 9.8 Hz, aromatic *C*H-CF), 124.3 (1C, C-2 *C*H), 115.1 (1C, quat.), 113.7 (1C, aromatic *C*H), 111.3 (1C, C-9 *C*H_2_), 109.9 (1C, aromatic *C*H), 107.64 (d, 1C, *J* = 21.4 Hz, aromatic *C*H-CF), 105.26 (d, 1C, *J* = 24.5 Hz, aromatic *C*H-CF), 78.6 (1C, C-B *C*H_2_), 48.4 (1C, C-A *C*H_2_), 46.8 (1C, C-4 *C*H), 35.5 (1C, C-3 *C*H), 31.9 (2C, C-3” *C*H_2_ and C-1” *C*H_2_), 30.5 (1C, C-6 *C*H_2_), 29.6 (1C, C-2” *C*H_2_), 28.1 (1C, C-5 *C*H_2_), 23.8 (1C, C-7 *C*H_3_), 22.7 (1C. C-4” *C*H_2_), 18.9 (1C, C-10 *C*H_3_), 14.2 (1C, C-5” *C*H_3_); ^19^F NMR (659 MHz, CDCl_3_) δ -112.27 (dt, *J* = 11.7, 7.4 Hz); ESI-QTOF MS: *m/z* calcd for C_29_H_36_FNO_2_Na^+^: 472.2622 [M+Na]^+^; found: 472.2622 (mass error: 0 ppm).

**Compound 10M**

Reaction route *Q*

The reaction was carried out via *reaction pathway III*.

Reagents: 4-fluoroaniline (142 µl, 1.5 mmol), formaldehyde (125 μl, 1.5 mmol); solvent: methanol (10 ml); cannabidiol (157 mg, 0.5 mmol); reaction time and temperature: 6 hours at reflux temperature; column chromatography: hexane/ethyl acetate 98:2; yielded **10M** (186 mg, 83%) as a yellow syrup.

**10M:** R_f_ = 0.41 (hexane/ethyl acetate 9:1); ^1^H NMR (500 MHz, CDCl_3_) δ 7.08 – 6.99 (m, 2H, aromatic C*H*), 6.99 – 6.91 (m, 2H, aromatic C*H*), 6.32 (s, 1H, aromatic C*H*), 5.95 (s, 1H, O*H*), 5.57 (s, 1H, H-2 C*H*), 5.18 (d, 1H, *J* = 10.3 Hz, H-B C*H*_2_a), 5.10 (d, 1H, *J* = 10.3 Hz, H-B C*H*_2_b), 4.55 – 4.39 (m, 2H, H-A C*H*_2_), 4.32 (d, 2H, *J* = 31.7 Hz, H-9 C*H*_2_), 3.98 – 3.87 (m, 1H, H-3 C*H*), 2.48 – 2.34 (m, 3H, H-4 C*H* and H-1” C*H*_2_), 2.30 – 2.17 (m, 1H, H-6 C*H*_2_a), 2.15 – 2.03 (m, 1H, H-6 C*H*_2_b), 1.84 – 1.75 (m, 5H, H-5 C*H*_2_ and H-7 C*H*_3_), 1.63 – 1.53 (m, 5H, H-10 C*H*_3_ and H-2” C*H*_2_), 1.42 – 1.32 (m, 4H, H-3” C*H*_2_ and H-4” C*H*_2_), 0.92 (t, 3H, *J* = 7.0 Hz, H-5” C*H*_3_); ^13^C NMR (126 MHz, CDCl_3_) δ 159.2, 157.3, 154.3, 152.7, 147.0, 145.6, 139.8, 138.9 (8C, quat.), 124.4 (1C, C-2 *C*H), 120.87 (d, 1C, *J* = 7.6 Hz, aromatic *C*H-CF), 115.61 (d, 1C, *J* = 22.2 Hz, aromatic *C*H-CF), 115.0 (1C, quat.), 111.3 (1C, C-9 *C*H_2_), 109.8 (1C, aromatic *C*H), 80.0 (1C, C-B *C*H_2_), 49.0 (1C, C-A *C*H_2_), 46.8 (1C, C-4 *C*H), 35.4 (1C, C-3 *C*H), 31.9 (2C, C-3” *C*H_2_ and C-1” *C*H_2_), 30.5 (1C, C-6 *C*H_2_), 29.6 (1C, C-2” *C*H_2_), 28.2 (1C, C-5 *C*H_2_), 23.8 (1C, C-7 *C*H_3_), 22.7 (1C. C-4” *C*H_2_), 18.8 (1C, C-10 *C*H_3_), 14.2 (1C, C-5” *C*H_3_); ^19^F NMR (659 MHz, CDCl_3_) δ -122.41 (tt, *J* = 8.6, 4.6 Hz); ESI-QTOF MS: *m/z* calcd for C_29_H_36_FNO_2_Na^+^: 472.2622 [M+Na]^+^; found: 472.2620 (mass error: 0.4 ppm).

***Synthesis of CBG derivatives***

**Compounds 11D** **and 11M**

Reaction route *A*

The reaction was carried out via *reaction pathway I*.

Reagents: 2,2,2-trifluoroethylamine hydrochloride (135 mg, 1 mmol), formaldehyde (167 μl, 2 mmol); solvent: dioxane (10 ml); cannabigerol (158 mg, 0.5 mmol); reaction time and temperature: 6 hours at reflux temperature; column chromatography: hexane/ethyl acetate 99:1; yielded **11D** (109 mg, 39%) as a yellowish powder.

Reaction route *B*:

The reaction was carried out via *reaction pathway II*.

Reagents: 2,2,2-trifluoroethylamine hydrochloride (407 mg, 3 mmol), NaHCO_3_ (252 mg, 3 mmol), formaldehyde (250 μl, 3 mmol); solvent: methanol (10 ml); cannabigerol (316 mg, 1 mmol); reaction time and temperature: 6 hours at reflux temperature; column chromatography: hexane/ethyl acetate 98:2; yielded **11M** (261 mg, 59%) as a white powder.

Reaction route *C*

The reaction was carried out via *reaction pathway III*.

Reagents: 2,2,2-trifluoroethylamine (2x78 μl, 2 mmol), formaldehyde (167 μl, 2 mmol); solvent: methanol (20 ml); cannabigerol (158 mg, 0.5 mmol); reaction time and temperature: 3 days at reflux temperature during the days and 40 °C at nights; column chromatography: hexane/acetone 95:5; yielded **11M** (74 mg, 34%) as a white powder.

**11D:** R_f_ = 0.75 (hexane/acetone 9:1); ^1^H NMR (400 MHz, CDCl_3_) δ 5.24 – 5.15 (m, 1H, H-2’C*H*), 5.12 – 5.04 (m, 1H, H-6’ C*H*), 4.82 (s, 4H, H-B C*H*_2_), 4.05 (s, 4H, H-A C*H*_2_), 3.35 – 3.21 (m, 6H, H-1’” C*H*_2_ and H-1’ C*H*_2_), 2.31 – 2.19 (m, 2H, H-1” C*H*_2_), 2.10 – 2.01 (m, 2H, H-5’ C*H*_2_), 2.01 – 1.92 (m, 2H, H-4’ C*H*_2_), 1.75 (s, 3H, H-9’ C*H*_3_), 1.66, 1.59 (2s, 6H, H-8’ and H-10’ C*H*_3_), 1.37 (s, 6H, H-2” C*H*_2_, H-3” C*H*_2_ and H-4” C*H*_2_), 0.97 – 0.86 (m, 3H, C-5” C*H*_3_); ^13^C NMR (101 MHz, CDCl_3_) δ 151.3, 136.2, 135.1, 131.3, 129.7, 127.0 (6C, quat.), 124.6 (1C, C-6’ *C*H), 124.2 (1C, quat.), 122.5 (1C, C-2’ *C*H), 115.2 (1C, quat.), 110.1 (1C, quat.), 82.9 (2C, C-B *C*H_2_), 53.42 (q, 2C, *J* = 31.7 Hz, C-1”’ *C*H_2_-CF_3_), 50.4 (2C, C-A *C*H_2_), 40.0 (1C, C-4’ *C*H_2_), 32.3 (1C, C-3” *C*H_2_), 29.4 (1C, C-2” *C*H_2_), 27.5 (1C, C-1” *C*H_2_), 26.9 (1C, C-5’ *C*H_2_), 25.8 (1C, C-8’ *C*H_3_ or C-10’ *C*H_3_), 22.6 (1C, C-4” *C*H_2_), 21.6 (1C, C-1’ *C*H_2_), 17.8 (1C, C-8’ *C*H_3_ or C-10’ *C*H_3_), 16.2 (1C, C-9’ *C*H_3_), 14.0 (1C, C-5” *C*H_3_); ^19^F NMR (659 MHz, CDCl_3_) δ -71.53 (t, *J* = 9.4 Hz); ESI-QTOF MS: *m/z* calcd for C_29_H_40_F_6_N_2_O_2_Na^+^: 585.2886 [M+Na]^+^; found: 585.2869 (mass error: 2.9 ppm).

**11M:** R_f_ = 0.41 (hexane/acetone 9:1); ^1^H NMR (500 MHz, CDCl_3_) δ 6.32 (s, 1H, aromatic C*H*), 5.29 – 5.22 (m, 2H, O*H* and H-2’C*H*), 5.11 – 5.04 (m, 1H, H-6’ C*H*), 4.82 (s, 2H, H-B C*H*_2_), 4.04 (s, 2H, H-A C*H*_2_), 3.36 (d, 2H, *J* = 7.1 Hz, H-1’ C*H*_2_), 3.30 (q, 2H, *J* = 9.3 Hz, H-1’” C*H*_2_), 2.40 – 2.32 (m, 2H, H-1” C*H*_2_), 2.15 – 2.08 (m, 2H, H-5’ C*H*_2_), 2.08 – 2.03 (m, 2H, H-4’ C*H*_2_), 1.81 (s, 3H, H-9’ C*H*_3_), 1.69, 1.60 (2s, 6H, H-8’ and H-10’ C*H*_3_), 1.56 – 1.47 (m, 2H, H-2” C*H*_2_), 1.40 – 1.31 (m, 4H, H-3” C*H*_2_ and H-4” C*H*_2_), 0.95 – 0.88 (m, 3H, H-5” C*H*_3_); ^13^C NMR (126 MHz, CDCl_3_) δ 154.2, 152.1, 139.7, 138.3, 132.0, 126.7, 124.5 (7C, quat.), 124.1 (1C, C-6’ *C*H), 122.1 (1C, C-2’ *C*H), 112.3 (1C, quat), 109.4 (1C quat. and 1C aromatic *C*H), 83.1 (1C, C-B *C*H_2_), 53.57 (q, 1C, *J* = 31.9 Hz, C-1”’ *C*H_2_-CF_3_), 50.5 (1C, C-A *C*H_2_), 39.9 (1C, C-4’ *C*H_2_), 31.9 (2C, C-3” *C*H_2_ and C-1” *C*H_2_), 29.9 (1C, C-2” *C*H_2_), 26.7 (1C, C-5’ *C*H_2_), 25.8 (1C, C-8’ *C*H_3_ or C-10’ *C*H_3_), 22.7 (1C, C-4” *C*H_2_), 22.0 (1C, C-1’ *C*H_2_), 17.7 (1C, C-8’ *C*H_3_ or C-10’ *C*H_3_), 16.3 (1C, C-9’ *C*H_3_), 14.1 (1C, C-5” *C*H_3_); ^19^F NMR (659 MHz, CDCl_3_) δ -71.61 (t, *J* = 9.4 Hz); ESI-QTOF MS: *m/z* calcd for C_25_H_36_F_3_NO_2_Na^+^: 462.2590 [M+Na]^+^; found: 462.2590 (mass error: 0 ppm).

**Compounds 12D and 12M**

Reaction route D

The reaction was carried out via *reaction pathway I*.

Reagents: 2,2-difluoroethylamine hydrochloride (470 mg, 4 mmol), formaldehyde (334 μl, 4 mmol); solvent: dioxane (30 ml); cannabigerol (316 mg, 1 mmol); reaction time and temperature: 1 hour at reflux temperature; column chromatography: hexane/ethyl acetate 97:3; yielded **12D** (203 mg, 39%) as a yellow syrup.

Reaction route *E*

The reaction was carried out via *reaction pathway III*.

Reagents: 2,2-difluoroethylamine (141 µl, 2 mmol), formaldehyde (167 μl, 2 mmol); solvent: methanol (10 ml); cannabigerol (158 mg, 0.5 mmol); reaction time and temperature: 22 hours at 70°C; column chromatography: hexane/ethyl acetate 97:3; yielded **12M** (133 mg, 63%) as a yellow syrup.

Reaction route *F*

The reaction was carried out via *reaction pathway II*.

Reagents: 2,2-difluoroethylamine hydrochloride (470 mg, 4 mmol), NaHCO_3_ (336 mg, 4 mmol), formaldehyde (334 μl, 4 mmol); solvent: methanol (30 ml); cannabigerol (316 mg, 1 mmol); reaction time and temperature: 3 hours at reflux temperature; column chromatography: hexane/ethyl acetate 97:3; yielded **12M** (224 mg, 53%) as a yellow syrup.

**12D:** R_f_ = 0.37 (hexane/acetone 9:1); ^1^H NMR (500 MHz, CDCl_3_) δ 5.94 (tt, 2H, *J* = 56.1, 4.3 Hz, H-2”’ C*H*), 5.25 – 5.19 (m, 1H, H-2’C*H*), 5.13 – 5.06 (m, 1H, H-6’ C*H*), 4.81 (s, 4H, H-B C*H*_2_), 4.01 (s, 4H, H-A C*H*_2_), 3.27 (d, 2H, *J* = 7.2 Hz, H-1’ C*H*_2_), 3.11 (td, 4H, J = 14.9, 4.3 Hz, H-1’” C*H*_2_), 2.30 – 2.21 (m, 2H, H-1” C*H*_2_), 2.11 – 2.04 (m, 2H, H-5’ C*H*_2_), 2.02 – 1.94 (m, 2H, H-4’ C*H*_2_), 1.76 (s, 3H, H-9’ C*H*_3_), 1.67, 1.60 (2s, 6H, H-8’ and H-10’ C*H*_3_), 1.38 (s, 6H, H-2” C*H*_2_, H-3” C*H*_2_ and H-4” C*H*_2_), 0.94 (t, 3H, *J* = 6.9 Hz, C-5” *C*H_3_); ^13^C NMR (126 MHz, CDCl_3_) δ 151.2, 136.0, 134.9, 131.2 (4C, quat.), 124.6 (1C, C-6’ *C*H), 122.6 (1C, C-2’ *C*H), 116.32 (t, 1C, *J* = 241.1 Hz, *C*HF_2_), 114.8, 110.2 (2C, quat.), 82.9 (2C, C-B *C*H_2_), 54.24 (t, 2C, *J* = 25.0 Hz, C-1”’ *C*H_2_-CHF_2_), 49.9 (2C, C-A *C*H_2_), 39.9 (1C, C-4’ *C*H_2_), 32.4 (1C, C-3” *C*H_2_), 29.2 (1C, C-2” *C*H_2_), 27.5 (1C, C-1” *C*H_2_), 26.9 (1C, C-5’ *C*H_2_), 25.8 (1C, C-8’ *C*H_3_ or C-10’ *C*H_3_), 22.6 (1C, C-4” *C*H_2_), 21.6 (1C, C-1’ *C*H_2_), 17.7 (1C, C-8’ *C*H_3_ or C-10’ *C*H_3_), 16.1 (1C, C-9’ *C*H_3_), 14.1 (1C, C-5” *C*H_3_); ^19^F NMR (659 MHz, CDCl_3_) δ -120.17 (t, *J* = 14.8 Hz), -120.25 (t, *J* = 14.8 Hz); ESI-QTOF MS: *m/z* calcd for C_29_H_42_F_4_N_2_O_2_Na^+^: 549.3075 [M+Na]^+^; found: 549.3072 (mass error: 0.5 ppm).

**12M:** R_f_ = 0.32 (hexane/acetone 9:1); ^1^H NMR (500 MHz, CDCl_3_) δ 6.31 (s, 1H, aromatic C*H*), 5.94 (tt, 1H, *J* = 56.0, 4.3 Hz, H-2”’ C*H*), 5.31 – 5.21 (m, 2H, O*H* and H-2’C*H*), 5.12 – 5.04 (m, 1H, H-6’ C*H*), 4.81 (s, 2H, H-B C*H*_2_), 3.99 (s, 2H, H-A C*H*_2_), 3.36 (d, 2H, *J* = 7.2 Hz, H-1’ C*H*_2_), 3.10 (td, 2H, *J* = 14.9, 4.3 Hz, H-1’” C*H*_2_), 2.40 – 2.32 (m, 2H, H-1” C*H*_2_), 2.15 – 2.08 (m, 2H, H-5’ C*H*_2_), 2.07 – 2.02 (m, 2H, H-4’ C*H*_2_), 1.80 (s, 3H, H-9’ C*H*_3_), 1.69, 1.60 (2s, 6H, H-8’ and H-10’ C*H*_3_), 1.57 – 1.46 (m, 2H, H-2” C*H*_2_), 1.41 – 1.30 (m, 4H, H-3” C*H*_2_ and H-4” C*H*_2_), 0.91 (t, 3H, *J* = 6.7 Hz, H-5” C*H*_3_); ^13^C NMR (126 MHz, CDCl_3_) δ 154.0, 152.0, 139.6, 138.2, 132.0 (5C, quat.), 124.0 (1C, C-6’ *C*H), 122.1 (1C, C-2’ *C*H), 116.25 (t, 1C, *J* = 241.0 Hz, *C*HF_2_), 112.2, 109.6 (2C, quat.), 109.3 (1C, aromatic *C*H), 83.1 (1C, C-B *C*H_2_), 54.36 (t, 1C, *J* = 25.1 Hz, C-1”’ *C*H_2_-CHF_2_), 50.0 (1C, C-A *C*H_2_), 39.9 (1C, C-4’ *C*H_2_), 31.9 (2C, C-3” *C*H_2_ and C-1” *C*H_2_), 29.9 (1C, C-2” *C*H_2_), 26.6 (1C, C-5’ *C*H_2_), 25.8 (1C, C-8’ *C*H_3_ or C-10’ *C*H_3_), 22.7 (1C, C-4” *C*H_2_), 22.0 (1C, C-1’ *C*H_2_), 17.8 (1C, C-8’ *C*H_3_ or C-10’ *C*H_3_), 16.3 (1C, C-9’ *C*H_3_), 14.1 (1C, C-5” *C*H_3_); ^19^F NMR (659 MHz, CDCl_3_) δ -120.32 (t, *J* = 15.3 Hz),
-120.40 (t, *J* = 14.8 Hz); ESI-QTOF MS: *m/z* calcd for C_25_H_37_F_2_NO_2_Na^+^: 444.2685 [M+Na]^+^; found: 444.2684 (mass error: 0.2 ppm).

**Compounds 13D and 13M**

Reaction route *G*

The reaction was carried out via *reaction pathway I*.

Reagents: 2-fluoroethylamine hydrochloride (398 mg, 4 mmol), formaldehyde (668 μl, 8 mmol); solvent: dioxane (30 ml); cannabigerol (316 mg, 1 mmol); reaction time and temperature: 2 hours at reflux temperature; column chromatography: hexane/ethyl acetate 9:1; yielded: **13M** (40 mg, 8%) as yellowish powder.

Reaction route *H*

The reaction was carried out via *reaction pathway II*.

Reagents: 2-fluoroethylamine hydrochloride (199 mg, 2 mmol), NaHCO_3_ (168 mg, 2 mmol), formaldehyde (334 μl, 4 mmol); solvent: dioxane (10 ml); cannabigerol (158 mg, 0.5 mmol); reaction time and temperature: 6 hours at reflux temperature; column chromatography: hexane/ethyl acetate 95:5; yielded **13M** (71 mg, 35%) as yellowish powder and **13D** (77mg, 31%) as yellowish syrup.

Reaction route *I*

The reaction was carried out via *reaction pathway I*.

Reagents: 2-fluoroethylamine hydrochloride (398 mg, 4 mmol), formaldehyde (668 μl, 8 mmol); solvent: methanol (30 ml); cannabigerol (316 mg, 1 mmol); reaction time and temperature: 1 day at room temperature; column chromatography: hexane/ethyl acetate 95:5; yielded **13M** (230 mg, 57%) yellowish powder.

Reaction route *J*

The reaction was carried out via *reaction pathway II*.

Reagents: 2-fluoroethylamine hydrochloride (199 mg, 2 mmol), NaHCO_3_ (168 mg, 2 mmol), formaldehyde (334 μl, 4 mmol); solvent: methanol (10 ml); cannabigerol (158 mg, 0.5 mmol); reaction time and temperature: 7 hours at room temperature; column chromatography: hexane/ethyl acetate 95:5; yielded **13M** (118 mg, 58%) as yellowish powder and **13D** (28 mg, 11%) as a yellowish syrup.

Reaction route *K*

The reaction was carried out via *reaction pathway II*.

Reagents: 2-fluoroethylamine hydrochloride (299 mg, 3 mmol), NaHCO_3_ (252 mg, 3 mmol), formaldehyde (250 μl, 3 mmol); solvent: methanol (10 ml); cannabigerol (316 mg, 1 mmol); reaction time and temperature: 6 hours at reflux temperature; column chromatography: hexane/ethyl acetate 95:5; yielded **13M** (202 mg, 50%) as a yellowish powder and **13D** (35 mg, 7%) as a yellowish syrup.

**13D:** R_f_ = 0.39 (hexane/ethyl acetate 8:2); ^1^H NMR (400 MHz, CDCl_3_) δ 5.24 – 5.17 (m, 1H, H-2’C*H*), 5.11 – 5.04 (m, 1H, H-6’ C*H*), 4.83 (s, 4H, H-B C*H*_2_), 4.66 (t, 2H, *J* = 4.9 Hz, H-2”’ C*H_2_*), 4.54 (t, 2H, *J* = 5.0 Hz, H-2”’ C*H_2_*), 4.00 (s, 4H, H-A C*H*_2_), 3.24 (d, 2H, *J* = 7.2 Hz, H-1’ C*H*_2_), 3.09 (t, 2H, *J* = 5.0 Hz, H-1”’ C*H_2_*), 3.02 (t, 2H, *J* = 5.0 Hz, H-1”’ C*H_2_*), 2.30 – 2.22 (m, 2H, H-1” C*H*_2_), 2.11 – 2.00 (m, 2H, H-5’ C*H*_2_), 1.98 – 1.91 (m, 2H, H-4’ C*H*_2_), 1.73 (s, 3H, H-9’ C*H*_3_), 1.65, 1.58 (2s, 6H, H-8’ and H-10’ C*H*_3_), 1.41 – 1.30 (m, 6H, H-2” C*H*_2_, H-3” C*H*_2_ and H-4” C*H*_2_), 0.96 – 0.86 (m, 3H, H-5” C*H*_3_); ^13^C NMR (101 MHz, CDCl_3_) δ 151.1, 135.9, 134.8, 131.2 (4C, quat.), 124.7 (1C, C-6’ *C*H), 122.8 (1C, C-2’ *C*H), 114.5, 110.2 (2C, quat.), 82.90 (d, 1C, *J* = 168.3 Hz, *C*H_2_F), 82.3 (1C, C-B *C*H_2_), 51.85 (d, 2C, *J* = 19.9 Hz, C-1”’ *C*H_2_-CH_2_F), 49.0 (1C, C-A *C*H_2_), 40.0 (1C, C-4’ *C*H_2_), 32.4 (1C, C-3” *C*H_2_), 29.2 (1C, C-2” *C*H_2_), 27.6 (1C, C-1” *C*H_2_), 26.9 (1C, C-5’ *C*H_2_), 25.8 (1C, C-8’ *C*H_3_ or C-10’ *C*H_3_), 22.6 (1C, C-4” *C*H_2_), 21.6 (1C, C-1’ *C*H_2_), 17.8 (1C, C-8’ *C*H_3_ or C-10’ *C*H_3_), 16.2 (1C, C-9’ *C*H_3_), 14.1 (1C, C-5” *C*H_3_); ^19^F NMR (659 MHz, CDCl_3_) δ -220.70 (tt, *J* = 47.4, 27.6 Hz); ESI-QTOF MS: *m/z* calcd for C_29_H_44_F_2_N_2_O_2_Na^+^: 513.3263 [M+Na]^+^; found: 513.3262 (mass error: 0.2 ppm).

**13M:** R_f_ = 0.45 (hexane/ethyl acetate 8:2); ^1^H NMR (500 MHz, CDCl_3_) δ 6.30 (s, 1H, aromatic C*H*), 5.38 (bs, 1H, O*H*), 5.29 – 5.21 (m,1H, H-2’C*H*), 5.12 – 5.03 (m, 1H, H-6’ C*H*), 4.85 (s, 2H, H-B C*H*_2_), 4.66 (t, 1H, *J* = 5.0 Hz, H-2”’ C*H*_2_a), 4.57 (t, 1H, *J* = 5.0 Hz, H-2”’ C*H*_2_b), 3.99 (s, 2H, H-A C*H*_2_), 3.36 (d, 2H, *J* = 7.1 Hz, H-1’ C*H*_2_), 3.07 (dt, 2H, *J* = 27.3, 5.0 Hz, H-1”’ C*H_2_*), 2.41 – 2.33 (m, 2H, H-1” C*H*_2_), 2.15 – 2.07 (m, 2H, H-5’ C*H*_2_), 2.06 – 2.01 (m, 2H, H-4’ C*H*_2_), 1.80 (s, 3H, H-9’ C*H*_3_), 1.68, 1.60 (2s, 6H, H-8’ and H-10’ C*H*_3_), 1.57 – 1.47 (m, 2H, H-2” C*H*_2_), 1.41 – 1.29 (m, 4H, H-3” C*H*_2_ and H-4” C*H*_2_), 0.96 – 0.86 (m, 3H, H-5” C*H*_3_); ^13^C NMR (126 MHz, CDCl_3_) δ 153.9, 152.1, 139.5, 137.9, 131.9 (5C, quat.), 124.1 (1C, C-6’ *C*H), 122.2 (1C, C-2’ *C*H), 112.1 (1C, quat.), 109.8 (1C, quat.), 109.1 (1C, aromatic *C*H), 82.82 (d, 1C, *J* = 168.2 Hz, *C*H_2_F), 82.6 (1C, C-B *C*H_2_), 51.94 (d, 1C, *J* = 20.2 Hz, C-1”’ *C*H_2_-CH_2_F), 49.0 (1C, C-A *C*H_2_), 39.9 (1C, C-4’ *C*H_2_), 31.9 (2C, C-3” *C*H_2_ and C-1” *C*H_2_), 29.9 (1C, C-2” *C*H_2_), 26.6 (1C, C-5’ *C*H_2_), 25.8 (1C, C-8’ *C*H_3_ or C-10’ *C*H_3_), 22.7 (1C, C-4” *C*H_2_), 22.0 (1C, C-1’ *C*H_2_), 17.8 (1C, C-8’ *C*H_3_ or C-10’ *C*H_3_), 16.2 (1C, C-9’ *C*H_3_), 14.1 (1C, C-5” *C*H_3_); ^19^F NMR (659 MHz, CDCl_3_) δ -220.78 (tt, *J* = 47.4, 26.8 Hz); ESI-QTOF MS: *m/z* calcd for C_25_H_38_FNO_2_Na^+^: 426.2779 [M+Na]^+^; found: 426.2778 (mass error: 0.2 ppm).

**Compound 14M**

Reaction route *L*

The reaction was carried out via *reaction pathway III*.

Reagents: 3-fluoroaniline (144 µl, 1.5 mmol), formaldehyde (125 μl, 1.5 mmol); solvent: methanol (10 ml); cannabigerol (158 mg, 0.5 mmol); reaction time and temperature: 6 hours at reflux temperature and 15 hours at 40 °C; column chromatography: hexane/acetone 98:2; yielded **14M** (190 mg, 84%) as a yellow powder.

**14M:** R_f_ = 0.29 (hexane/acetone 8:2); ^1^H NMR (500 MHz, CDCl_3_) δ 7.25 – 7.17 (m, 1H, aromatic C*H*), 6.90 – 6.84 (m, 1H, aromatic C*H*), 6.84 – 6.77 (m, 1H, aromatic C*H*), 6.66 – 6.58 (m, 1H, aromatic C*H*), 6.34 (s, 1H, aromatic C*H*), 5.34 – 5.29 (m, 3H, O*H* and H-B C*H*_2_), 5.28 – 5.23 (m, 1H, H-2’C*H*), 5.12 – 5.05 (m, 1H, H-6’ C*H*), 4.53 (s, 2H, H-A C*H*_2_), 3.37 (d, 2H, *J* = 7.1 Hz, H-1’ C*H*_2_), 2.45 (t, 2H, *J* = 7.8 Hz, H-1” C*H*_2_), 2.16 – 2.08 (m, 2H, H-5’ C*H*_2_), 2.08 – 2.02 (m, 2H, H-4’ C*H*_2_), 1.81 (s, 3H, H-9’ C*H*_3_), 1.70 (s, 3H, H-8’ or H-10’ C*H*_3_), 1.64 – 1.54 (m, 5H, H-8’ or H-10’ C*H*_3_ and H-2” C*H*_2_), 1.43 – 1.35 (m, 4H, H-3” C*H*_2_ and H-4” C*H*_2_), 0.99 – 0.90 (m, 3H, H-5” C*H*_3_); ^13^C NMR (126 MHz, CDCl_3_) δ 164.8, 162.8, 154.0, 152.3, 150.5, 150.4, 138.7, 138.1, 131.9 (9C, quat.), 130.40 (d, 1C, *J* = 9.8 Hz, aromatic *C*H-CF), 124.0 (1C, C-6’ *C*H), 122.0 (1C, C-2’ *C*H), 113.3 (1C, aromatic *C*H), 112.5, 110.7 (2C, quat.), 109.3 (1C, aromatic *C*H), 107.66 (d, 1C, *J* = 21.3 Hz, aromatic *C*H-CF), 105.01 (d, 1C, *J* = 24.7 Hz, aromatic *C*H-CF), 78.5 (1C, C-B *C*H_2_), 48.5 (1C, C-A *C*H_2_), 39.8 (1C, C-4’ *C*H_2_), 31.9 (C-3” *C*H_2_ and C-1” *C*H_2_), 29.8 (1C, C-2” *C*H_2_), 26.6 (C-5’ *C*H_2_), 25.8 (C-8’ *C*H_3_ or C-10’ *C*H_3_), 22.7 (1C, C-4” *C*H_2_), 22.0 (1C, C-1’ *C*H_2_), 17.8 (1C, C-8’ *C*H_3_ or C-10’ *C*H_3_), 16.3 (1C, C-9’ *C*H_3_), 14.1 (1C, C-5” *C*H_3_); ^19^F NMR (659 MHz, CDCl_3_) δ -112.03 (dt, *J* = 11.7, 7.6 Hz); ESI-QTOF MS: *m/z* calcd for C_29_H_38_FNO_2_Na^+^: 474.2779 [M+Na]^+^; found: 474.2781 (mass error: 0.4 ppm).

**Compounds 15M and 15D**

Reaction route *M*

The reaction was carried out via *reaction pathway III*.

Reagents: 4-fluoroaniline (142 µl, 1.5 mmol), formaldehyde (125 μl, 1.5 mmol); solvent: methanol (10 ml); cannabigerol (158 mg, 0.5 mmol); reaction time and temperature: 6 hours at reflux temperature and 15 hours at 40 °C; column chromatography: hexane/acetone 98:2; yielded **15M** (194 mg, 85%) as a yellow powder and **15D** (44 mg, 15%) as a white powder.

**15D:** R_f_ = 0.37 (hexane/acetone 8:2); ^1^H NMR (400 MHz, CDCl_3_) δ 7.12 – 7.03 (m, 4H, aromatic C*H*), 7.00 – 6.89 (m, 4H, aromatic C*H*), 5.24 (s, 4H, H-B C*H*_2_), 5.21 – 5.14 (m, 1H, H-2’C*H*), 5.14 – 5.06 (m, 1H, H-6’ C*H*), 4.50 (s, 4H, H-A C*H*_2_), 3.26 (d, 2H, *J* = 7.2 Hz, H-1’ C*H*_2_), 2.40 – 2.30 (m, 2H, H-1” C*H*_2_), 2.11 – 2.01 (m, 2H, H-5’ C*H*_2_), 2.00 – 1.91 (m, 2H, H-4’ C*H*_2_), 1.76 (s, 3H, H-9’ C*H*_3_), 1.67 (s, 3H, H-8’ or H-10’ C*H*_3_), 1.60 (s, 3H, H-8’ or H-10’ C*H*_3_), 1.47 – 1.33 (m, 6H, H-2” C*H*_2_, H-3” C*H*_2_ and H-4” C*H*_2_), 0.99 – 0.89 (m, 3H, H-5” C*H*_3_); ^13^C NMR (101 MHz, CDCl_3_) δ 159.4, 157.0, 151.4, 145.5, 134.8, 134.3, 131.3 (7C, quat.), 124.6 (1C, C-6’ *C*H), 122.7 (1C, C-2’ *C*H), 120.63 (d, 1C, *J* = 7.8 Hz, aromatic *C*H-CF), 115.80 (d, 1C, *J* = 22.3 Hz, aromatic *C*H-CF), 115.3, 111.2 (2C, quat.), 79.5 (1C, C-B *C*H_2_), 49.7 (1C, C-A *C*H_2_), 40.0 (1C, C-4’ *C*H_2_), 32.5 (1C, C-3” *C*H_2_), 29.1 (1C, C-2” *C*H_2_), 27.8 (1C, C-1” *C*H_2_), 26.9 (1C, C-5’ *C*H_2_), 25.8 (C-8’ *C*H_3_ or C-10’ *C*H_3_), 22.6 (1C, C-4” *C*H_2_), 21.7 (1C, C-1’ *C*H_2_), 17.8 (1C, C-8’ *C*H_3_ or C-10’ *C*H_3_), 16.3 (1C, C-9’ *C*H_3_), 14.1 (1C, C-5” *C*H_3_); ^19^F NMR (659 MHz, CDCl_3_) δ -122.29 (tt, *J* = 8.7, 4.7 Hz); ESI-QTOF MS: *m/z* calcd for C_37_H_44_F_2_N_2_O_2_Na^+^: 609.3263 [M+Na]^+^; found: 609.3263 (mass error: 0 ppm).

**15M:** R_f_ = 0.24 (hexane/acetone 8:2); ^1^H NMR (400 MHz, CDCl_3_) δ 7.12 – 7.05 (m, 2H, aromatic C*H*), 7.01 – 6.92 (m, 2H, aromatic C*H*), 6.33 (s, 1H, aromatic C*H*), 5.37 – 5.21 (m, 4H, O*H*, H-B C*H*_2_ and H-2’C*H*), 5.13 – 5.04 (m, 1H, H-6’ C*H*), 4.50 (s, 2H, H-A C*H*_2_), 3.37 (d, 2H, *J* = 7.1 Hz, H-1’ C*H*_2_), 2.48 – 2.38 (m, 2H, H-1” C*H*_2_), 2.16 – 2.09 (m, 2H, H-5’ C*H*_2_), 2.09 – 2.02 (m, 2H, H-4’ C*H*_2_), 1.81 (s, 3H, H-9’ C*H*_3_), 1.70 (s, 3H, H-8’ or H-10’ C*H*_3_), 1.64 – 1.52 (m, 5H, H-8’ or H-10’ C*H*_3_ and H-2” C*H*_2_), 1.44 – 1.33 (m, 4H, H-3” C*H*_2_ and H-4” C*H*_2_), 0.98 – 0.90 (m, 3H, H-5” C*H*_3_); ^13^C NMR (101 MHz, CDCl_3_) δ 159.4, 157.0, 153.9, 152.4, 145.3, 138.6, 138.0, 131.9 (8C, quat.), 124.0 (1C, C-6’ *C*H), 122.1 (1C, C-2’ *C*H), 120.56 (d, 1C, *J* = 7.8 Hz, aromatic *C*H-CF), 115.78 (d, 1C, *J* = 22.3 Hz, aromatic *C*H-CF), 112.4, 110.7 (2C, quat.), 109.2 (1C, aromatic *C*H), 79.9 (1C, C-B *C*H_2_), 49.2 (1C, C-A *C*H_2_), 39.8 (1C, C-4’ *C*H_2_), 31.9 (C-3” *C*H_2_ and C-1” *C*H_2_), 29.8 (1C, C-2” *C*H_2_), 26.6 (C-5’ *C*H_2_), 25.8 (C-8’ *C*H_3_ or C-10’ *C*H_3_), 22.7 (1C, C-4” *C*H_2_), 22.0 (1C, C-1’ *C*H_2_), 17.8 (1C, C-8’ *C*H_3_ or C-10’ *C*H_3_), 16.3 (1C, C-9’ *C*H_3_), 14.1 (1C, C-5” *C*H_3_); ^19^F NMR (659 MHz, CDCl_3_) δ -122.32 (tt, *J* = 8.2, 4.5 Hz); ESI-QTOF MS: *m/z* calcd for C_29_H_38_FNO_2_Na^+^: 474.2779 [M+Na]^+^; found: 474.2777 (mass error: 0.4 ppm).

# Antimalarial results

The heatmap analysis of the antimalarial activity of CBD and CBG derivatives allowed for rapid visual differentiation of performance trends with a clear gradient shift indicating declining efficacy in certain compounds at lower concentrations.

**
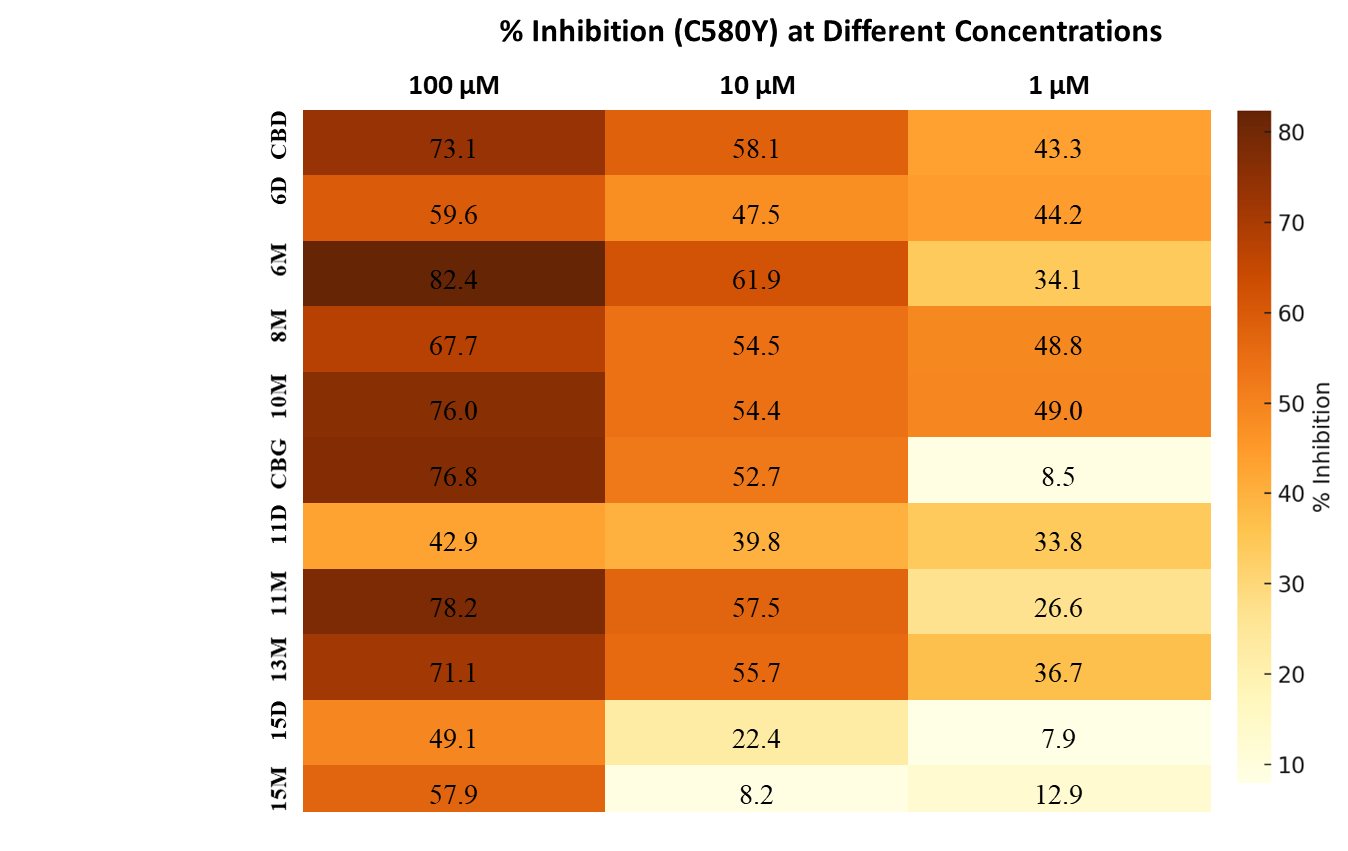
**

**Figure S1**. Heatmap of % inhibition of CBD and CBG derivatives against the *Pf*C580Y strain.

# Antiviral tests

1. SARS-CoV-2 infection, cytopathic effect (CPE) detection

Anti-SARS-CoV-2 activity was tested in VERO-E6 cells (5,000 cells/25 µl) in 384-well plate format. Compounds were tested from 128 µM concentration using 2-fold dilution. Compounds were added to the cells, after one hour, cells were infected with SARS-CoV-2 (MOI 0.01). Cells were incubated for 72 hours in CO_2_ incubator set to 37 °C and after incubation cytopathic effect (CPE) was analyzed by XTT colorimetric assay. Briefly, 50 μl of 50:1 mixture of XTT labeling reagent (1 mg/ml) and PMS electron-coupling reagent (0.383 mg/ml) was added to the wells and incubated for 4 h in 37 °C in 5 % CO_2_. Formation of orange formazan dye was measured in EnVision plate reader.

1. Cytotoxicity in VERO-E6 cells (XTT assay- CC_50_ measurement)

Cytotoxicity was measured in VERO-E6 cells (5,000 cells/25 µl) in 384-well plate format. Compound cytotoxicity was tested from 128 µM concentration using 2-fold dilution. Cytotoxicity was detected after 72 hours using XTT assay.

1. SARS-CoV-2 infection, Immunofluorescence (IF) assay

Anti-SARS-COV-2 activity was tested in VERO-E6 cells (20,000 cells/100 µl) in 96-well plate for those compounds, which have shown measurable antiviral activity in previous CPE detection. Compound was tested from the 100 µM concentration using 2-fold dilution. Compound was added to the cells, after one hour, cells were infected with SARS-CoV-2 (MOI 0.005). Cells were incubated for 72 hours in CO_2_ incubator set to 37 °C and after incubation IF assay was performed. Briefly, medium was washed out, cells were fixed using 4% PFA, cell membranes were perforated with 0.2% Triton-X100 and SARS-CoV-2 nucleoprotein was labeled with 1^st^ mouse anti-SARS-CoV-2 antibody, 2^nd^ anti-mouse antibody conjugated with Cy-3 fluorophore and signal was detected using fluorescent microscope

As a control compound, Remdesivir was used. Tempest liquid dispenser system (Formulatrix) was used for all dispensing steps.

**Table S1.** Anti-SARS-CoV-2 activity and cytotoxicity of fluorine containing CBD and CBG derivatives

| Compound | EC_50_ CPE [µM] | 95% CI of EC_50_ CPE | CC_50_ [µM] | 95% CI of CC_50_ | EC_50_ IF [µM] | 95% CI of EC_50_ IF |
| --- | --- | --- | --- | --- | --- | --- |
| **6D** | 63 | 51 – 77 | >128 | n.a. | ~128 | n.a. |
| **6M** | 9.3 | 8.8 – 9.9 | 29 | 26 – 32 | 17 | 7.5 – 36 |
| **7D** | 74 | 65 – 84 | >128 | n.a. | 37 | 29 – 49 |
| **7M** | 26 | 25 – 28 | 40 | 33 – 47 | >25 | n.a. |
| **8M** | 24 | 23 – 25 | 53 | 51 – 55 | ~11 | n.a. |
| **11D** | >128 | n.a. | >128 | n.a. | n.d. | n.d. |
| **11M** | >17 | n.a. | ~17 | n.a. | n.d. | n.d. |
| **12M** | >28 | n.a. | 28 | 21.0 – 37 | n.d. | n.d. |
| **13D** | 62 | 56 – 69 | 100 | 94 – 110 | 90 | 43 – 190 |
| **13M** | >37 | n.a. | 37 | 35.1 – 38.6 | n.d. | n.d. |
| Remdesivir | 4.7 | 4.2 – 5.1 | >128 | n.a. | 1.5 | 1.1 – 1.9 |

n.d. not determined, n.a. not available

# Antibacterial assay

Reference: Clinical and Laboratory Standards Institute. 2018. Methods for dilution antimicrobial susceptibility tests for bacteria that grow aerobically, 11th ed Approved standard M07-A11 Clinical and Laboratory Standards Institute, Wayne, PA.

According to Clinical and Laboratory Standards Institute (CLSI) guideline the Minimal Inhibitory Concentrations (MICs) of the prepared compounds were determined with the broth micro dilution method. After an overnight growth on 5% bovine blood agar plates at 35 °C bacterial strains were suspended in physiological saline in order to reach the density of 0.5 McFarland for inoculation. Stock solutions containing different concentrations of the substances were prepared in DMSO and H_2_O (1:1). These were two-fold serially diluted from 256 to 0.5 mg/l in Mueller-Hinton broth, and 100 ml of each dilution was inoculated with 10 ml of each bacterial suspension. Incubation was performed at 35 °C for 18 h and determination of MIC was made with the naked eyes on a dark background.

**Table S2.** Antibacterial results of fluorine containing CBD and CBG derivatives.

|  | | VAN | CBD | 6D | 6M | 7D | 7M | 8M | CBG | 11D | 11M | 12M | 13D | 13M |
| --- | --- | --- | --- | --- | --- | --- | --- | --- | --- | --- | --- | --- | --- | --- |
|  | **MIC (µg/ml)** | | | | | | | | | | | | | |
| *Bacillus subtilis* | | 0.5 | 4 | 256 | 256 | 256 | 256 | 256 | 2 | 256 | 256 | 256 | 256 | 256 |
| methicillin-susceptible *Staphylococcus aureus* MSSA | | 0.5 | 4 | 256 | 256 | 256 | 256 | 256 | 4 | 256 | 256 | 256 | 256 | 256 |
| methicillin-resistant *Staphylococcus Aureus* MRSA | | 0.5 | 4 | 256 | 256 | 256 | 256 | 256 | 4 | 256 | 256 | 256 | 256 | 256 |
| *Staphylococcus epidermidis* biof. | | 2 | 8 | 256 | 256 | 256 | 256 | 256 | 8 | 256 | 256 | 256 | 256 | 256 |
| *Staphylococcus epidermidis* mecA | | 4 | 8 | 256 | 256 | 256 | 256 | 256 | 8 | 256 | 256 | 256 | 256 | 256 |
| *Enterococcus faecalis* 29 212 | | 1 | 2 | 256 | 64 | 128 | 256 | 256 | 4 | 64 | 32 | 32 | 256 | 64 |
| *Enterococcus faecalis* 15 376 VanA | | 256 | 4 | 256 | 64 | 128 | 256 | 256 | 4 | 64 | 32 | 32 | 256 | 64 |
| *Enterococcus faecalis* 51299 VanB | | 128 | 4 | 256 | 256 | 256 | 256 | 256 | 4 | 256 | 256 | 256 | 256 | 256 |

MIC: Minimum inhibitory concentration; VAN: vancomycin

# Drug-likeness properties and *in silico* ADMET analysis

**Table S3.** Molecular properties of CBD, CBG, and the newly synthesized compounds **6M**, **8M** and **13M**.

| **Compound** | **ALogP** | **MW (g/mol)** | **Num. HBD** | **Num. HBA** | **Num. RB** |
| --- | --- | --- | --- | --- | --- |
| **CBD** | 6.613 | 314.462 | 2 | 2 | 6 |
| **6M** | 6.160 | 438.546 | 2 | 2 | 8 |
| **8M** | 5.553 | 402.565 | 2 | 2 | 8 |
| **CBG** | 7.340 | 316.478 | 2 | 2 | 9 |
| **13M** | 6.280 | 404.581 | 2 | 2 | 11 |

MW: Molecular weight, Num. HBD: Number of hydrogen bond donors, Num. HBA: Number of hydrogen bond acceptors, Num. RB: Number of rotatable bonds.

**Table S4.** *In silico* ADMET and mutagenic properties of CBD, CBG, and the newly synthesized compounds **6M**, **8M** and **13M**

| **Compounds** | **Absorption level** | **Solubility level** | **BBB level** | **PPB level** | **CYP2D6 inhibition** | **Hepatotoxicity** | **Mutagenicity** | **PSA_2D (Å^2^)** |
| --- | --- | --- | --- | --- | --- | --- | --- | --- |
| **CBD** | 1  (moderate) | 2  (low) | 0  (very high) | 1  (≥ 90%) | 1  (inhibitor) | 0  (not toxic) | 0  (non-mutagenic) | 41.631 |
| **6M** | 1  (moderate) | 1  (very low, but possible) | 0  (very high) | 1  (≥ 90%) | 0  (non-inhibitor) | 0  (not toxic) | 0  (non-mutagenic) | 32.949 |
| **8M** | 0  (good) | 2  (low) | 0  (very high) | 1  (≥ 90%) | 0  (non-inhibitor) | 0  (not toxic) | 0  (non-mutagenic) | 32.949 |
| **CBG** | 3  (very low) | 2  (low) | 4  (undefined) | 1  (≥ 90%) | 1  (inhibitor) | 0  (not toxic) | 0  (non-mutagenic) | 41.631 |
| **13M** | 1  (moderate) | 2  (low) | 0  (very high) | 1  (≥ 90%) | 0  (non-inhibitor) | 0  (not toxic) | 0  (non-mutagenic) | 32.949 |

BBB: Blood-brain barrier, PPB: Plasma protein binding, PSA: Polar surface area.

# **Table S5.** Consolidated potency table.

| Compound | Side chain | Substitution | ALogP | Absorption level | Sebaceous lipogenesis at  30 µM after 24 h  (**red fonts:** alterations with opposing directions compared to the parent compounds) | | Antiproliferative effect after 72 h  % viability | | | | Antiproliferative effect at 30 µM after 48 h % viability | | | | Effect against PfC580Y at 1 µM  % Inhibition | Anti-viral effect  EC_50_ CPE [µM] | CC_50_ on Vero cells [µM]  (**green**: low toxicity, **orange**: moderate  **red**: high) | Antibac-terial effect  MIC [µg/ml] |
| --- | --- | --- | --- | --- | --- | --- | --- | --- | --- | --- | --- | --- | --- | --- | --- | --- | --- | --- |
|  |  |  |  |  | vs. control (control  =100%)  % | vs. 50 μM AA (AA=100%)  % | CAKI-2  at 30 µM | A-498  at 30 µM | CAKI-2  at 60 µM | A-498  at 60 µM | MDA-MB-231 | MCF-7 | OCM-1 | OCM-3 |  |  |  | *Entero-coccus faecalis* 29 212 |
| **CBD** | - | - | 6.613 | moderate | ˗30.3 | ˗54.27 | 13.64 | 16.26 | 11.90 | 13.24 | 34.6 | 88.7 | 43.0 | 47.0 | 43.3 | 5.1 | **12.1** | 2 |
| **6D** |  | Di | 5.707 | good | **+26.9** | **+15.72** | 81.82 | 76.43 | 77.50 | 70.63 | 83.7 | 93.8 | 98.6 | 125.4 | 44.2 | 63 | **>128** | 256 |
| **6M** |  | Mono | 6.160 | moderate | ˗21.3 | ˗37.56 | 34.98 | 79.36 | 15.62 | 42.91 | 36.1 | 95.2 | 99.8 | 94.2 | 34.1 | 9.3 | **29** | 64 |
| **7D** |  | Di | 4.718 | good | **+12.1** | ˗13.69 | 91.64 | 95.55 | 94.22 | 98.67 | 102.1 | 84.1 | 87.9 | 100.7 | n.d. | 74 | **>128** | 128 |
| **7M** |  | Mono | 5.666 | good | ˗8 | ˗24.79 | 100.31 | 87.47 | 43.46 | 82.76 | 102.0 | 116.4 | 105.1 | 113.2 | n.d. | 26 | **40** | 256 |
| **8M** |  | Mono | 5.553 | good | **+0.2** | ˗9.19 | 76.43 | 56.26 | 22.43 | 25.72 | n.d. | n.d. | n.d. | n.d. | 48.8 | 24 | **53** | 256 |
| **9M** |  | Mono | 8.591 | very low | ˗5.4 | ˗17.08 | 76.63 | 82.29 | 68.84 | 73.92 | 73.3 | 100.4 | 72.4 | 84.6 | n.d. | n.d. | n.d. | n.d. |
| **10M** |  | Mono | 8.591 | very low | ˗2 | ˗19.92 | n.d. | n.d. | n.d. | n.d. | n.d. | n.d. | n.d. | n.d. | 49.0 | n.d. | n.d. | n.d. |
| **CBG** | - | - | 7.340 | very low | ˗15.89 | ˗34.73 | 68.68 | 57.03 | 16.08 | 17.13 | 47.3 | 11.0 | 27.6 | 84.0 | 8.5 | >11 | **~11** | 4 |
| **11D** |  | Di | 6.433 | moderate | **+25.89** | **+10.62** | 86.30 | 96.55 | 87.93 | 92.45 | 84.8 | 107.4 | 206.1 | 93.3 | 33.8 | >128 | **>128** | 64 |
| **11M** |  | Mono | 6.887 | low | ˗7.51 | ˗25.04 | 20.52 | 36.42 | 14.85 | 17.94 | 20.9 | 25.9 | 55.2 | 60.6 | 26.6 | >17 | **~17** | 32 |
| **12D** |  | Di | 5.445 | good | n.d. | n.d. | n.d. | n.d. | n.d. | n.d. | n.d. | n.d. | n.d. | n.d. | n.d. | n.d. | n.d. | n.d. |
| **12M** |  | Mono | 6.329 | moderate | n.d. | n.d. | 90.78 | 85.01 | 28.25 | 39.04 | 65.4 | 105.0 | 84.5 | 92.7 | n.d. | >28 | **28** | 32 |
| **13D** |  | Di | 5.220 | good | **+10.03** | **+4.58** | 33.37 | 52.40 | 19.24 | 17.74 | 36.3 | 68.5 | 85.4 | 78.2 | n.d. | 62 | **100** | 256 |
| **13M** |  | Mono | 6.280 | moderate | **+23.71** | **+16.1** | 80.73 | 84.26 | 23.51 | 29.61 | n.d. | n.d. | n.d. | n.d. | 36.7 | >37 | **37** | 64 |
| **14M** |  | Mono | 9.317 | very low | ˗9.96 | ˗29.59 | 94.65 | 92.26 | 94.92 | 88.28 | 45.2 | 43.5 | 60.1 | 68.2 | n.d. | n.d. | n.d. | n.d. |
| **15D** |  | Di | 11.295 | very low | ˗3.1 | ˗12.27 | 46.41 | 56.26 | 16.83 | 24.13 | n.d. | n.d. | n.d. | n.d. | 7.9 | n.d. | n.d. | n.d. |
| **15M** |  | Mono | 9.317 | very low | **+9.72** | ˗16.26 | n.d. | n.d. | n.d. | n.d. | n.d. | n.d. | n.d. | n.d. | 12.9 | n.d. | n.d. | n.d. |

ALogP: Calculated LogP; vs.: versus; AA: Arachidonic Acid; CAKI-2 and A-498: human renal carcinoma cell lines; MDA-MB-231 and MCF-7: human breast cancer cell lines; OCM-1 and OCM-3: human uveal melanoma cell lines; *PfC580Y*: *Plasmodium falciparum* strain; EC_50_: Effective Concentration, 50%; CPE: Cytopathic Effect; CC_50_: Cytotoxicity Concentration, 50%; MIC: Minimum Inhibitory Concentration; n.d.: not determined.

Color map explanation:

| Lowest effect |  |  |  |  |  | Highest effect |
| --- | --- | --- | --- | --- | --- | --- |

# NMR spectra of the CBD and CBG derivatives

**NMR spectra of compound 6D**


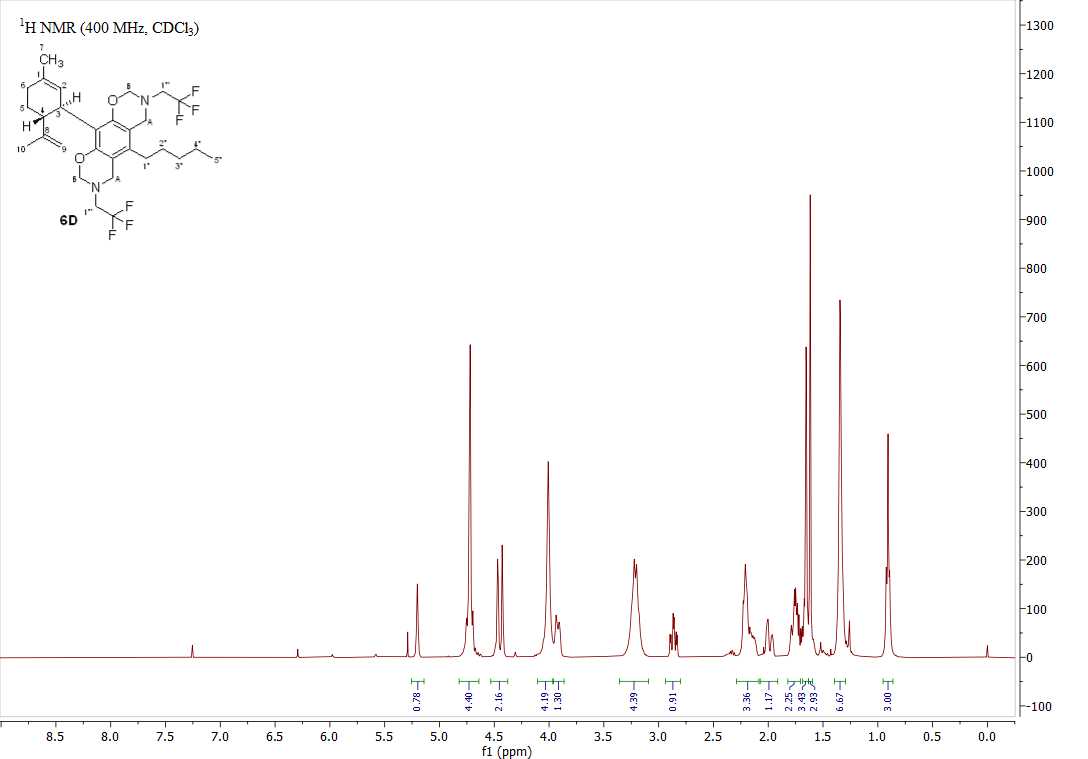


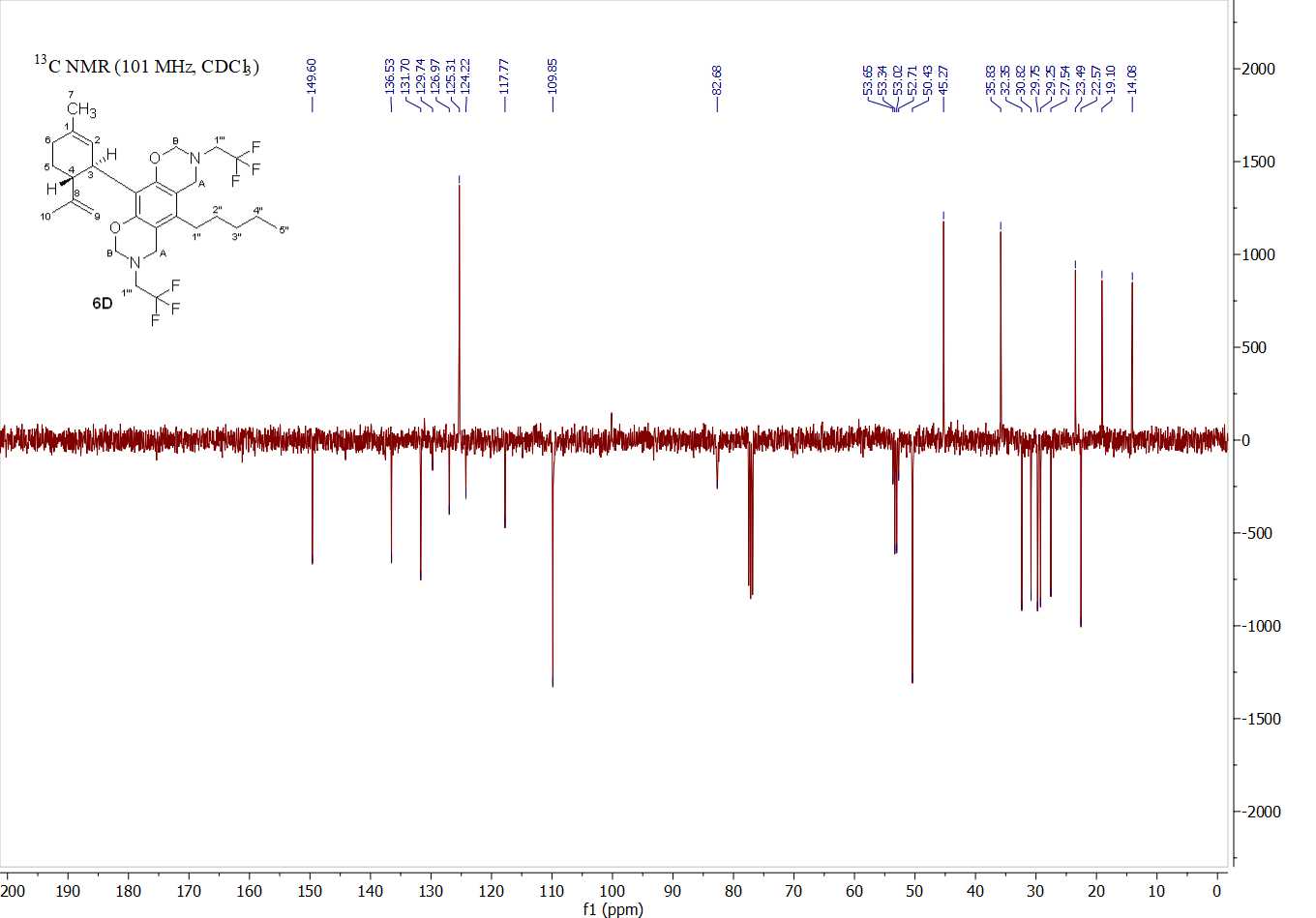


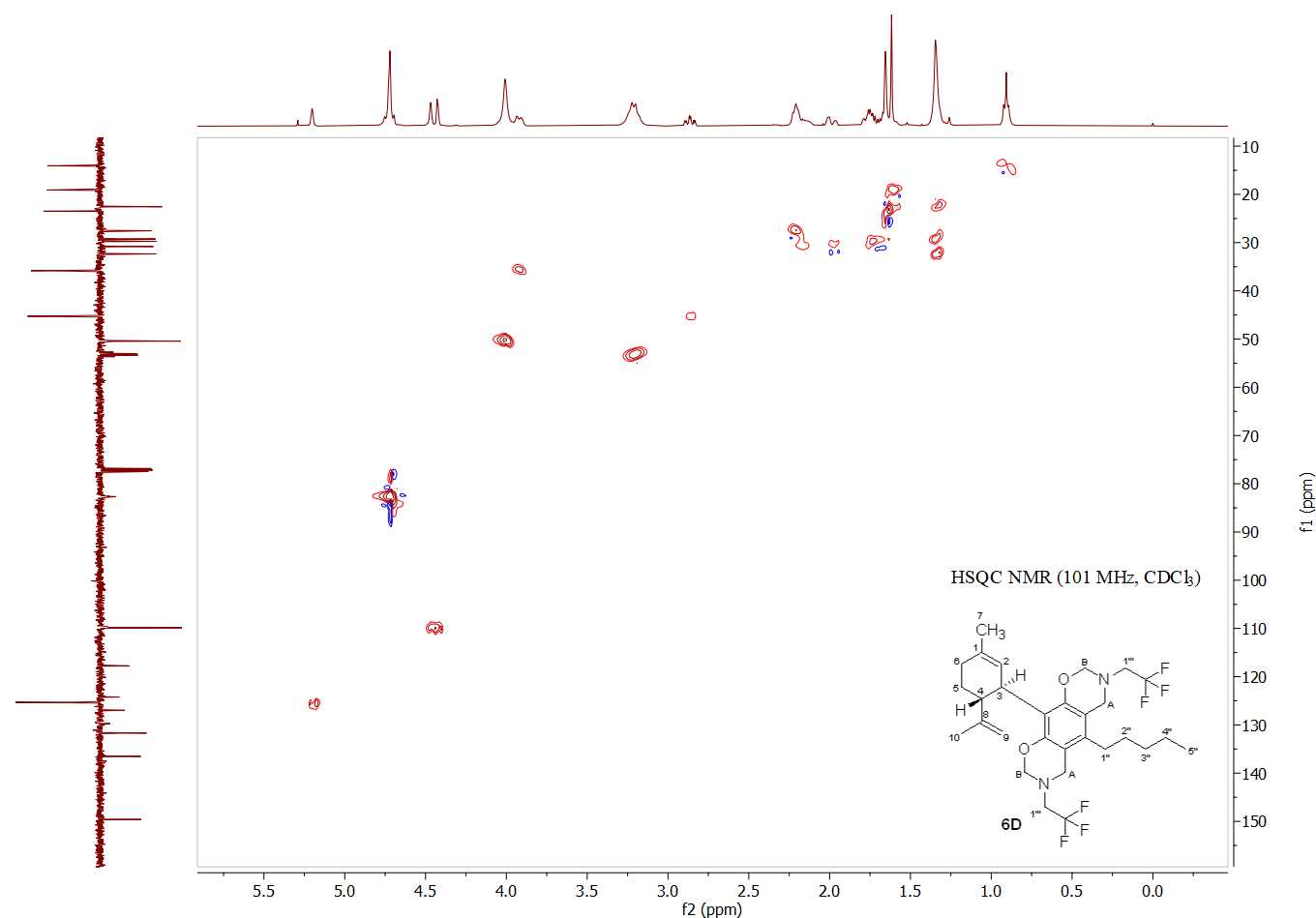


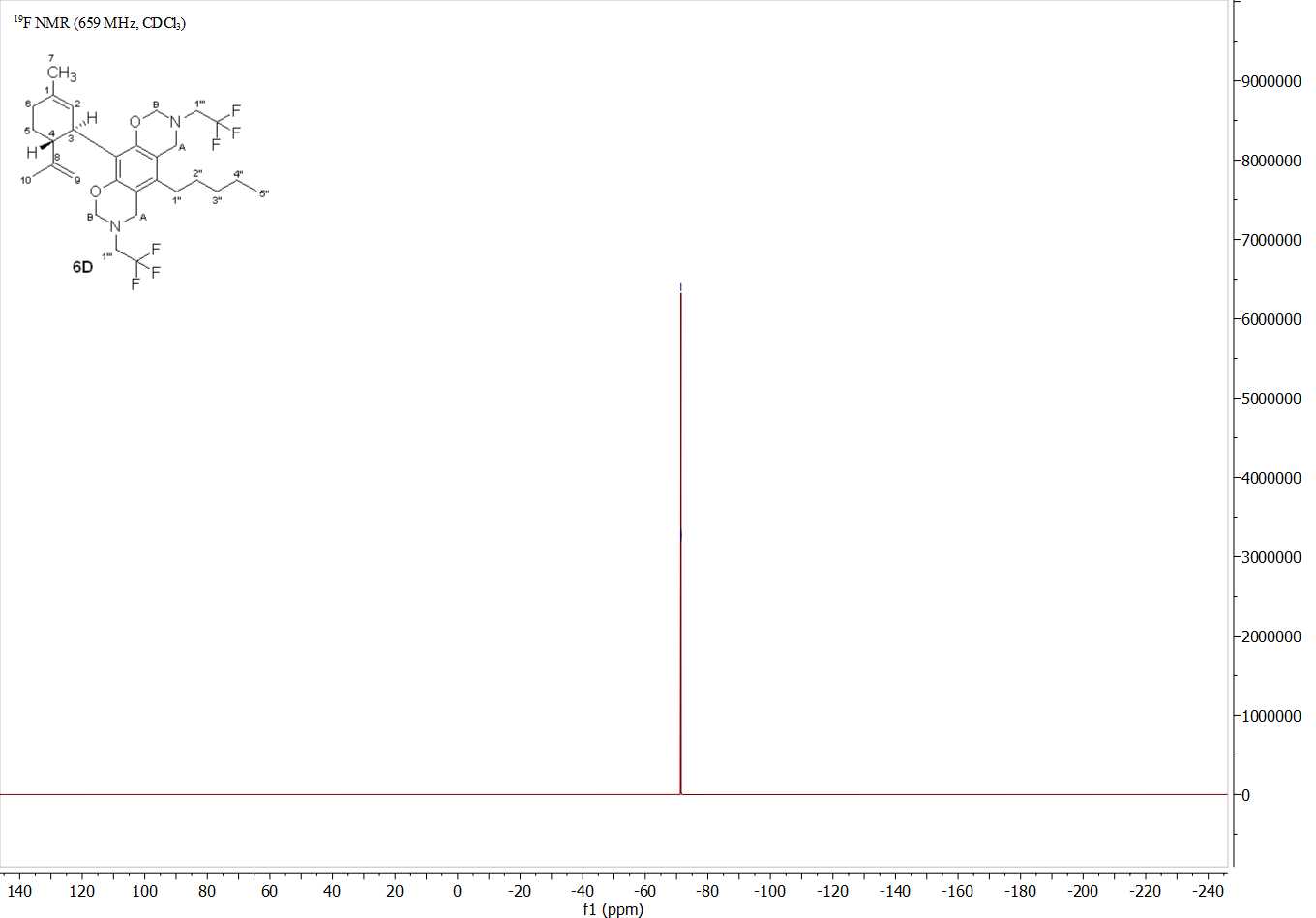


**NMR spectra of compound 6M**


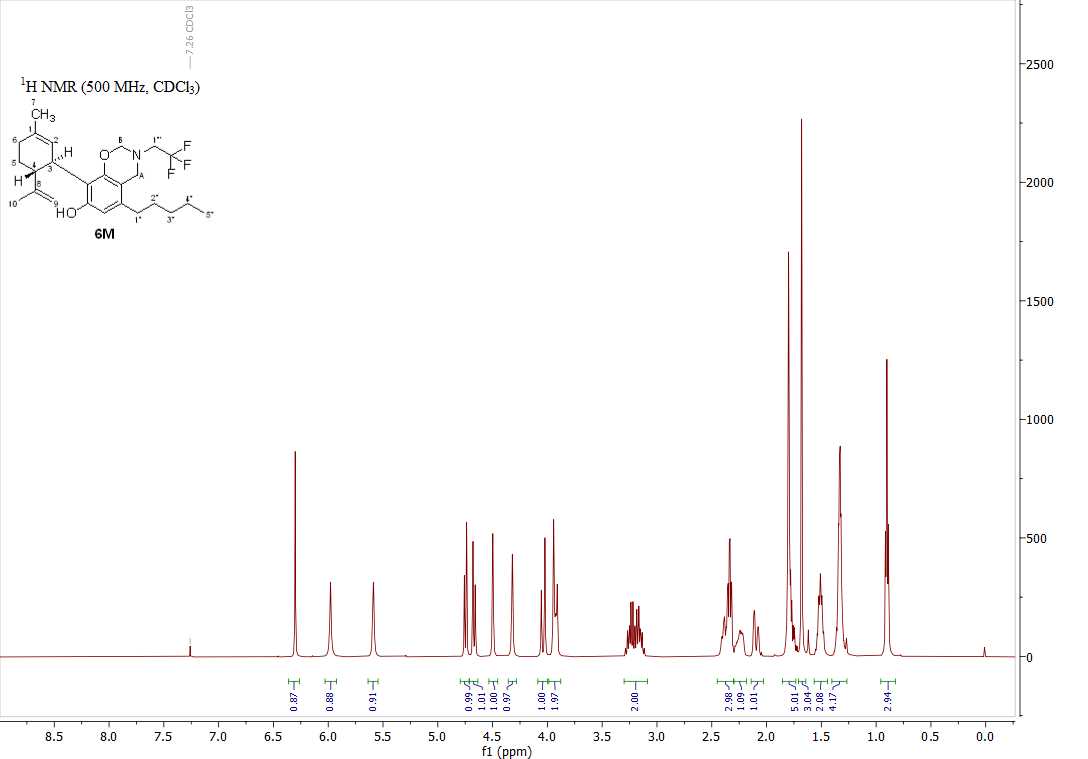


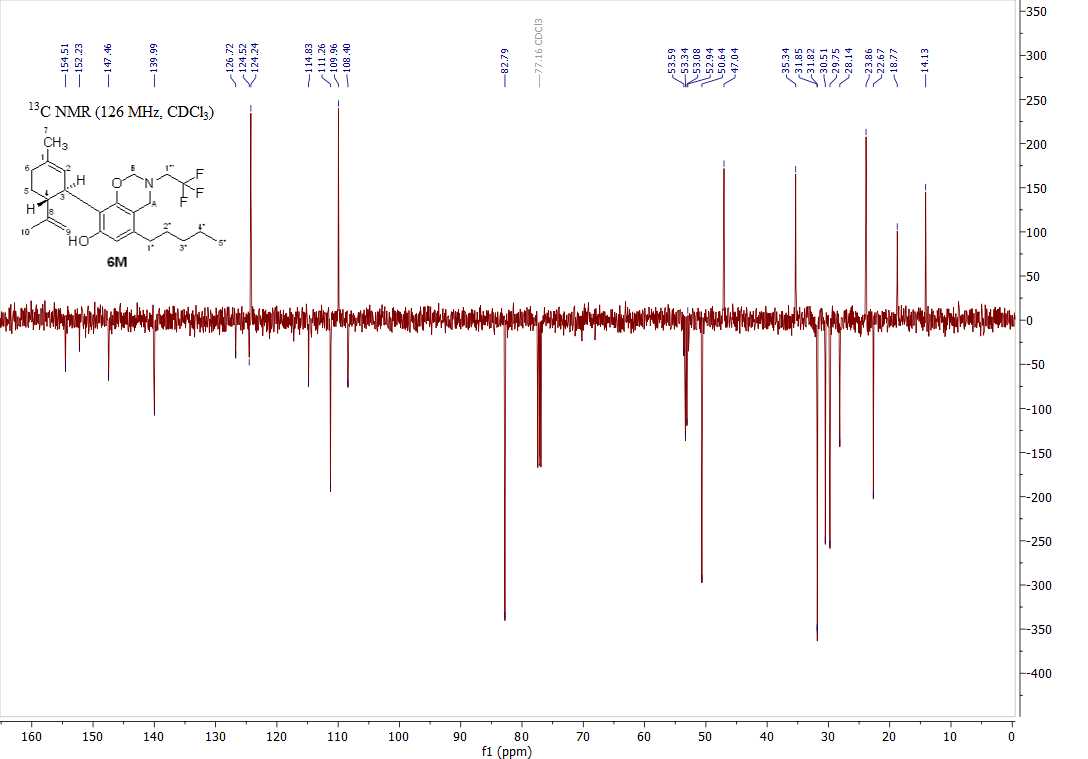


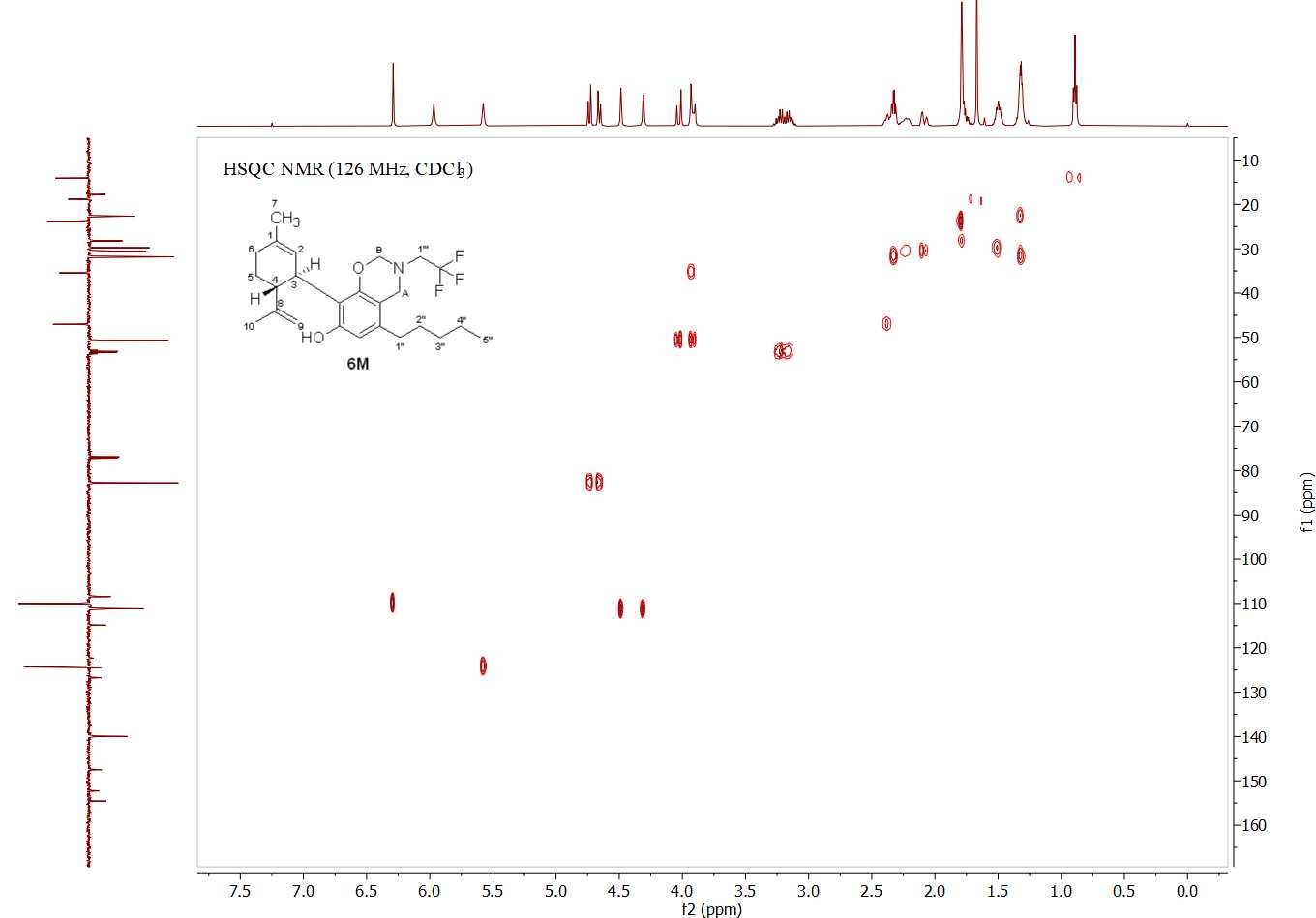


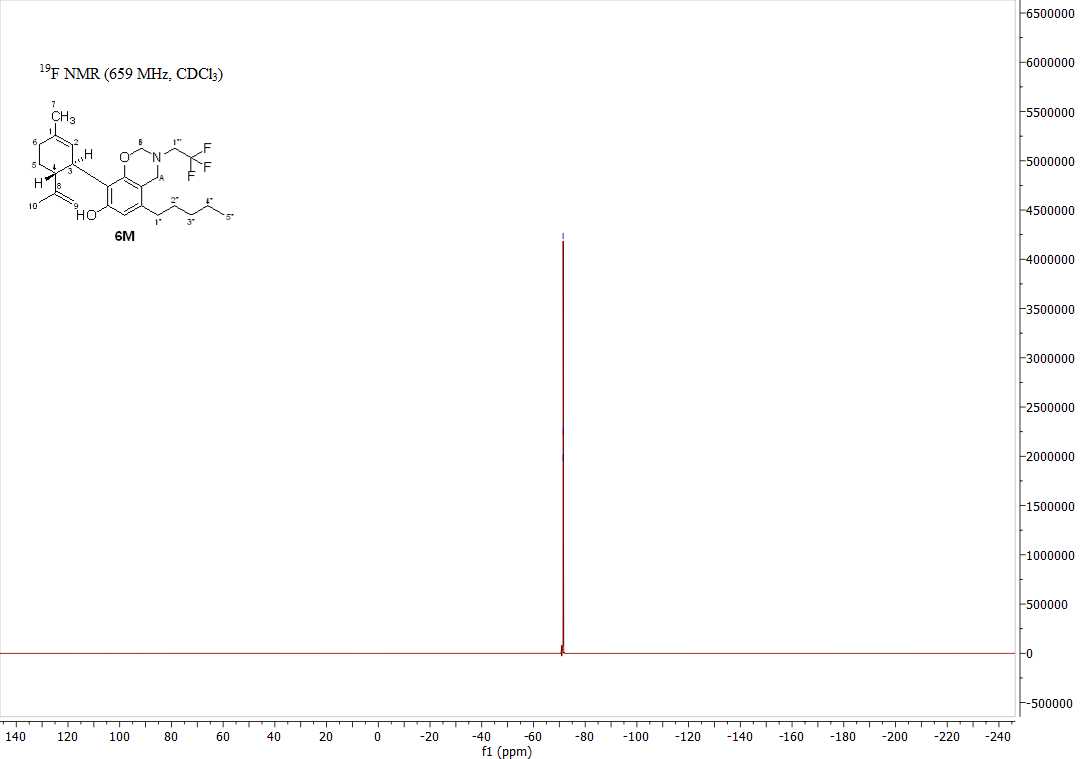


**NMR spectra of compound 7D**


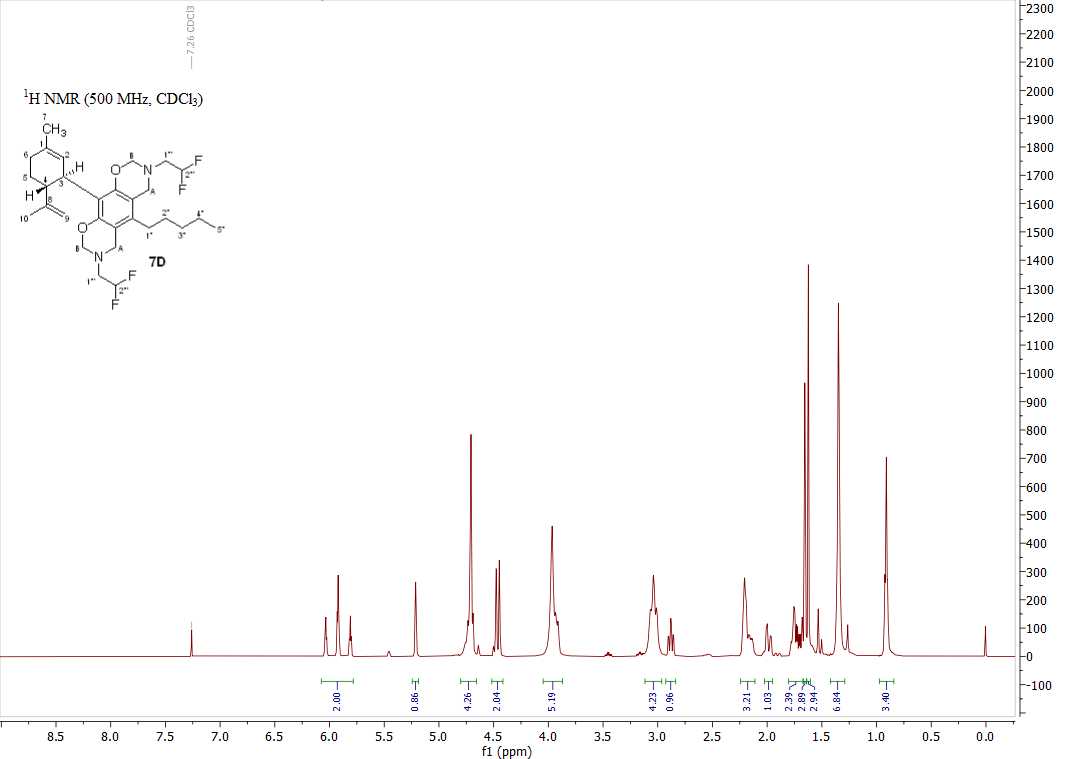


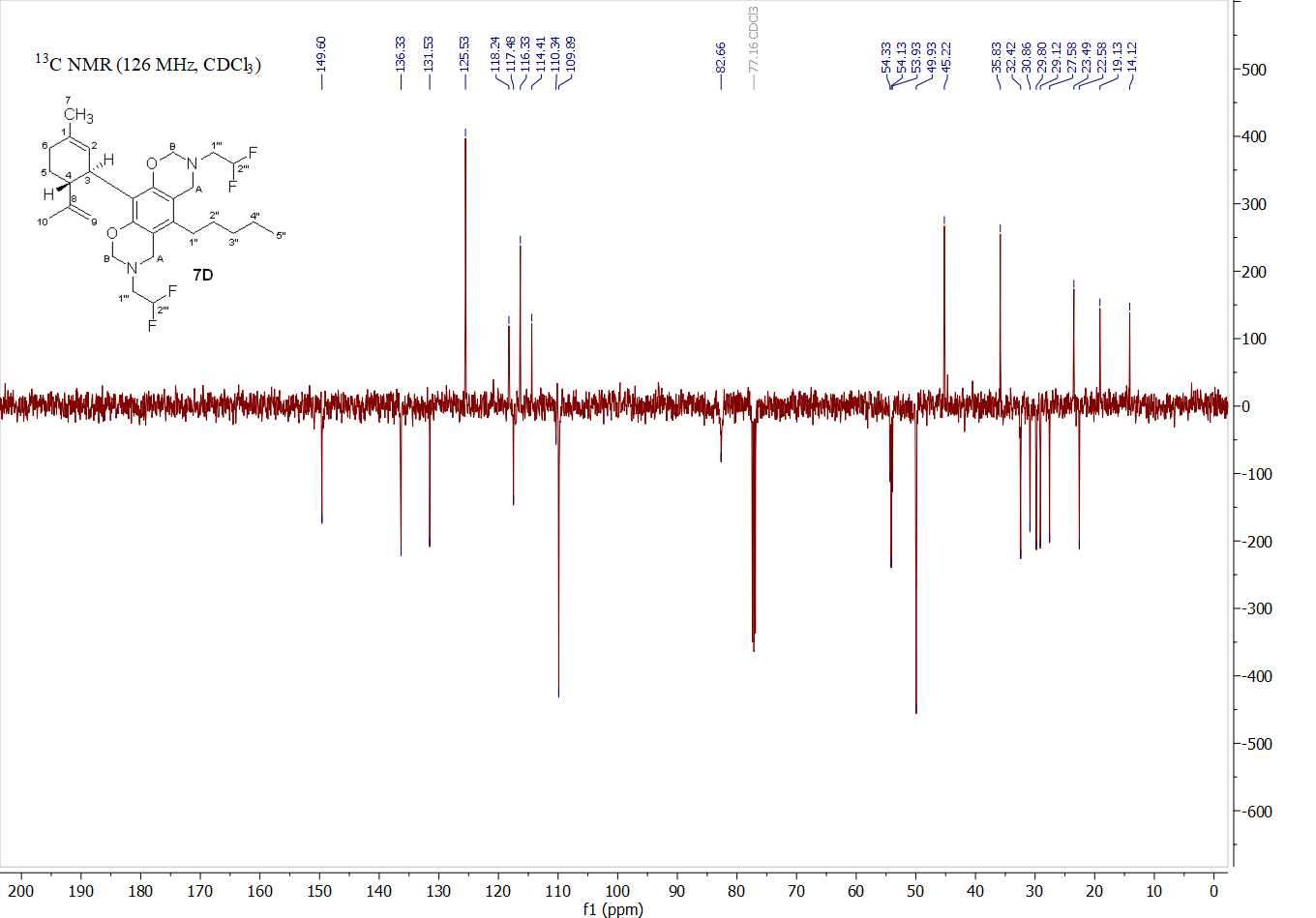


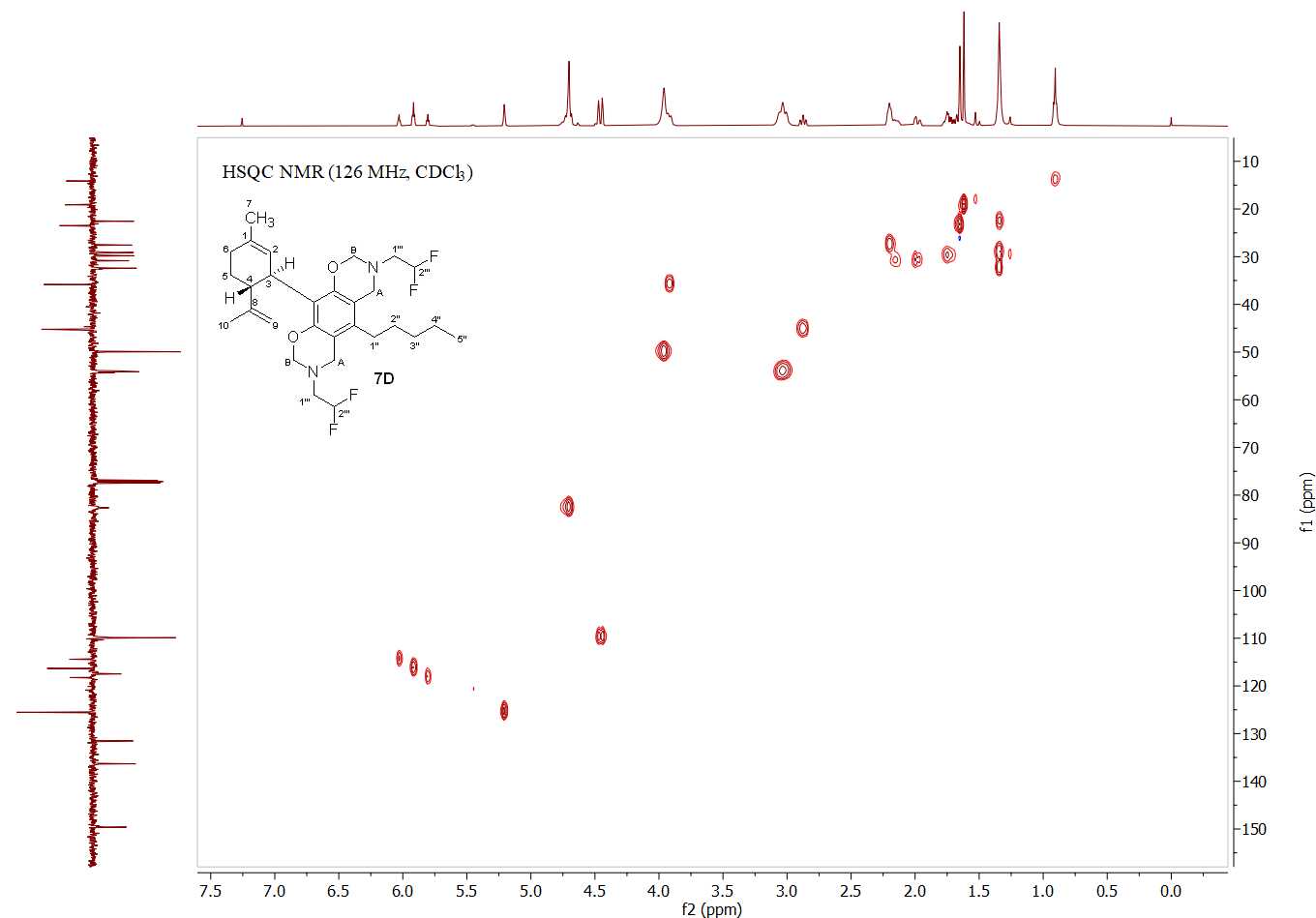


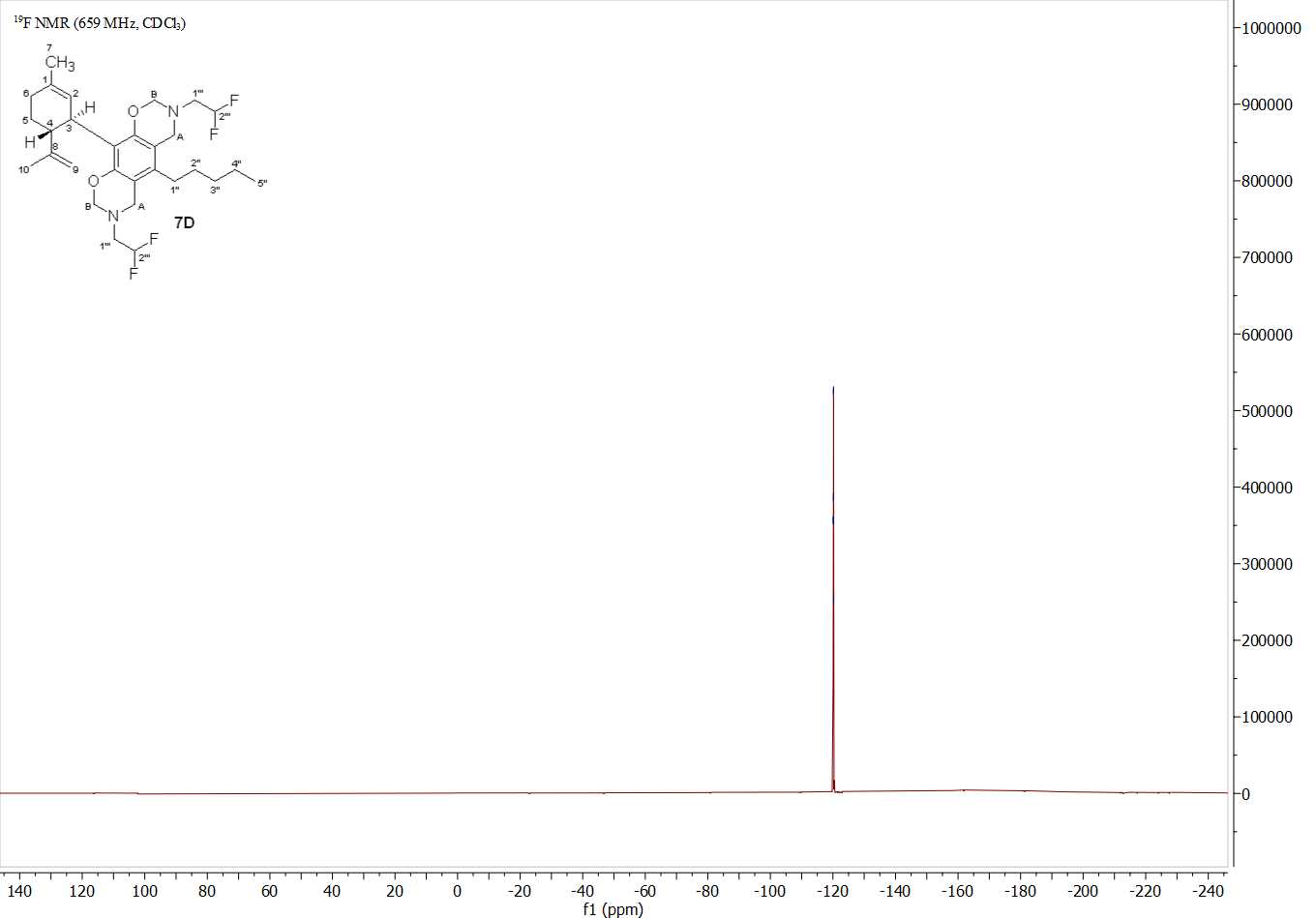


**NMR spectra of compound 7M**


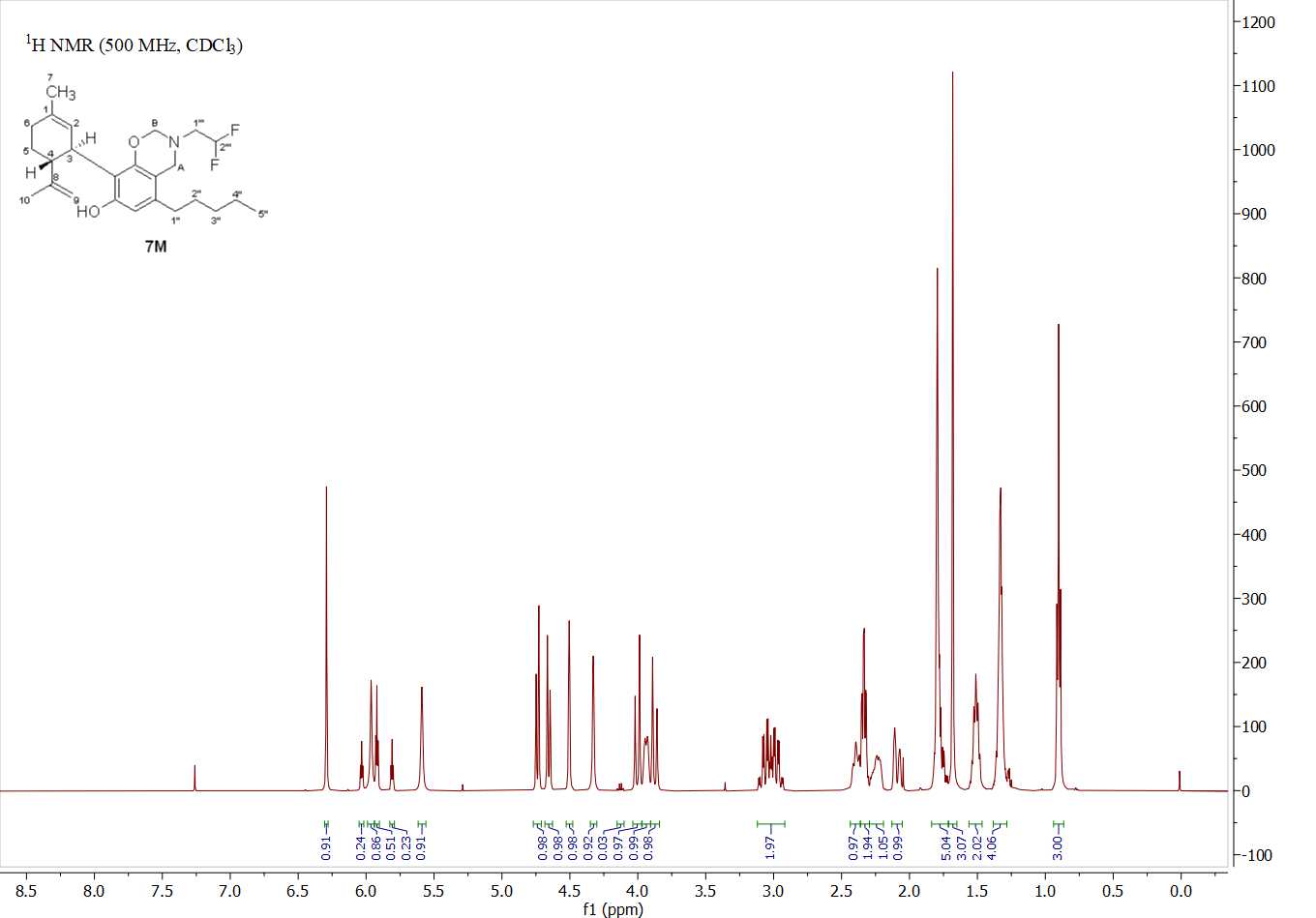


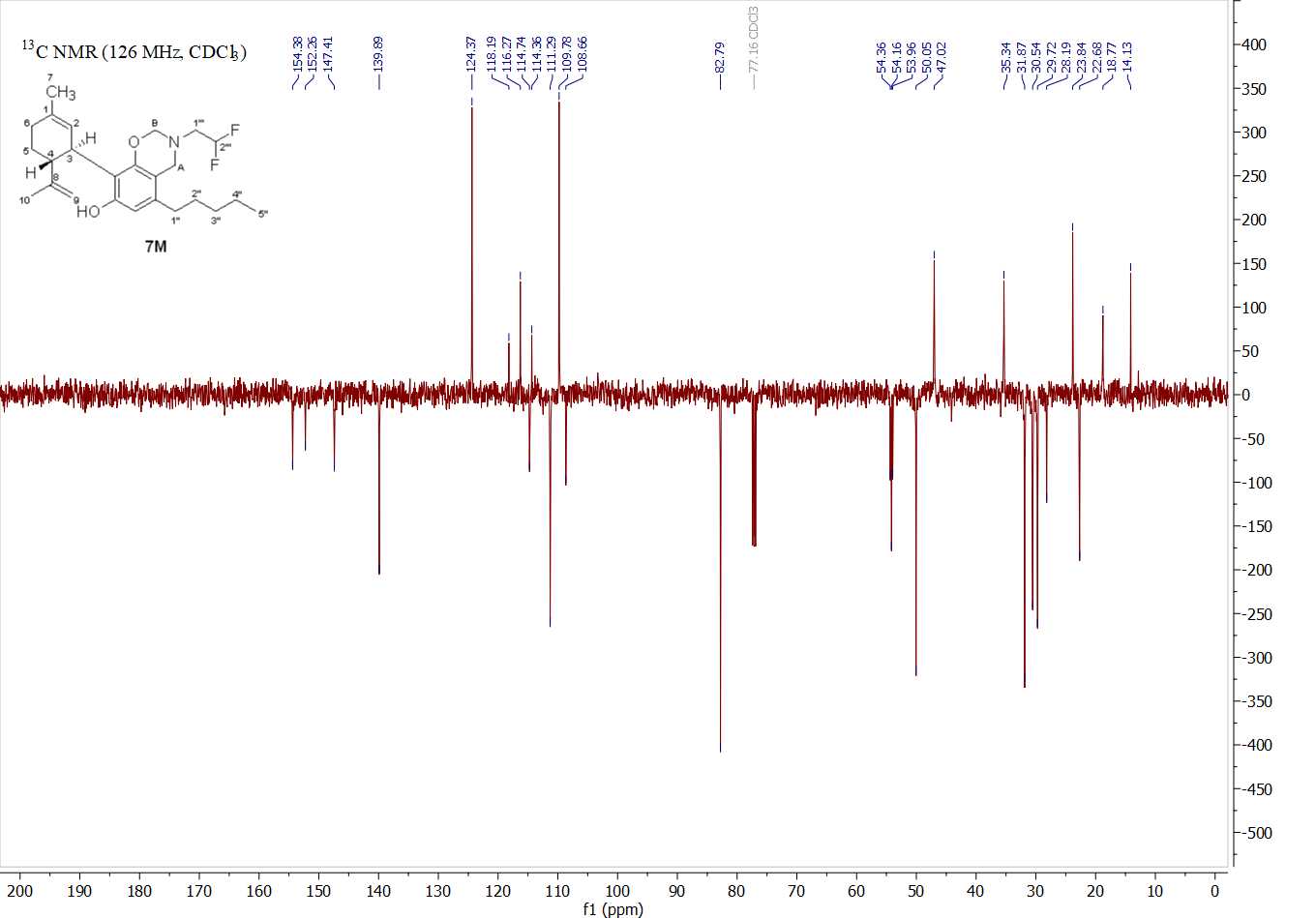


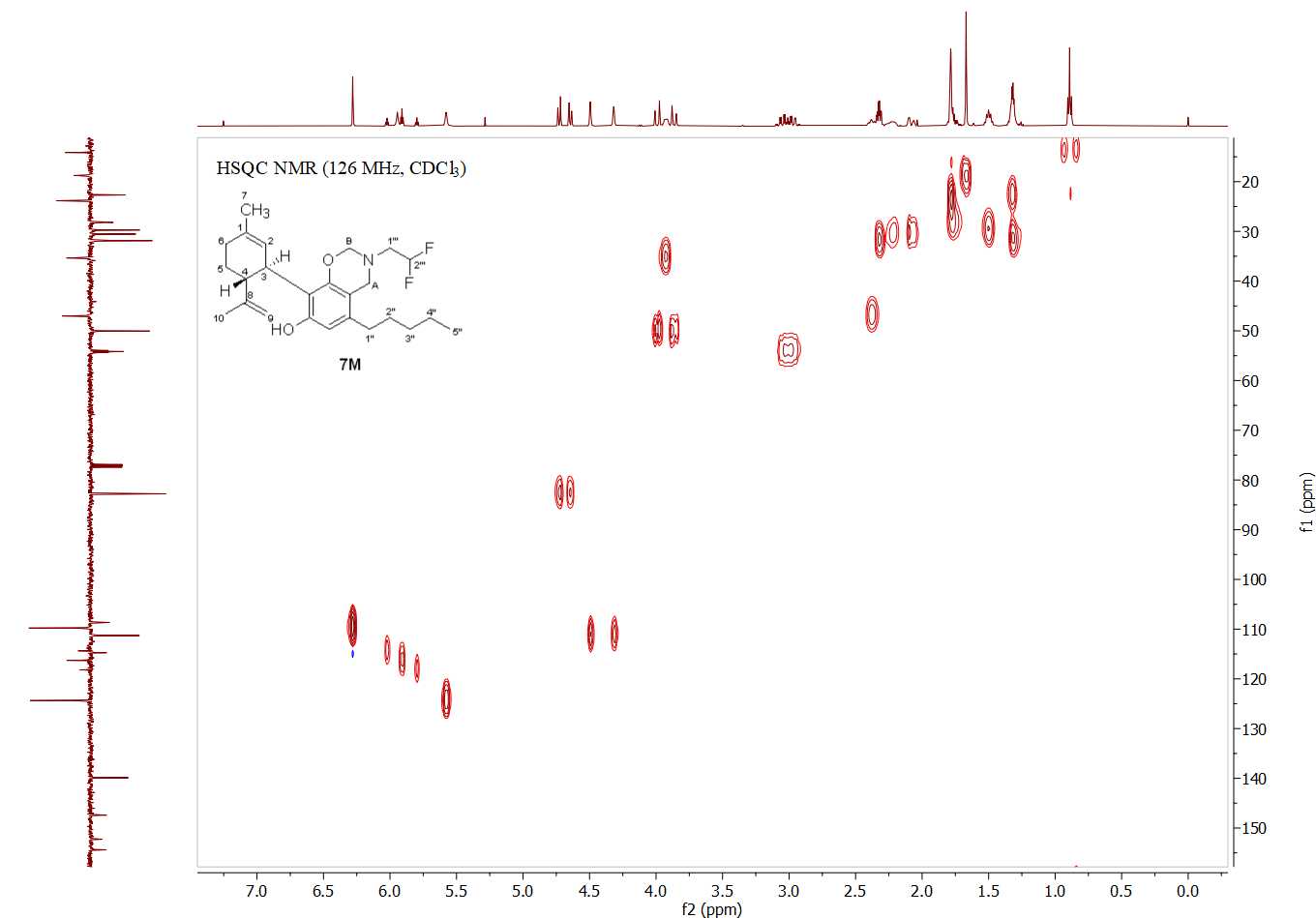


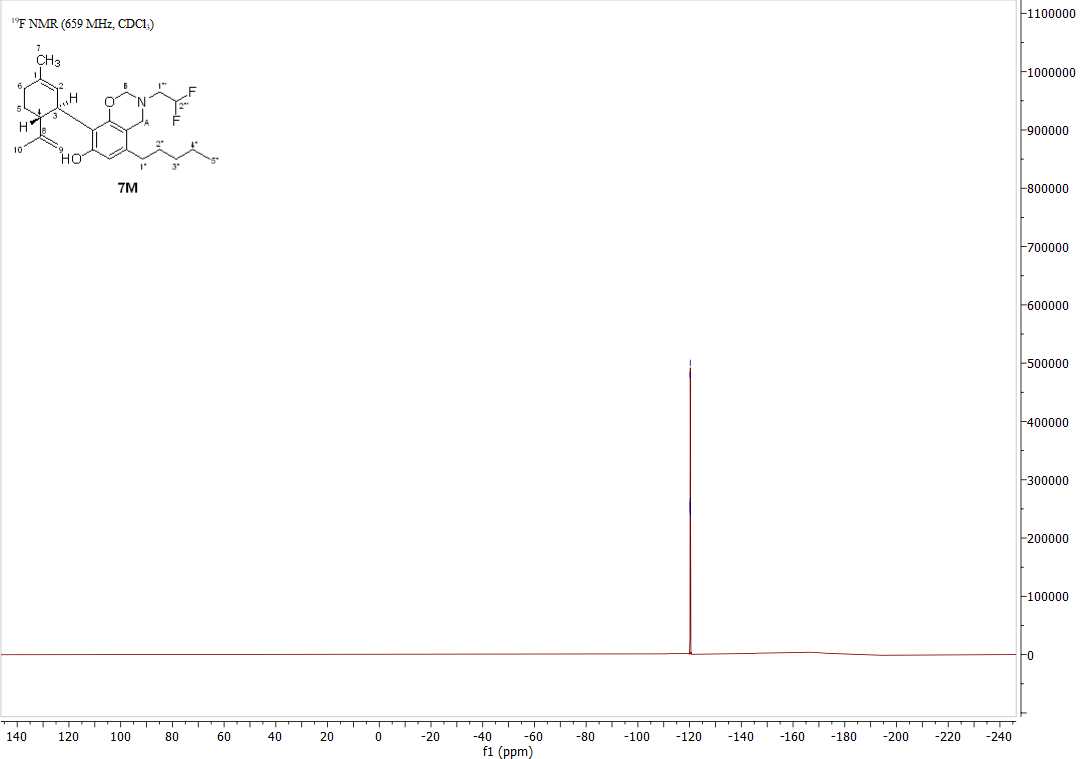


**NMR spectra of compound 8M**


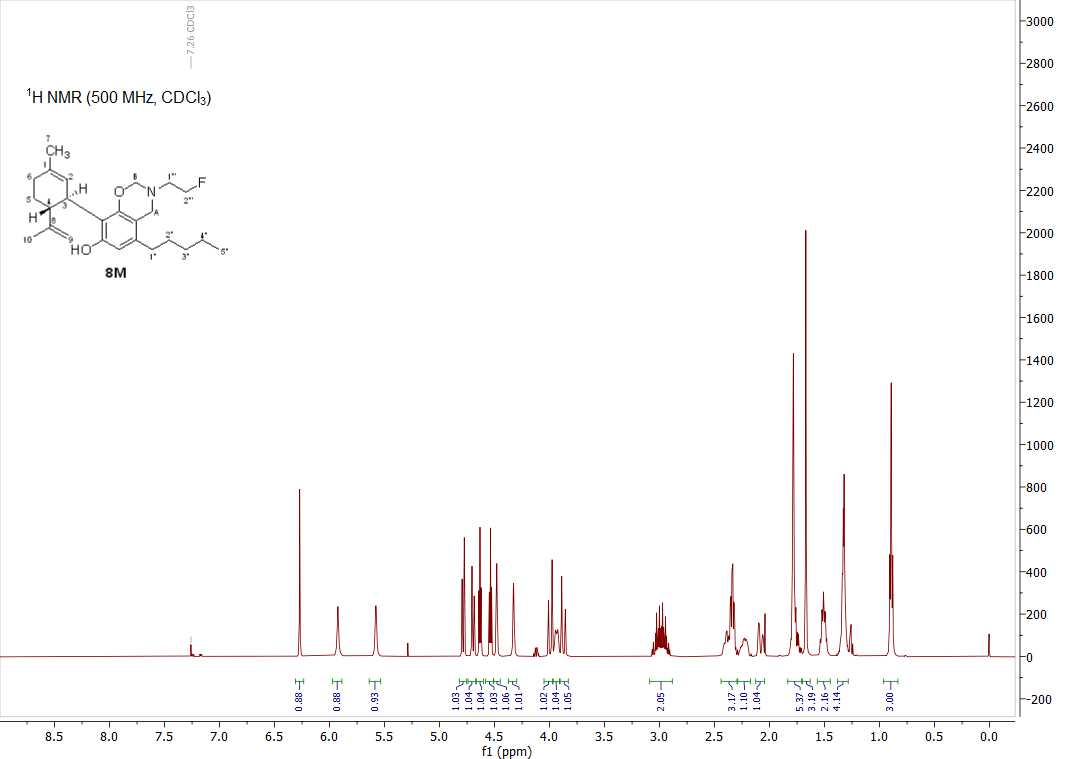


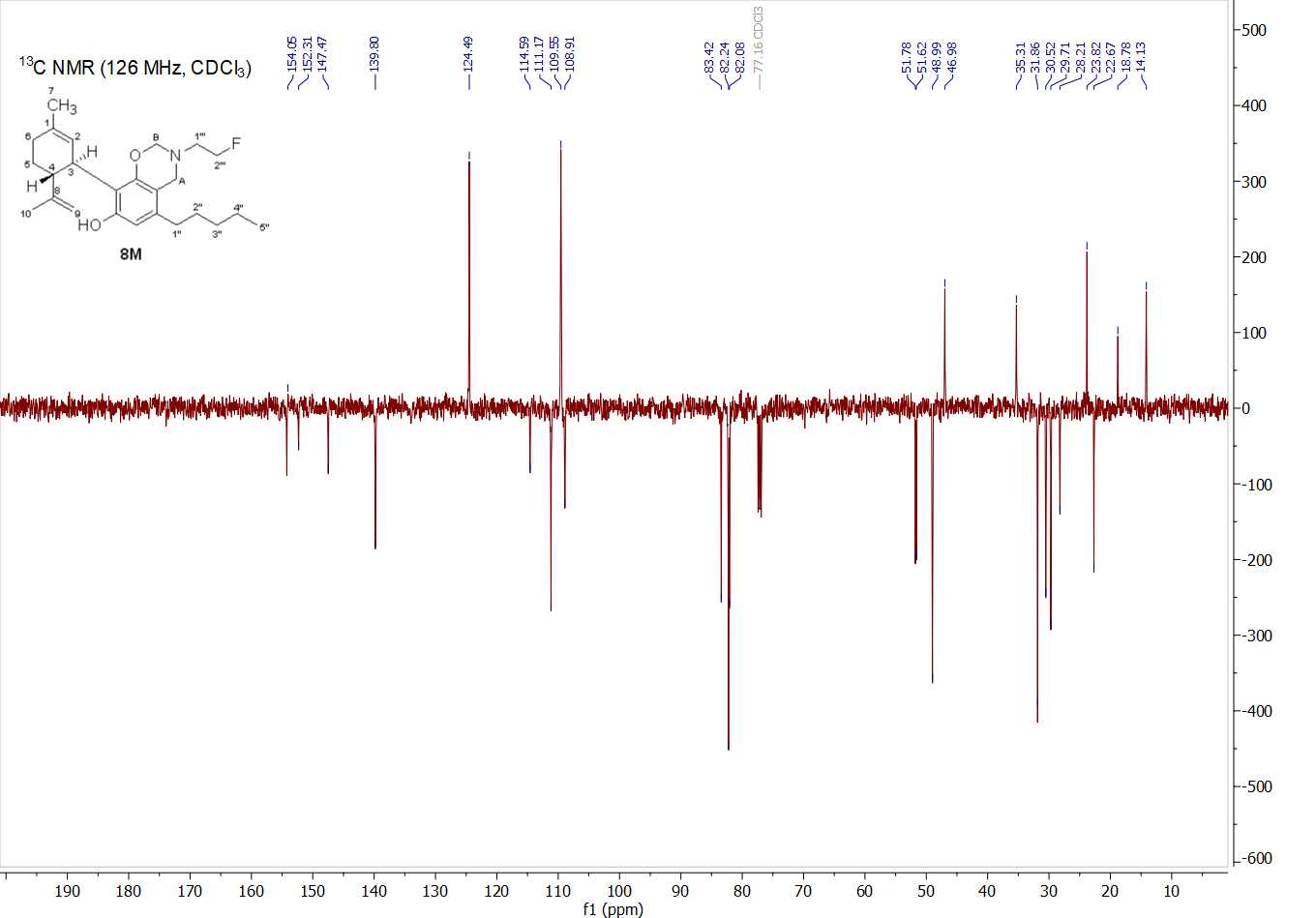


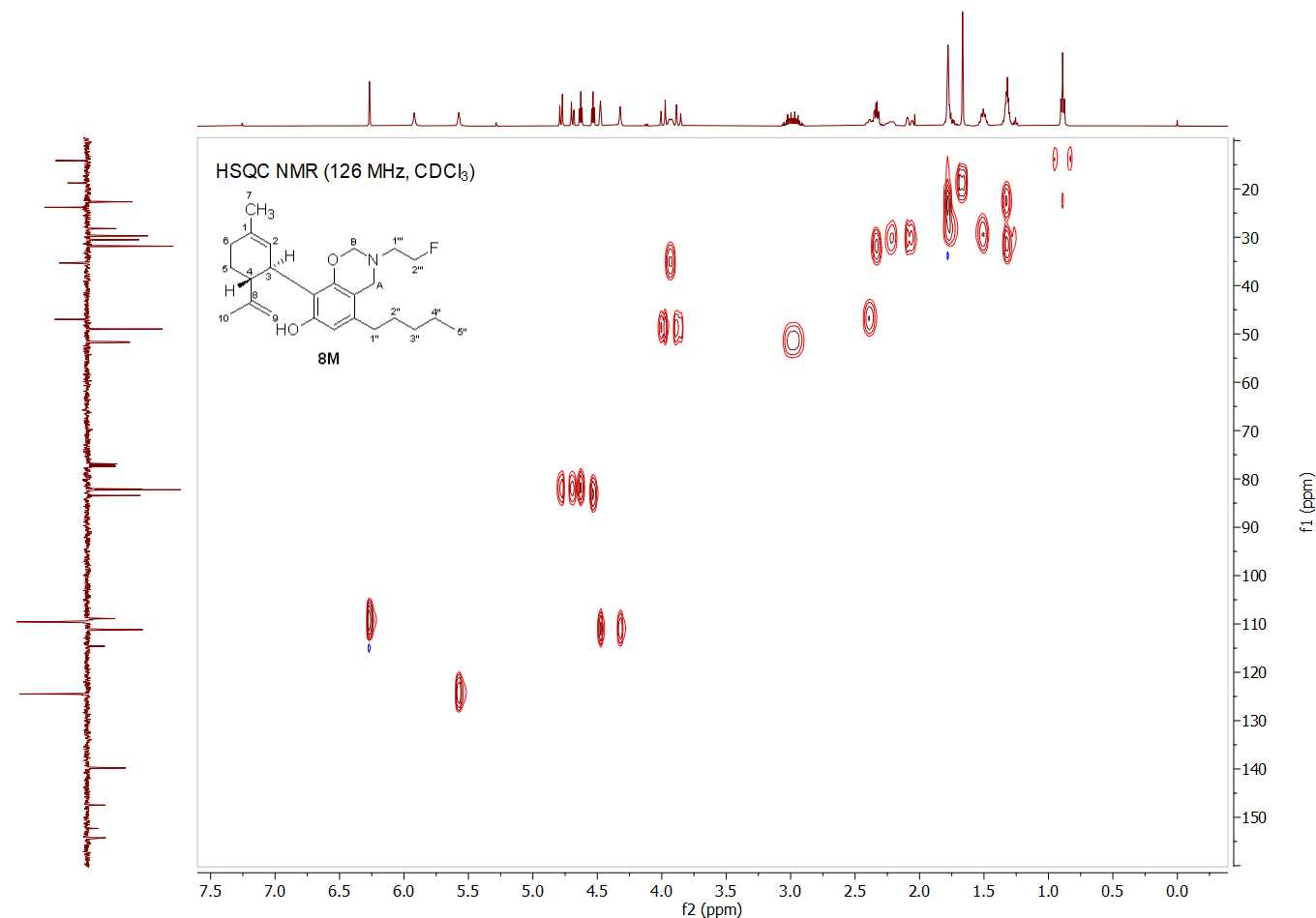


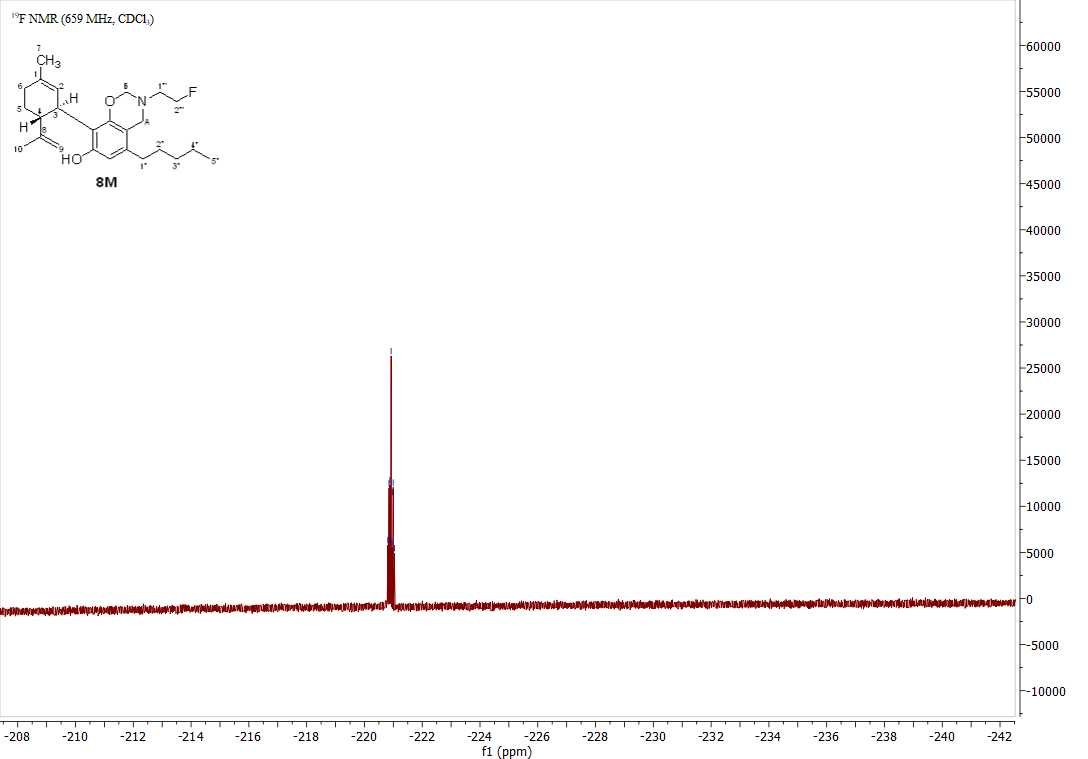


**NMR spectra of compound 9M**


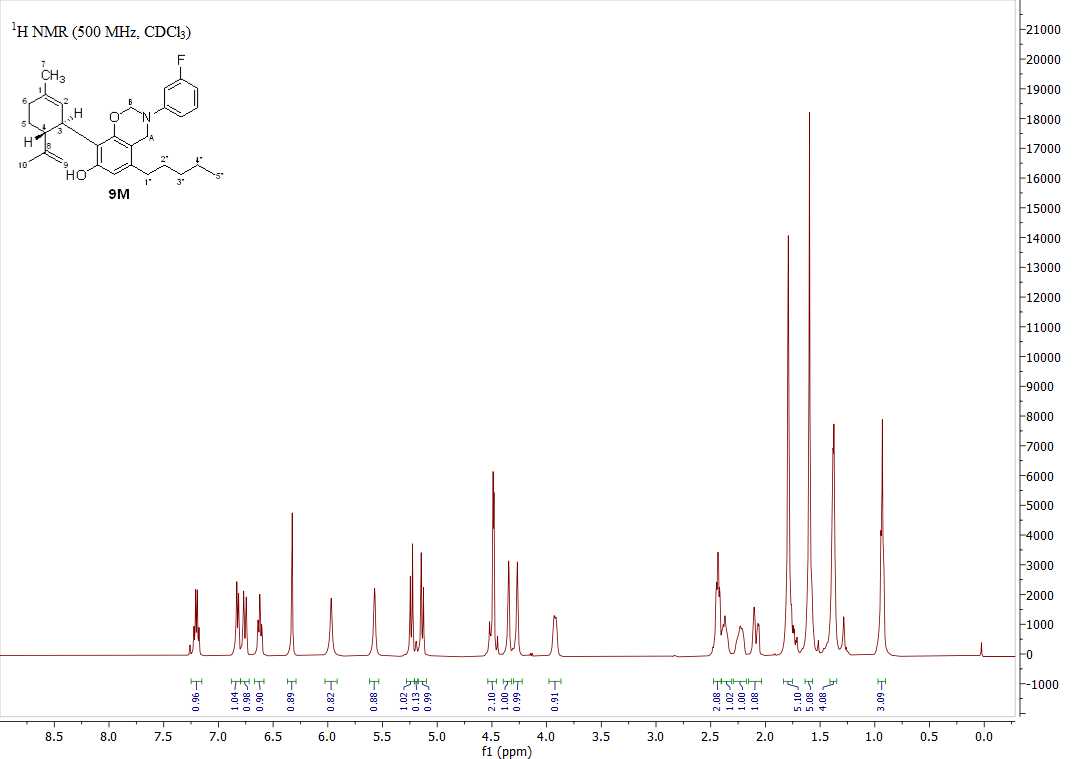


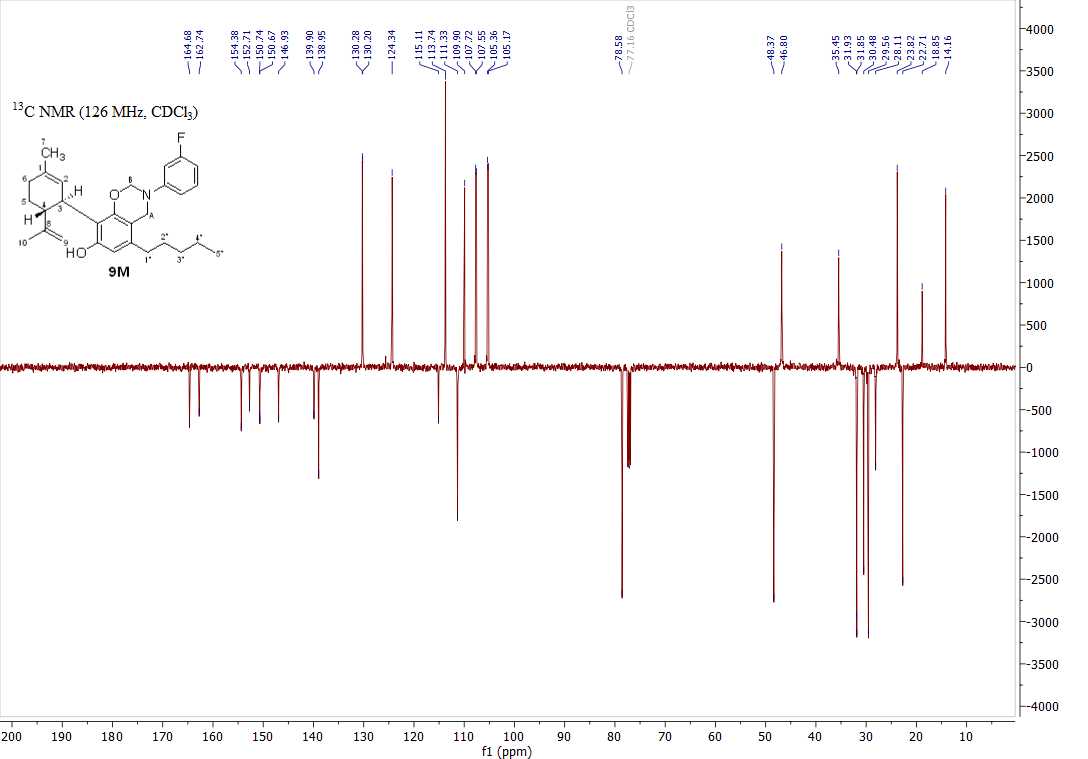


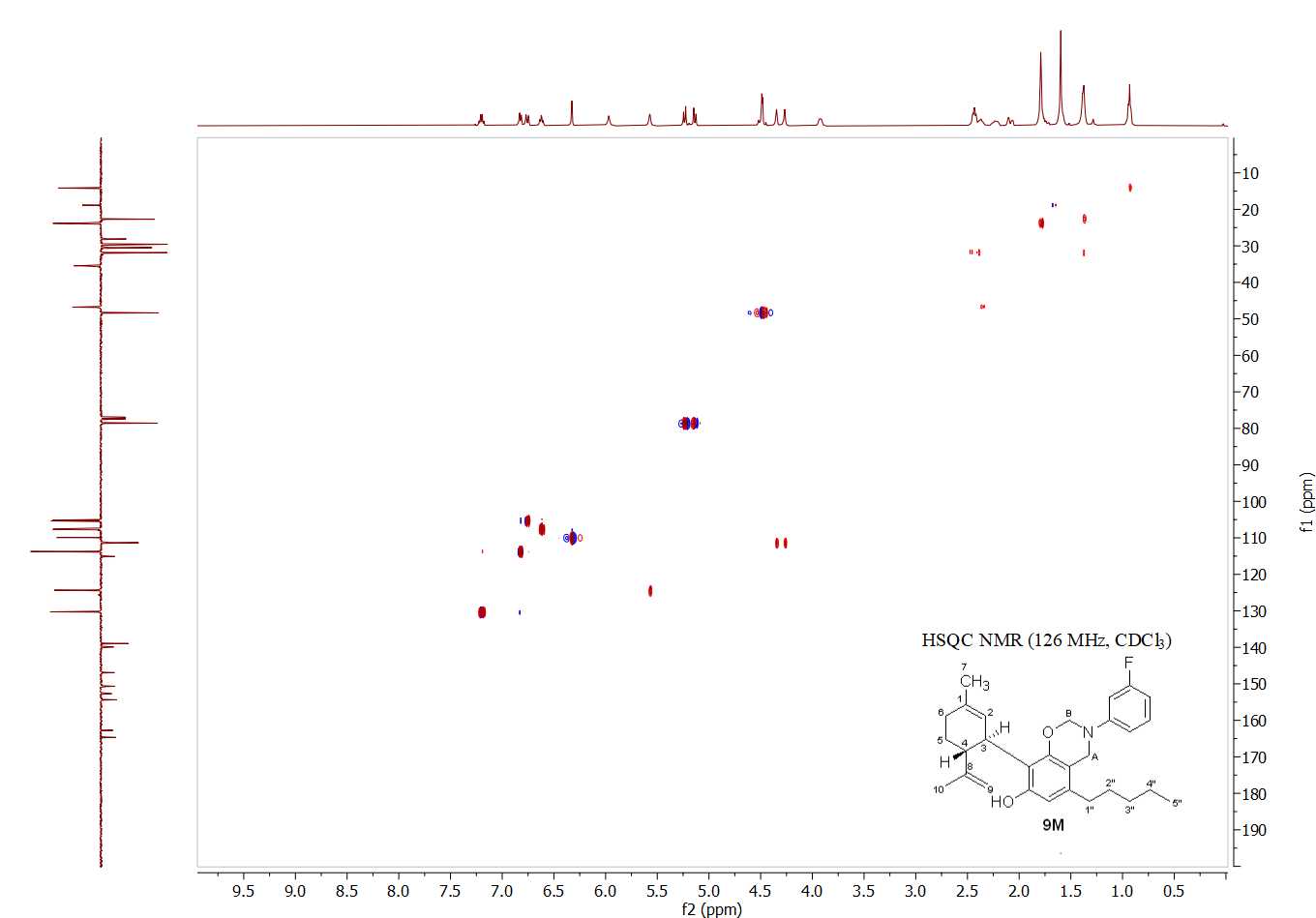


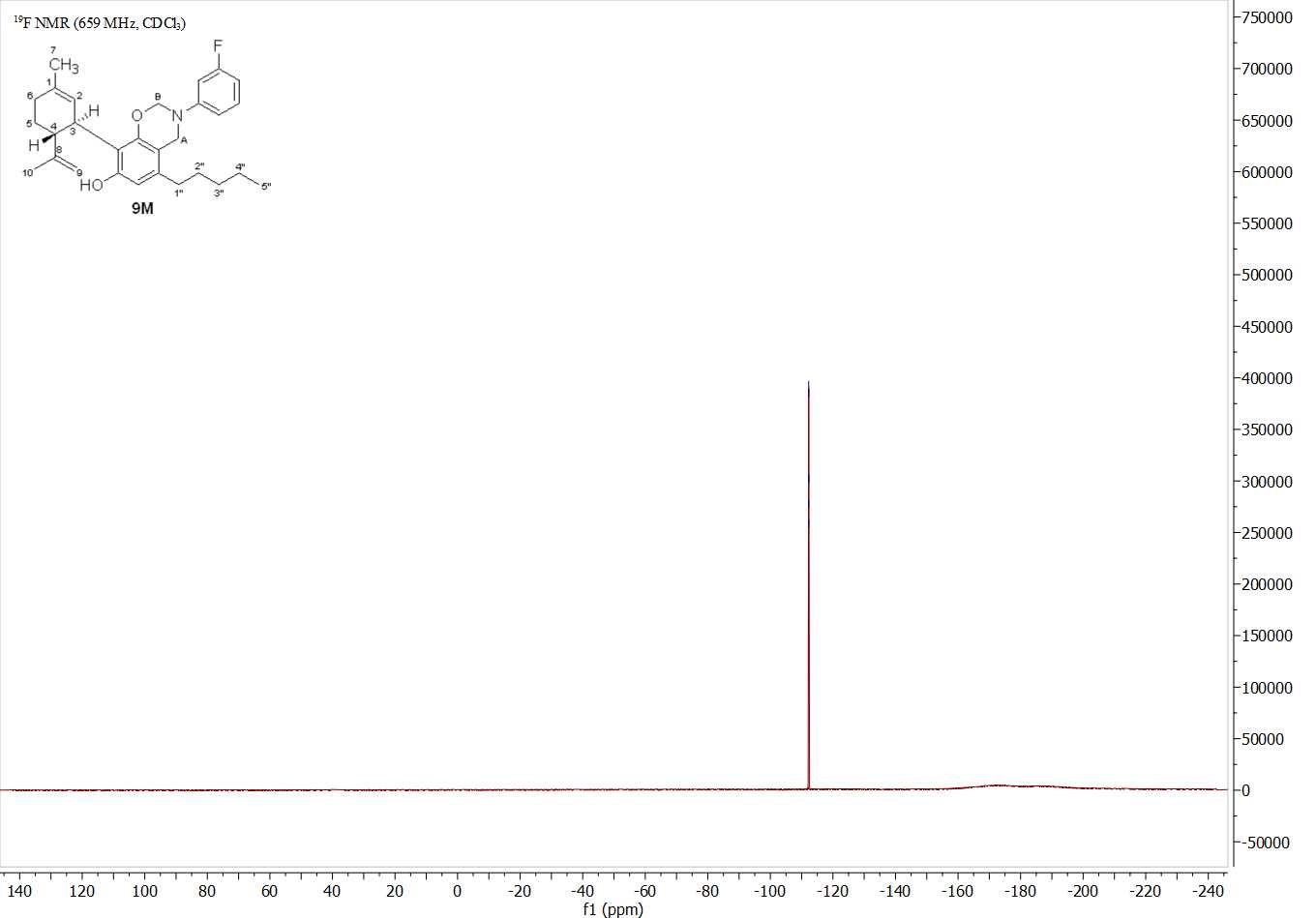


**NMR spectra of compound 10M**


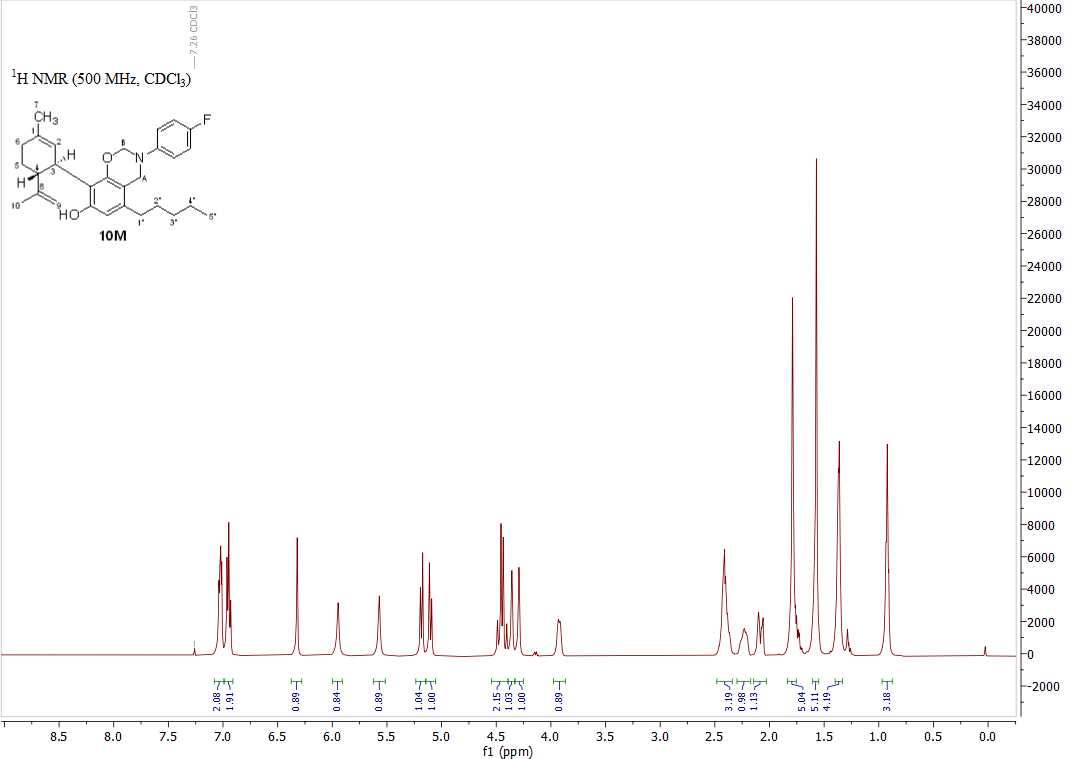


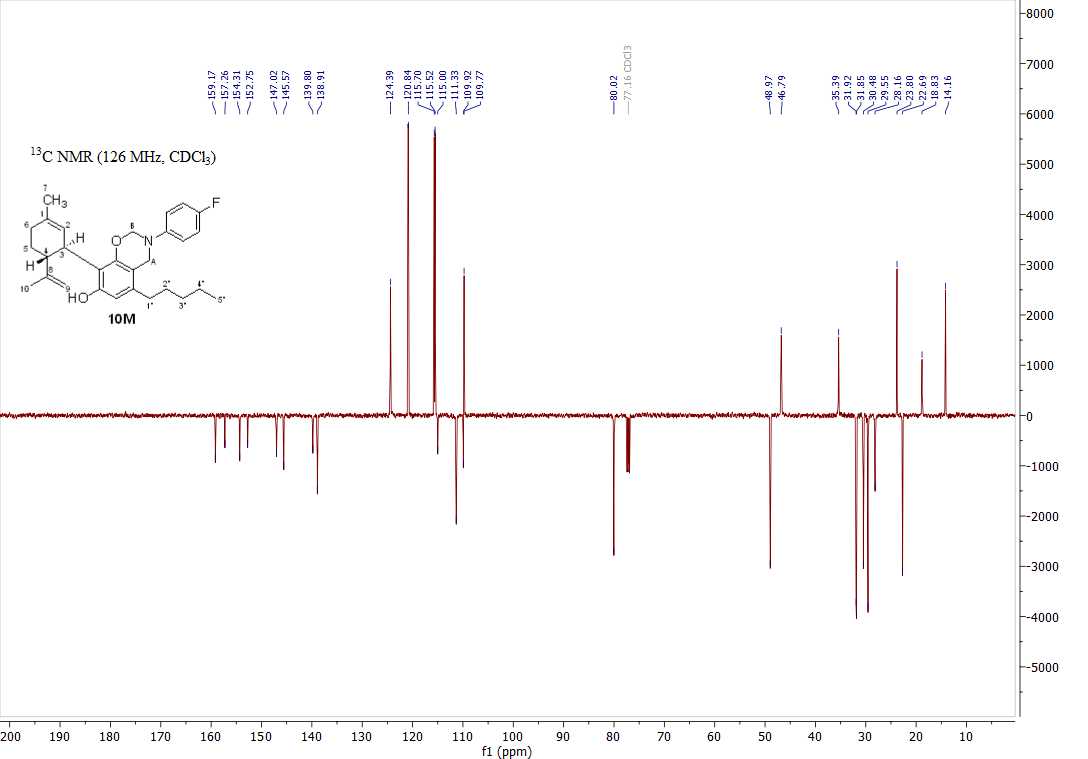


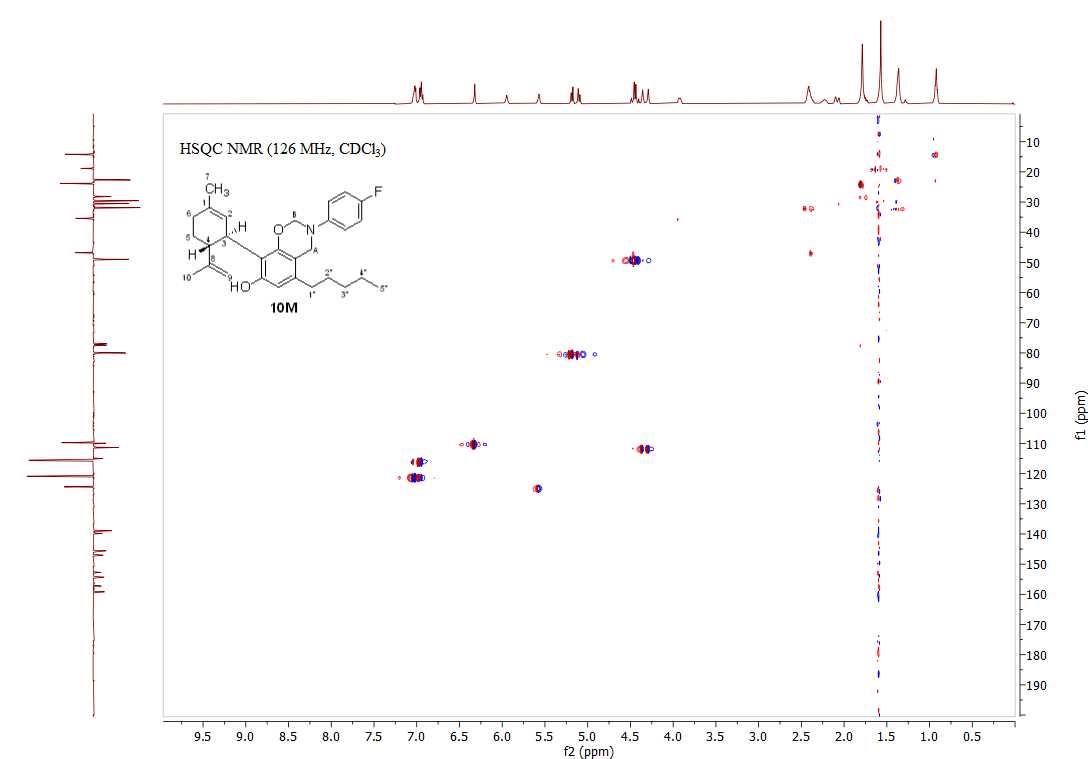


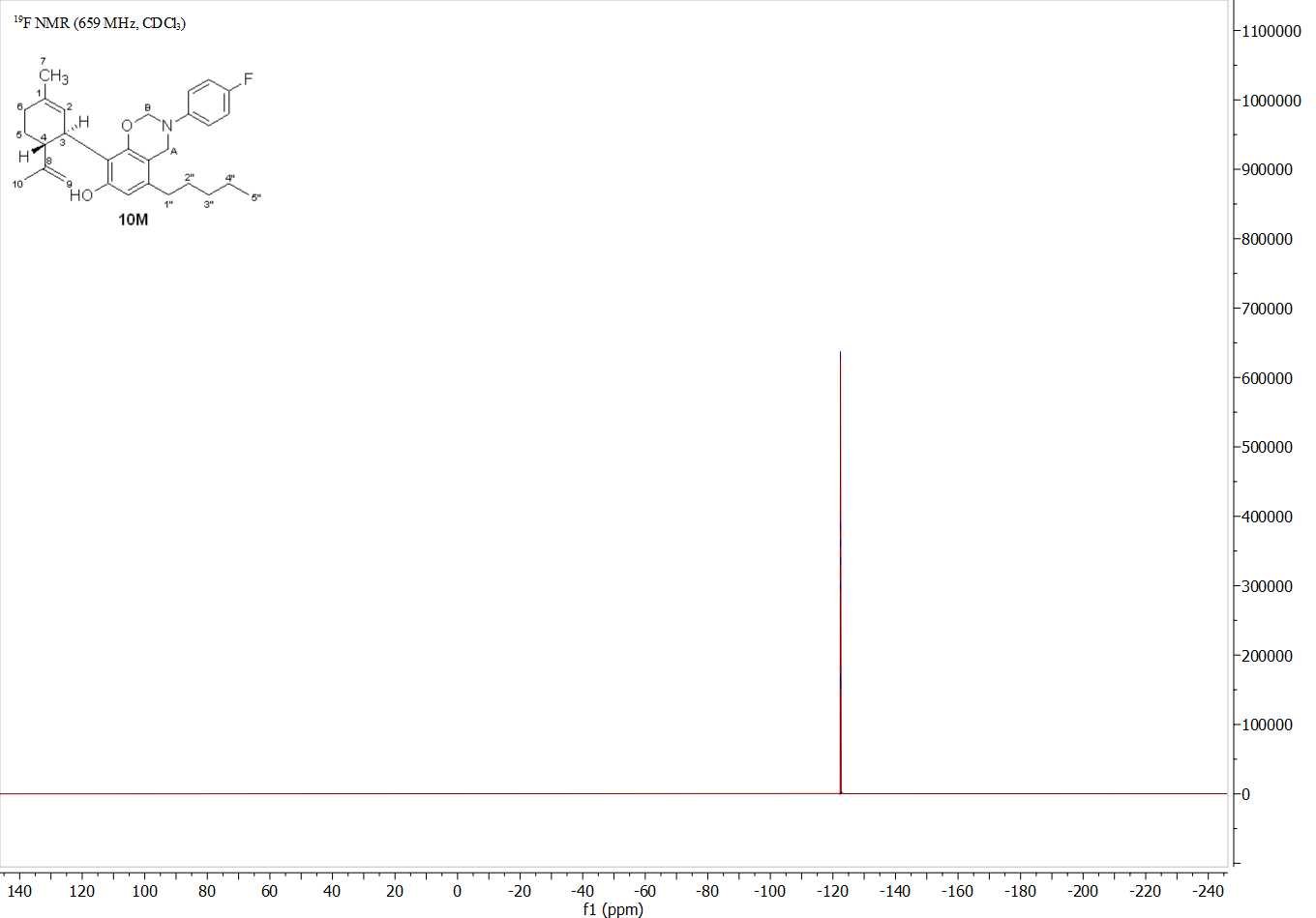


**NMR spectra of compound 11D**


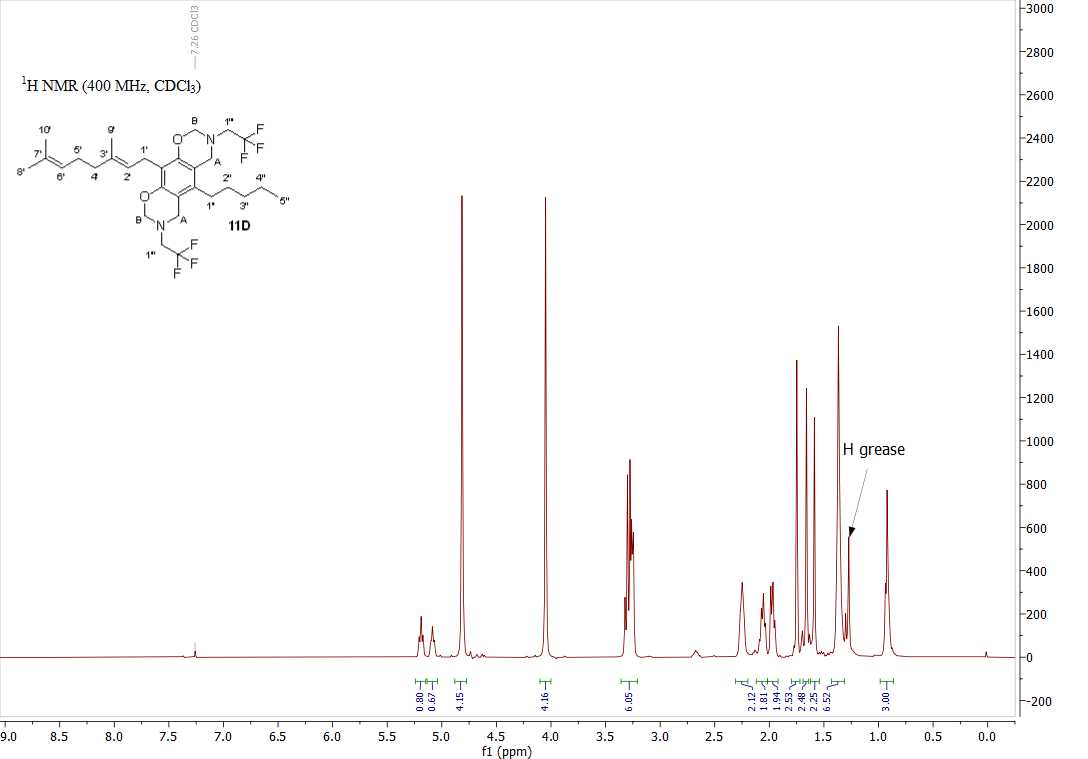


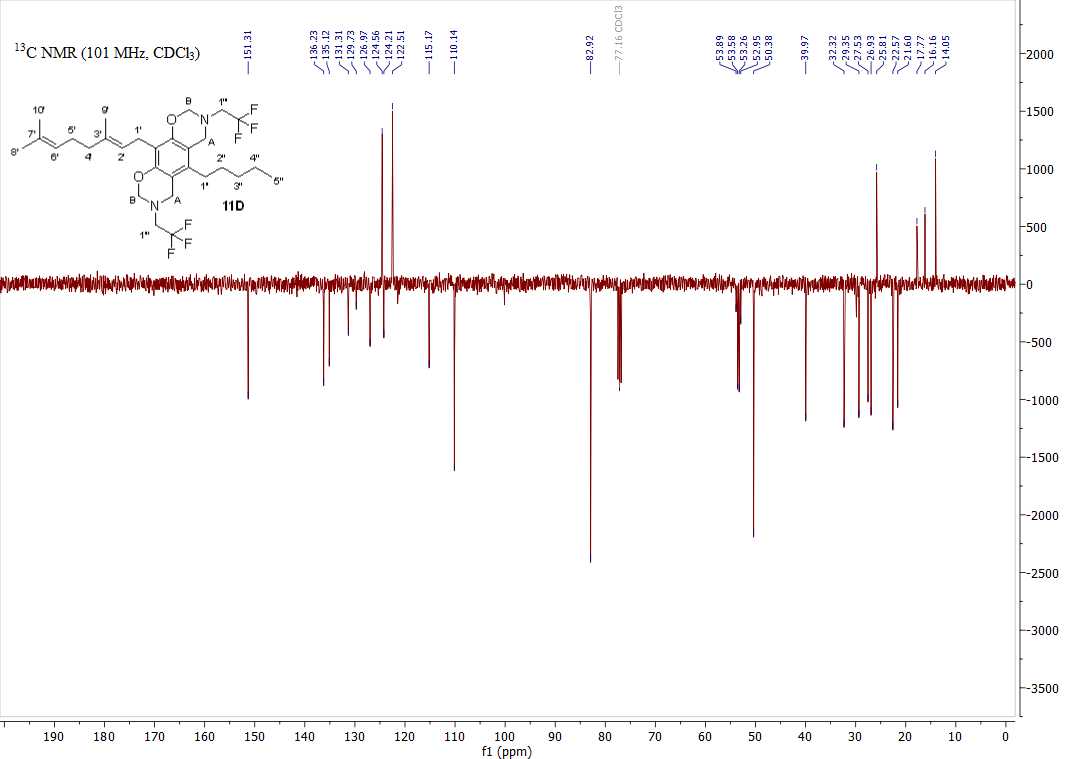


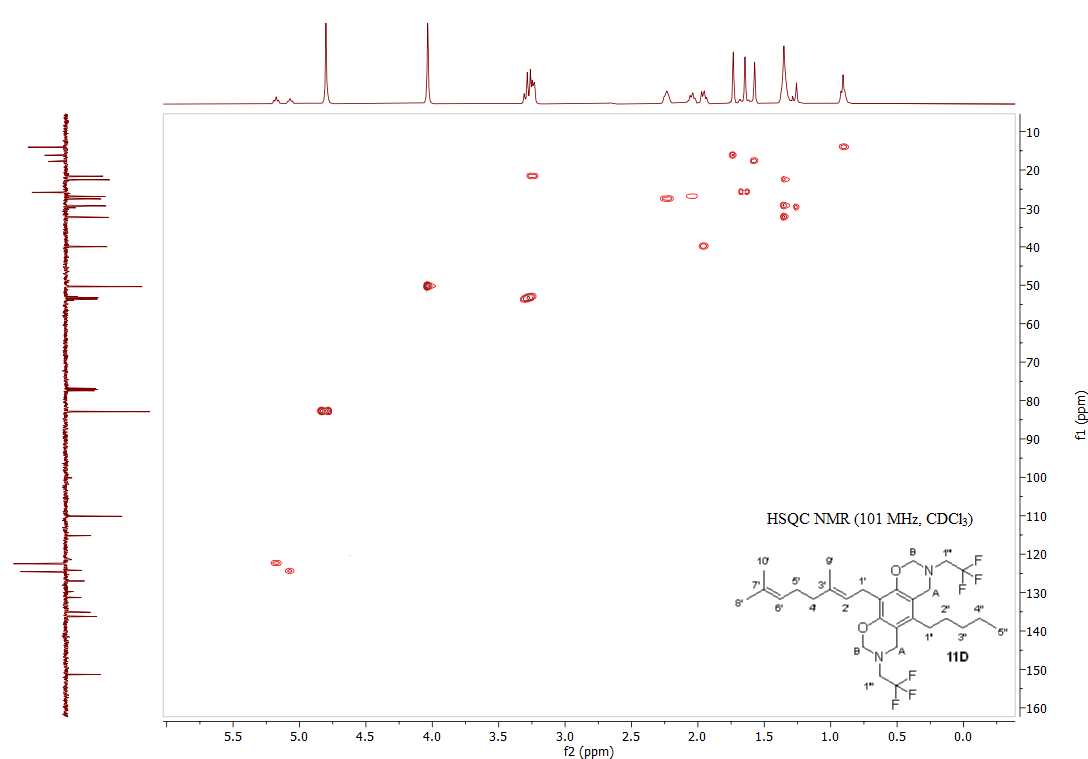


**
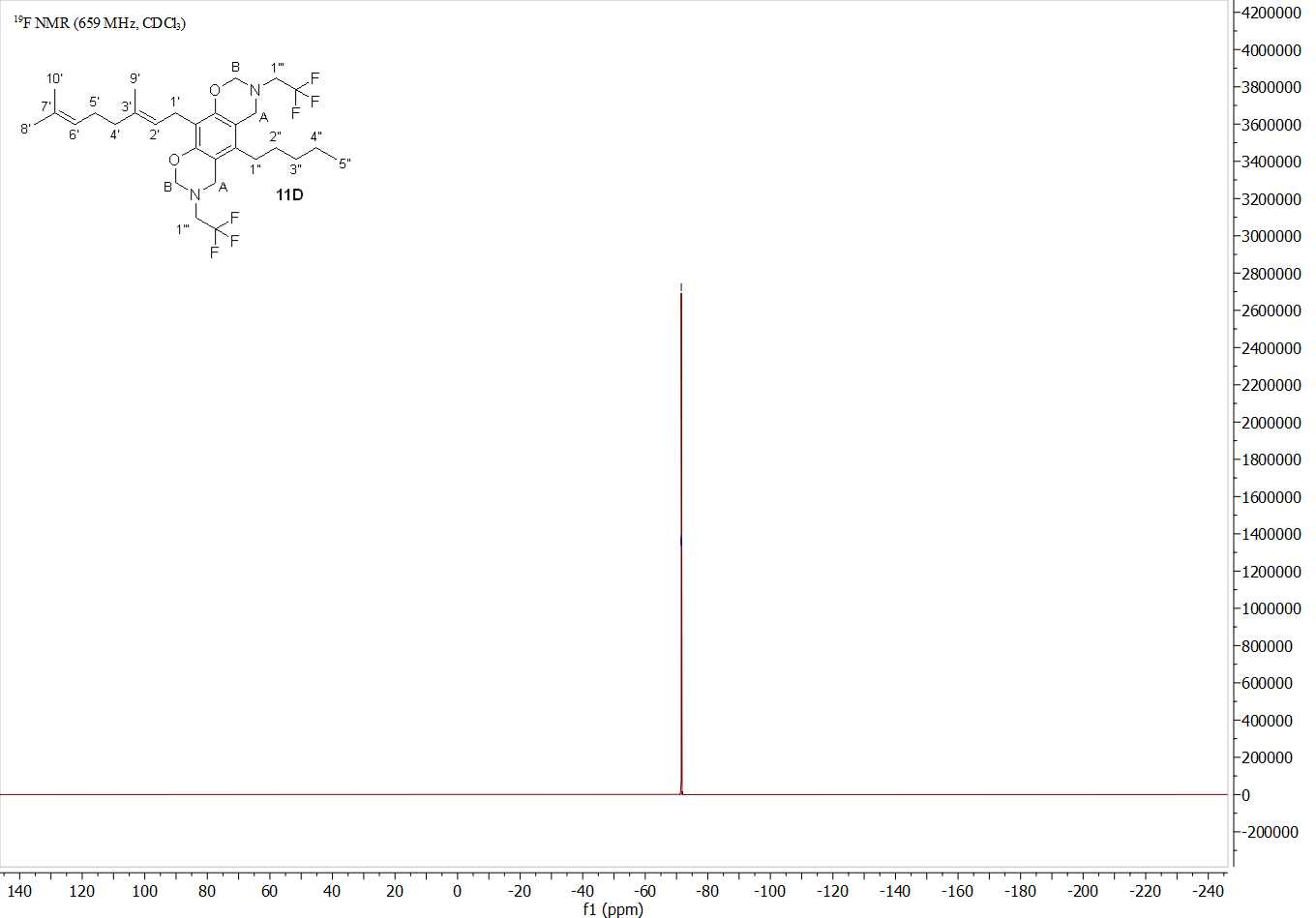
**

**NMR spectra of compound 11M**


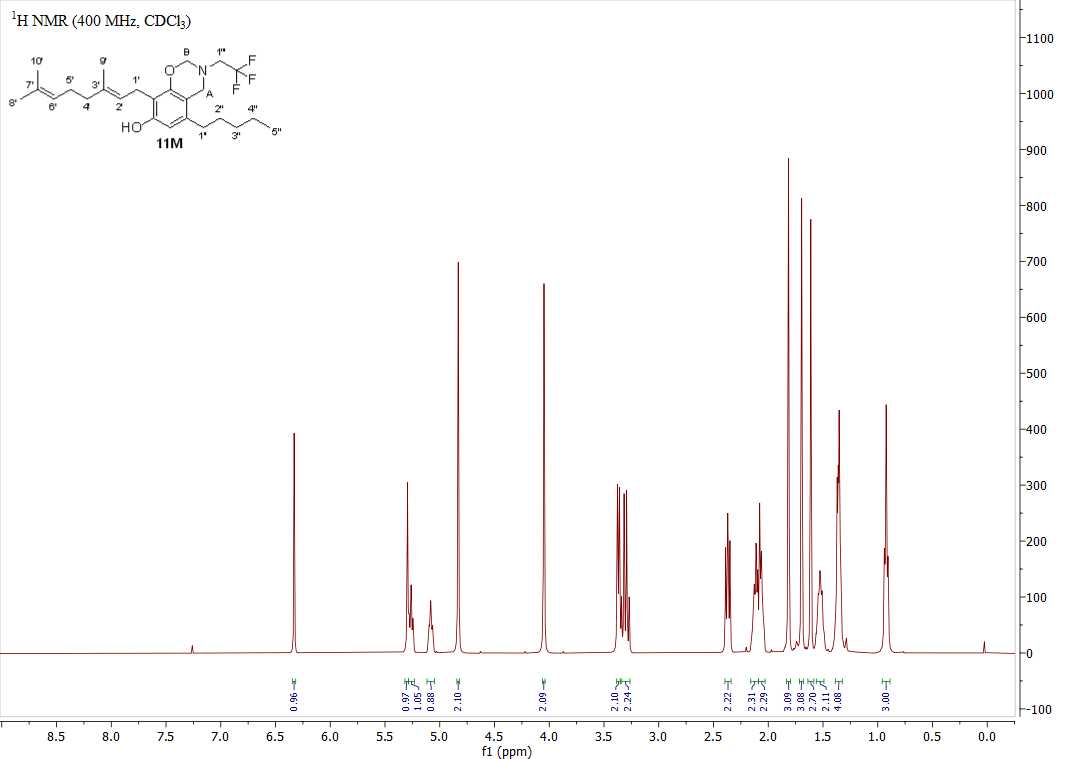


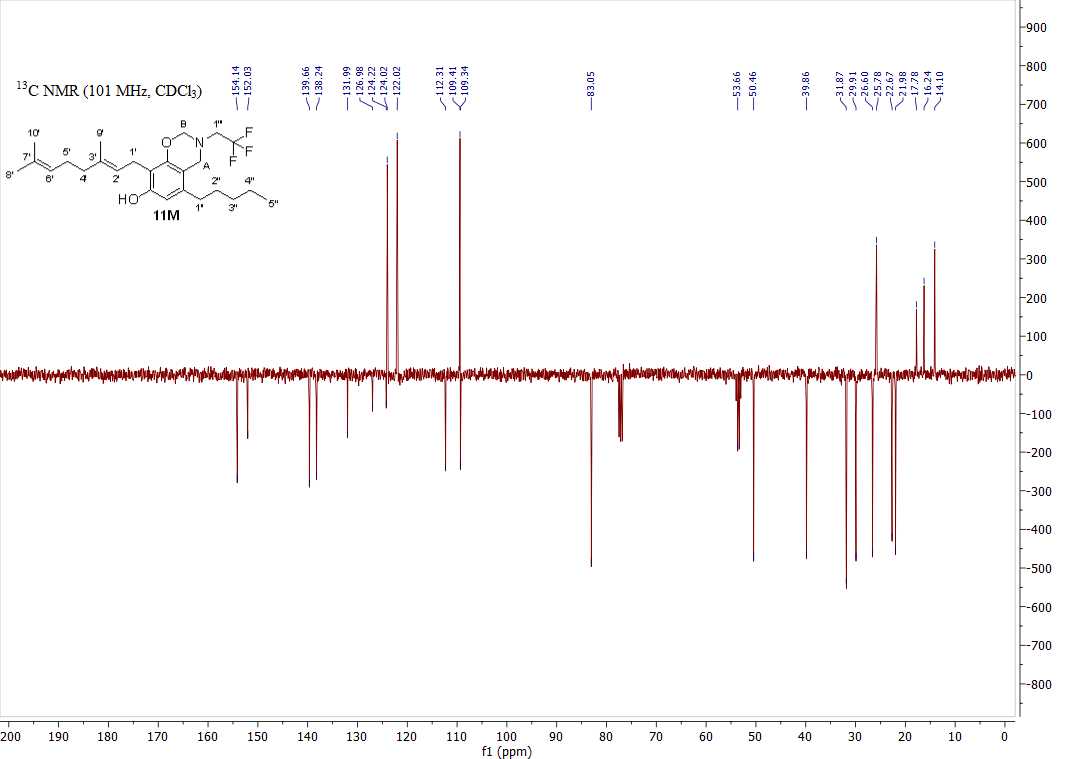


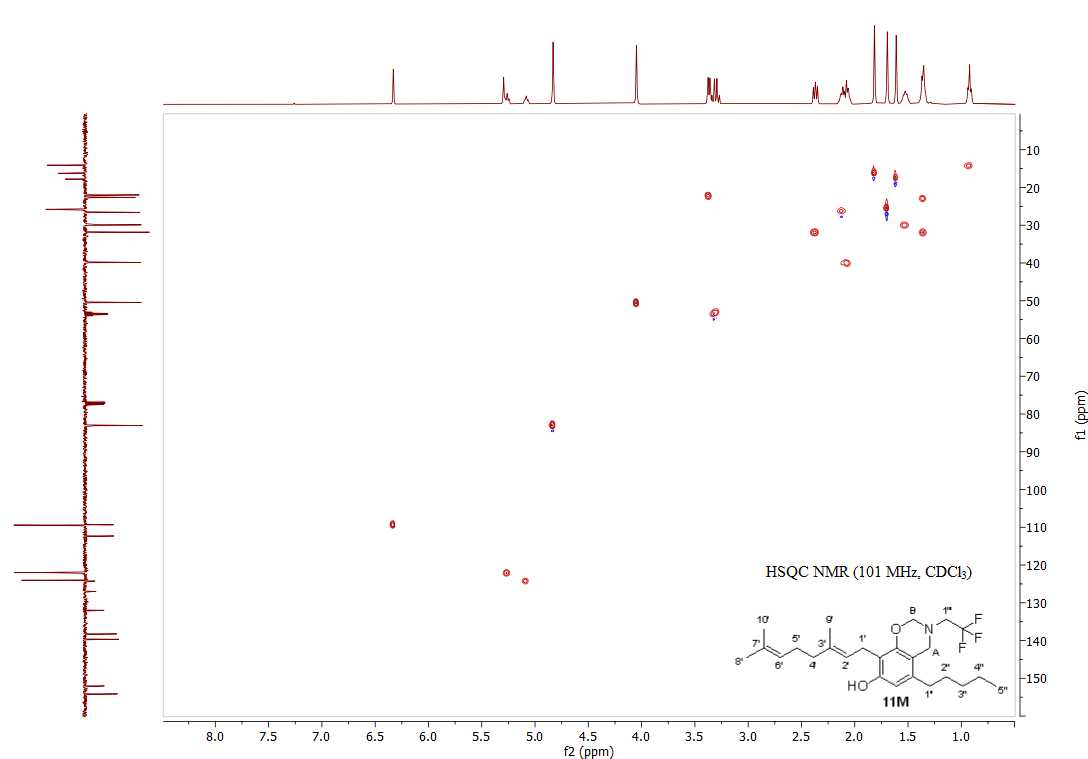


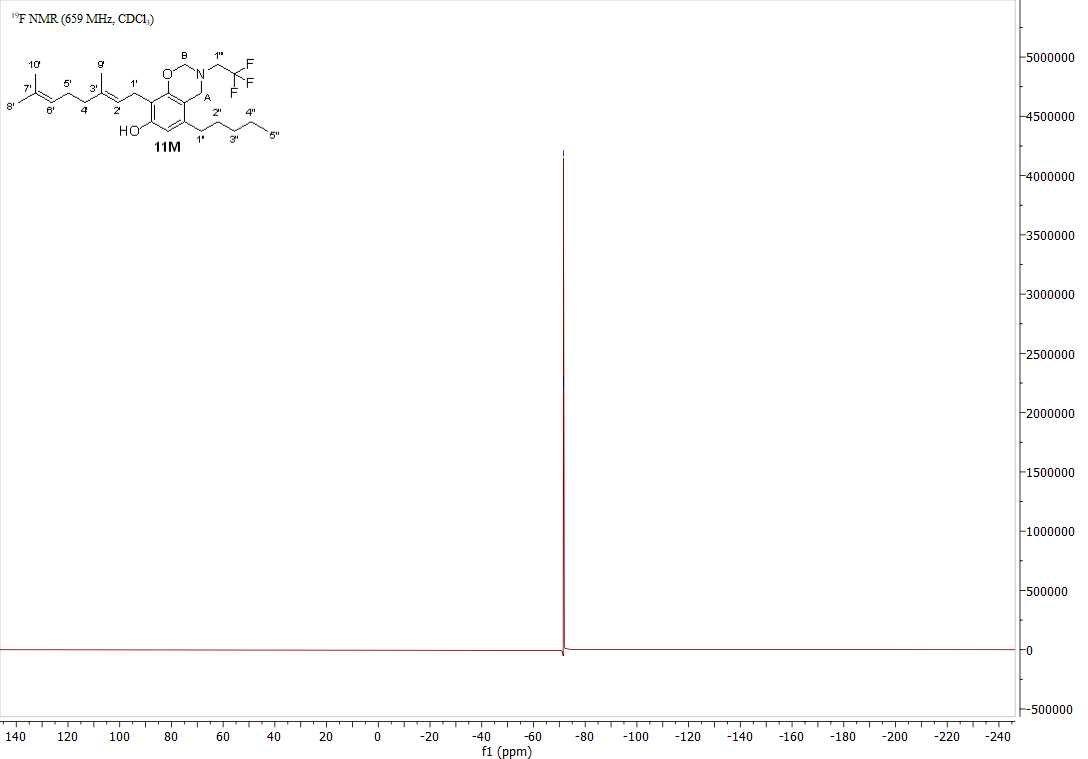


**NMR spectra of compound 12D**


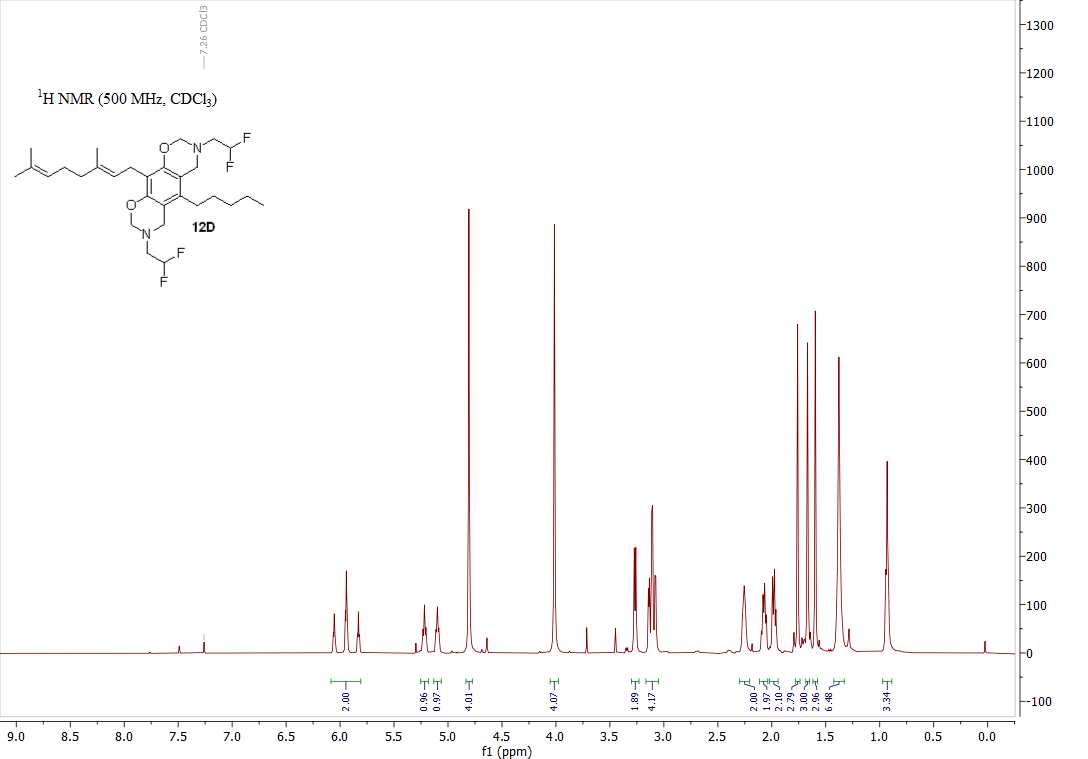


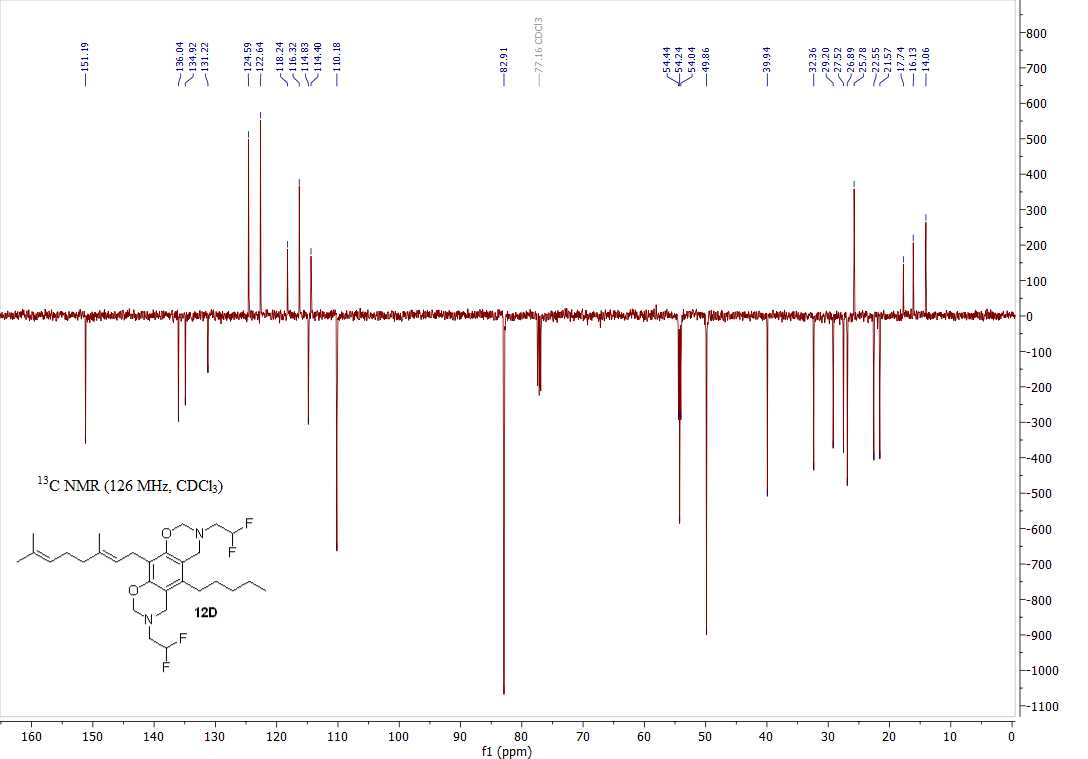


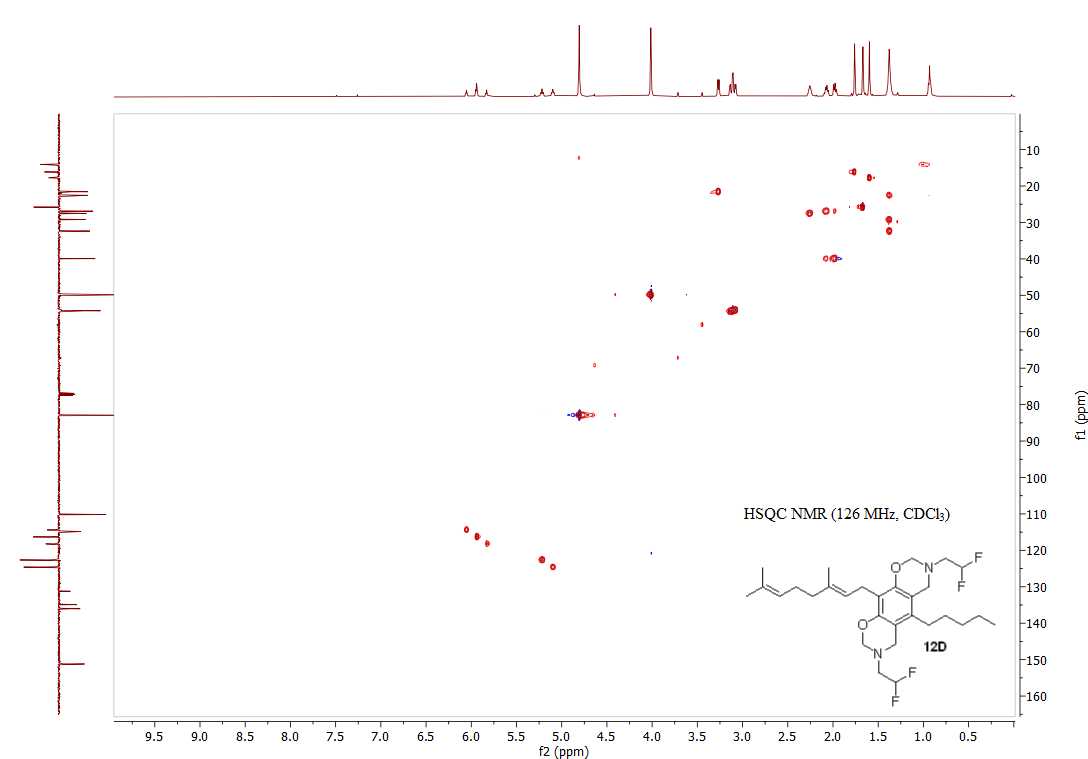


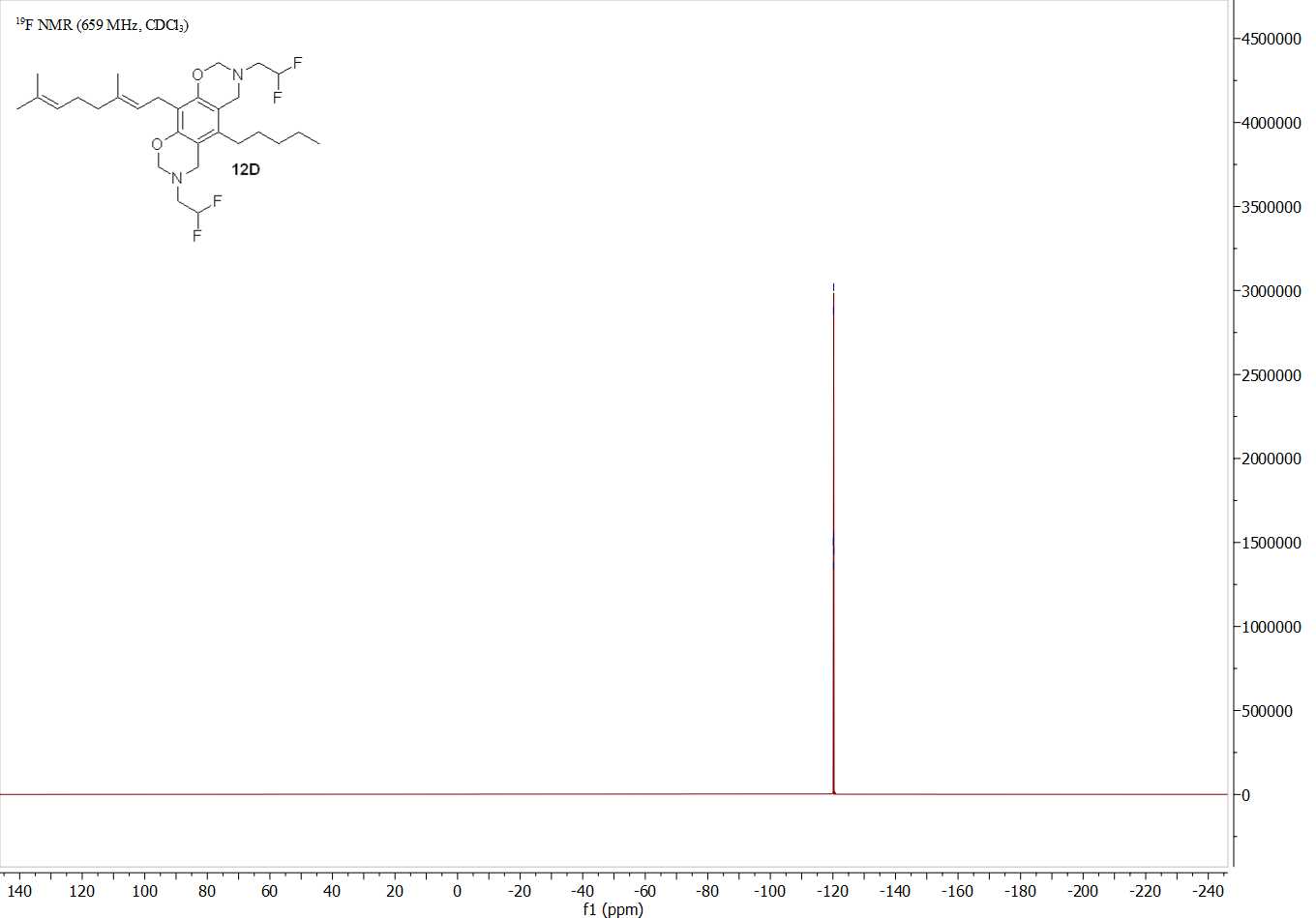


**NMR spectra of compound 12M**


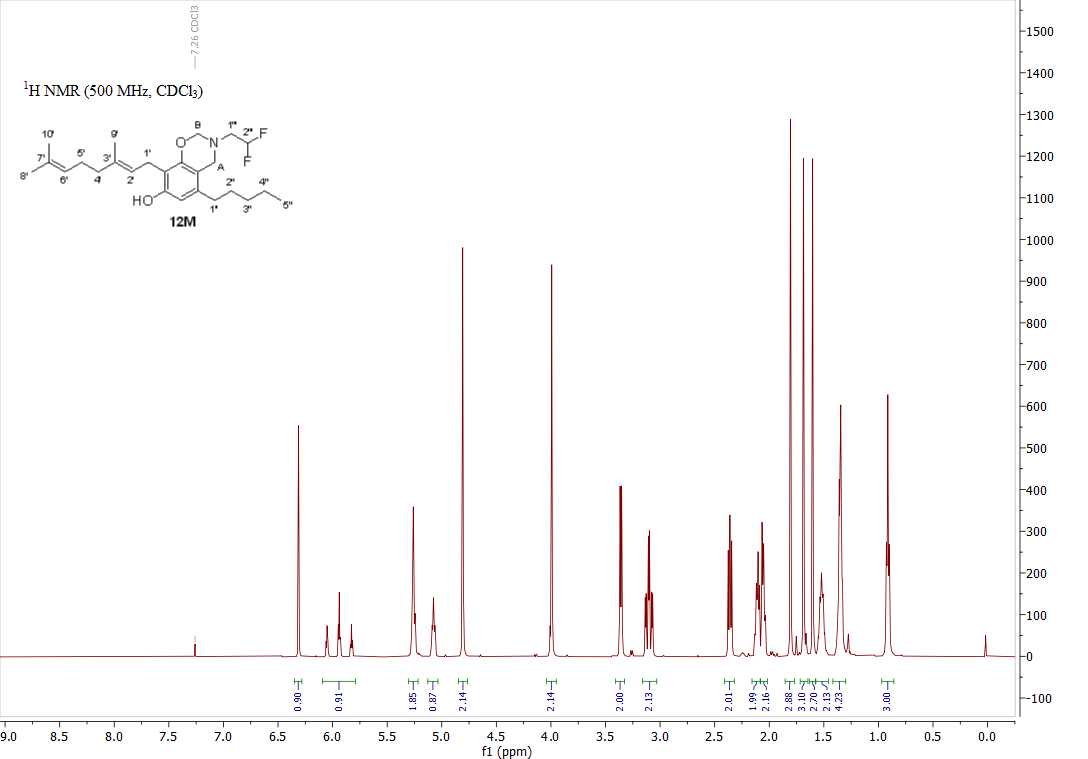


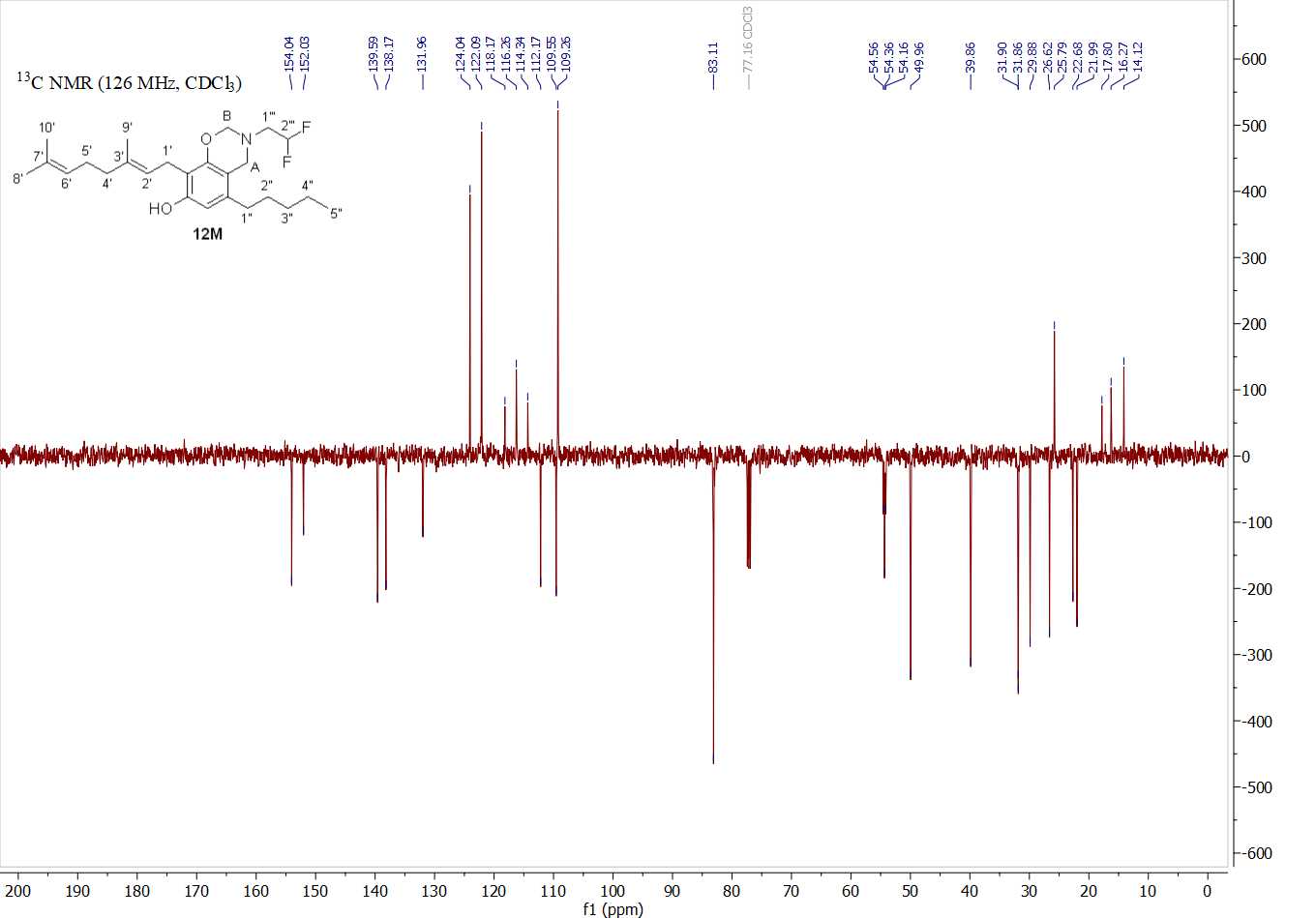


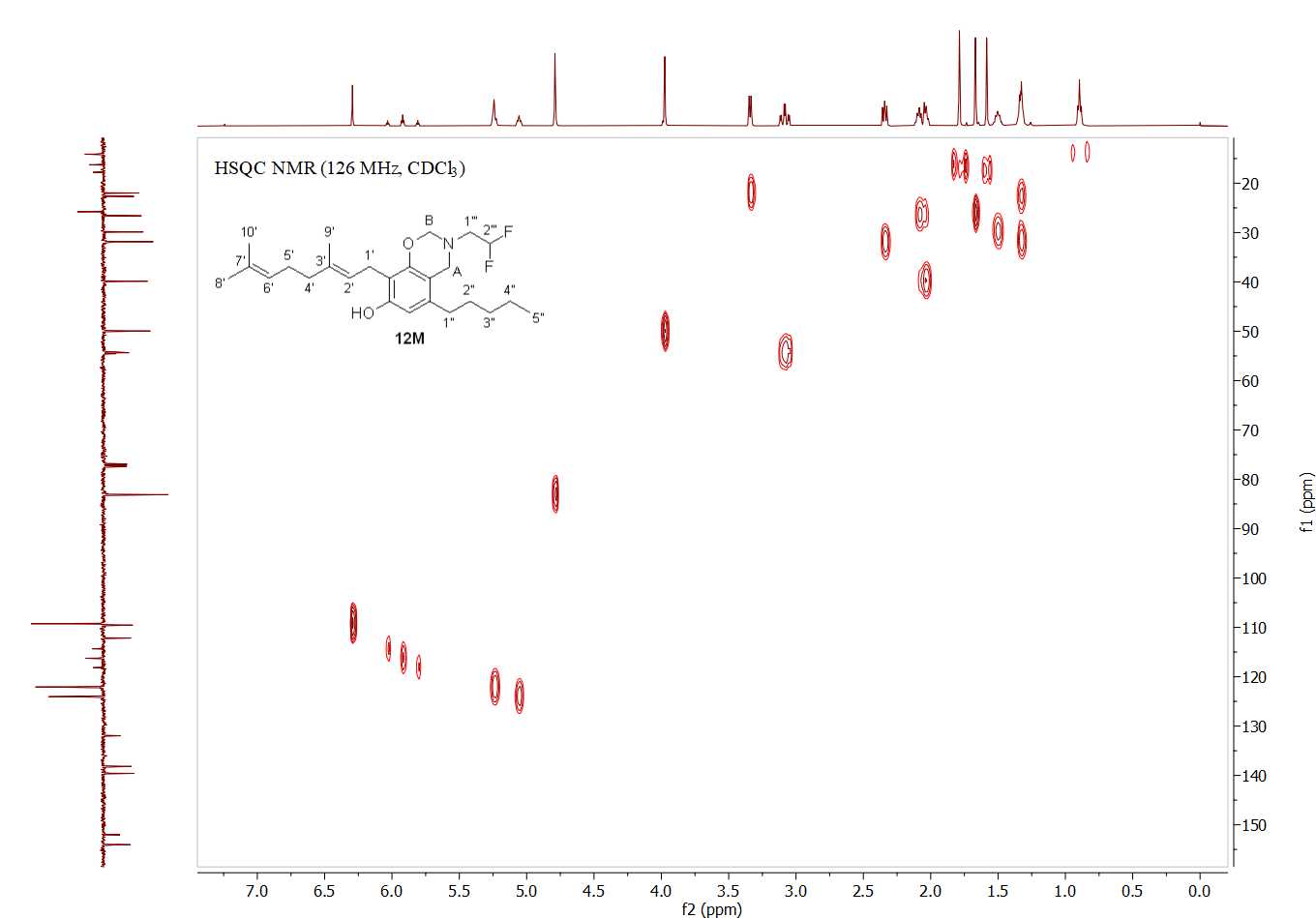


**
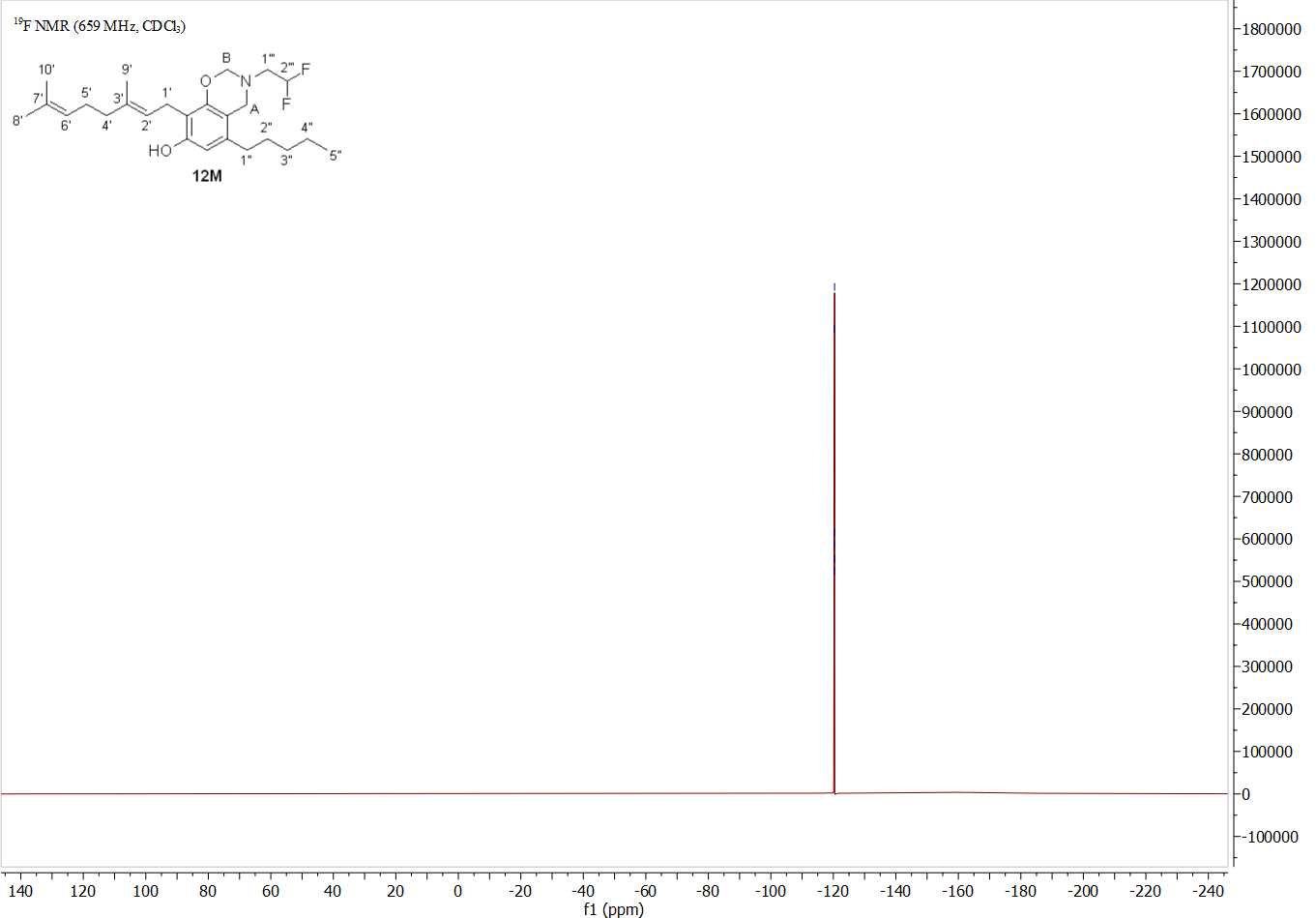
**

**NMR spectra of compound 13D**


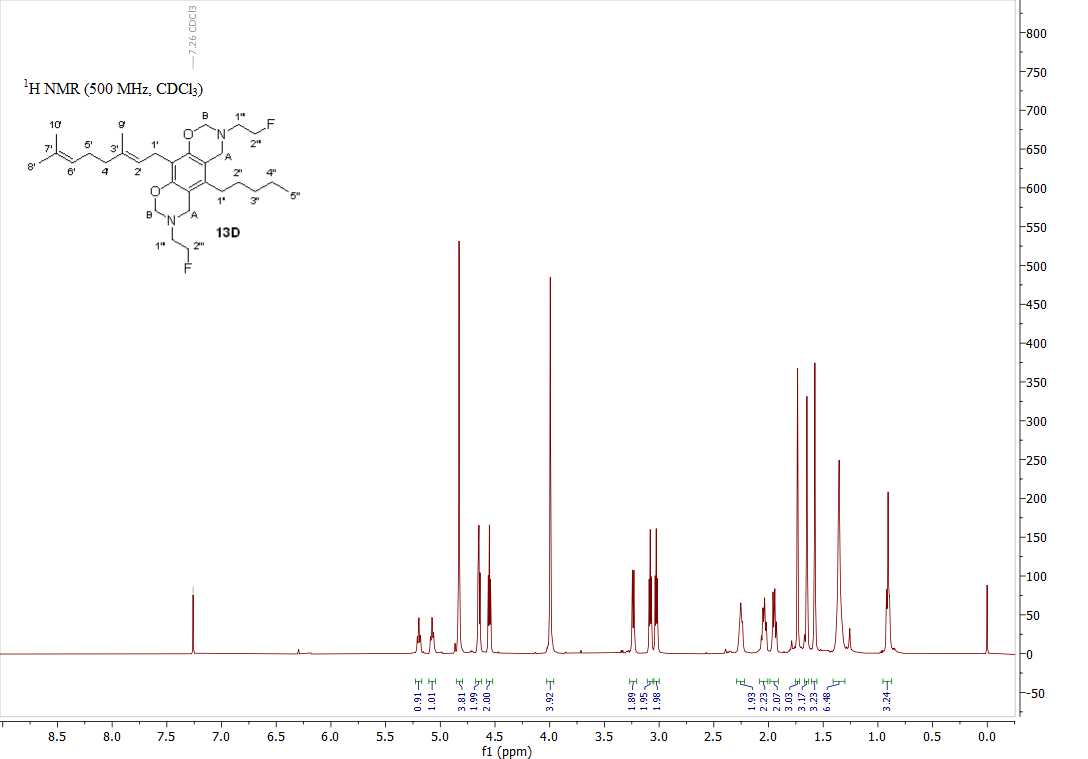


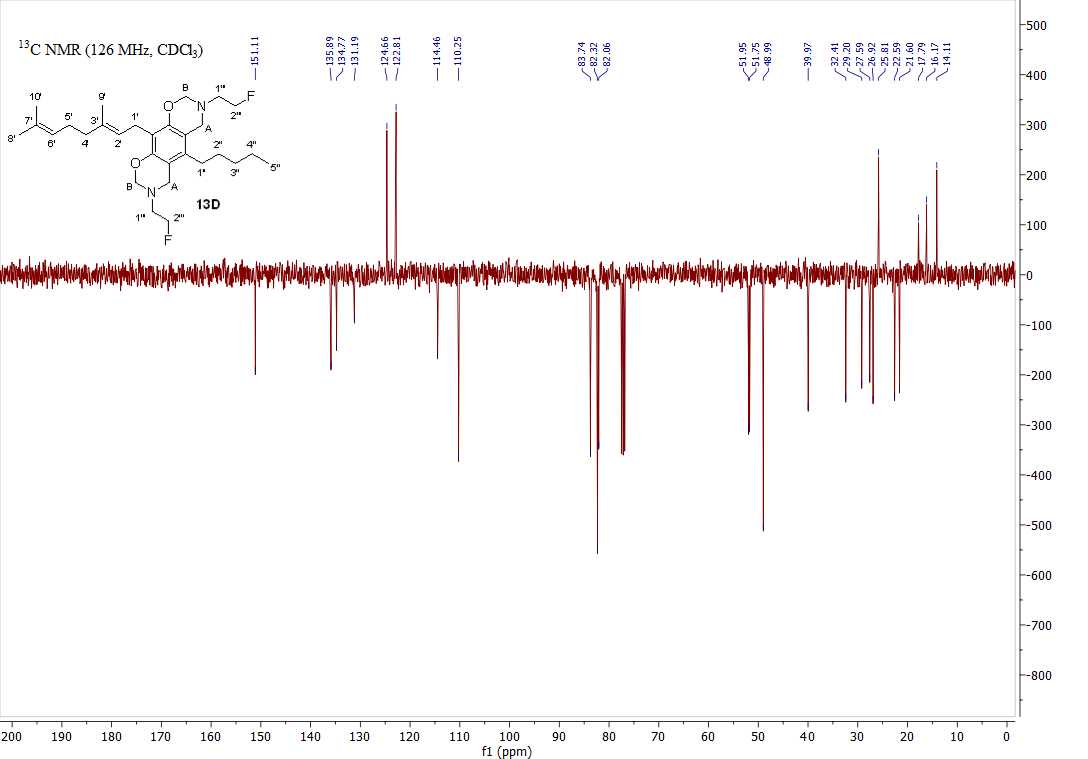


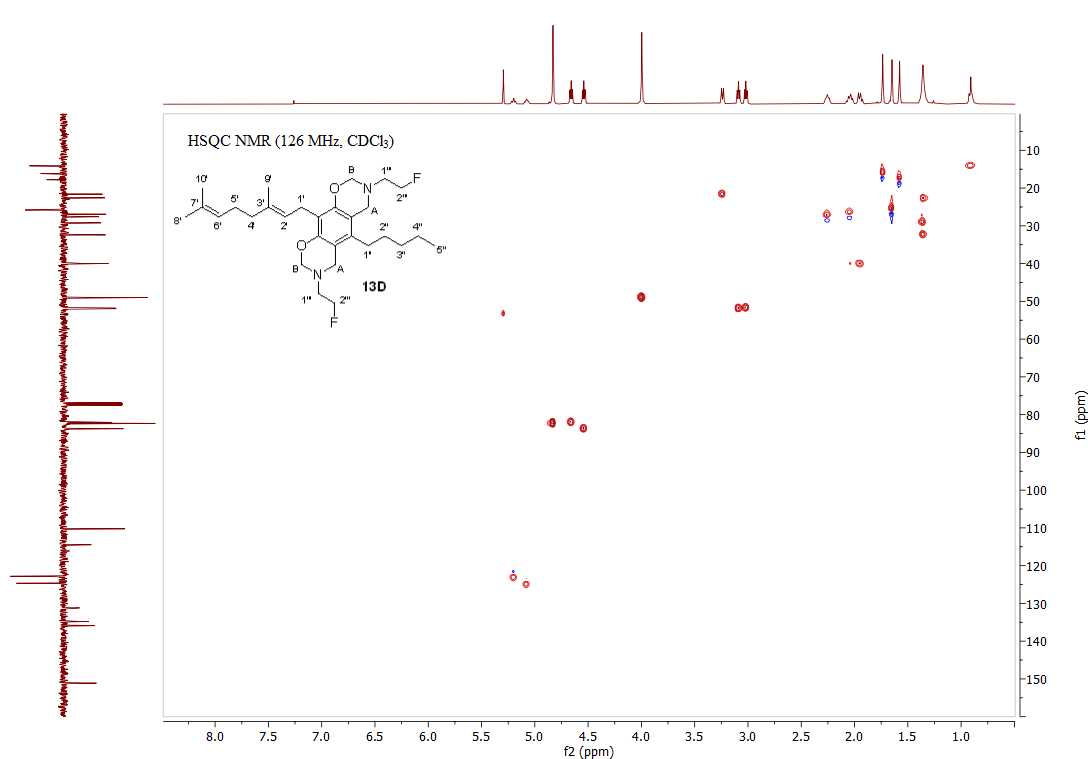


**
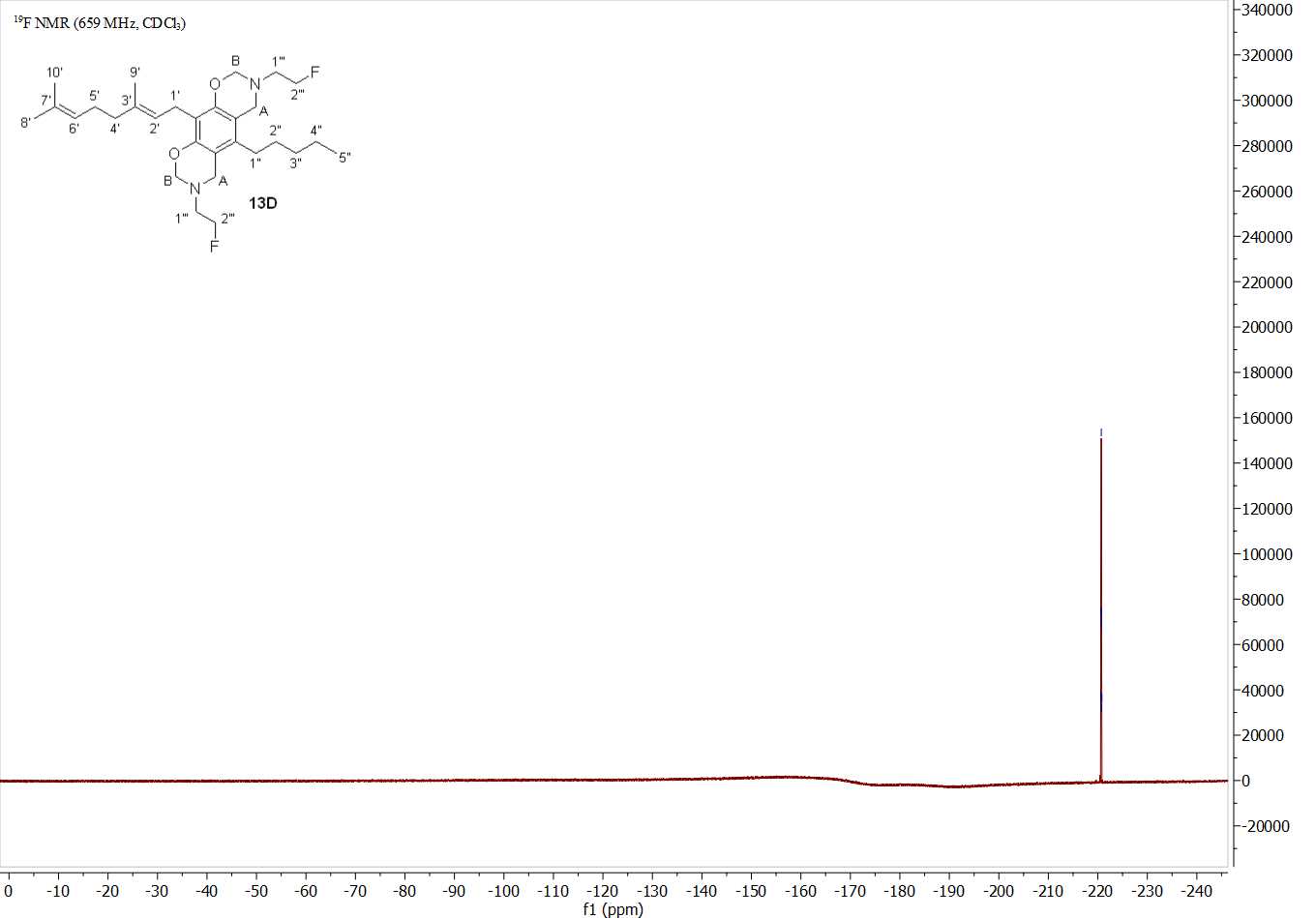
**

**NMR spectra of compound 13M**


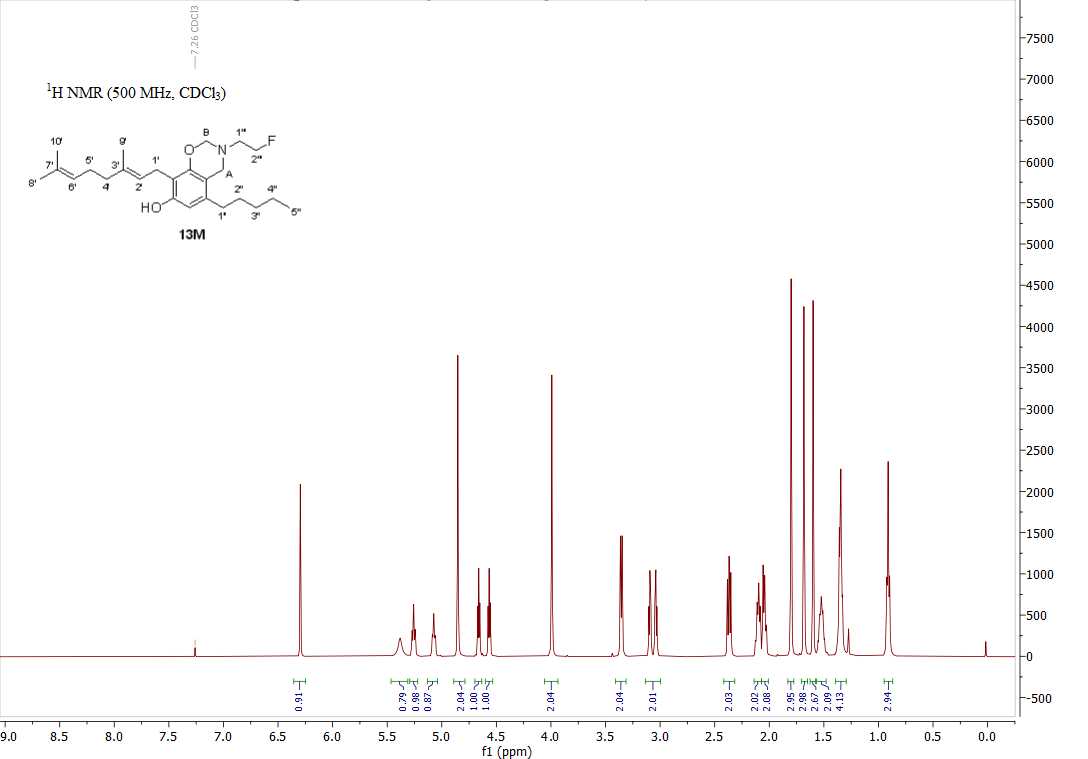


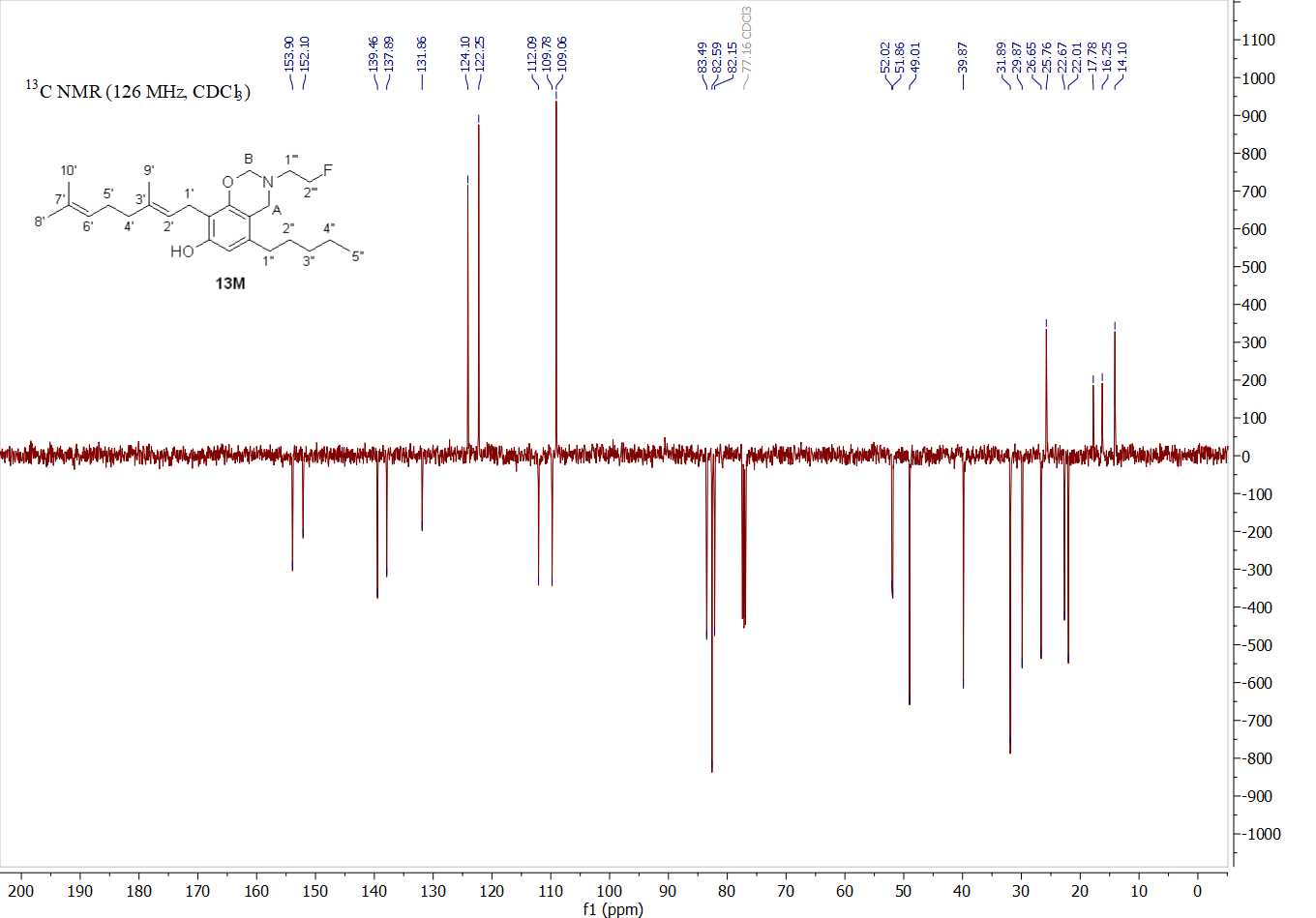


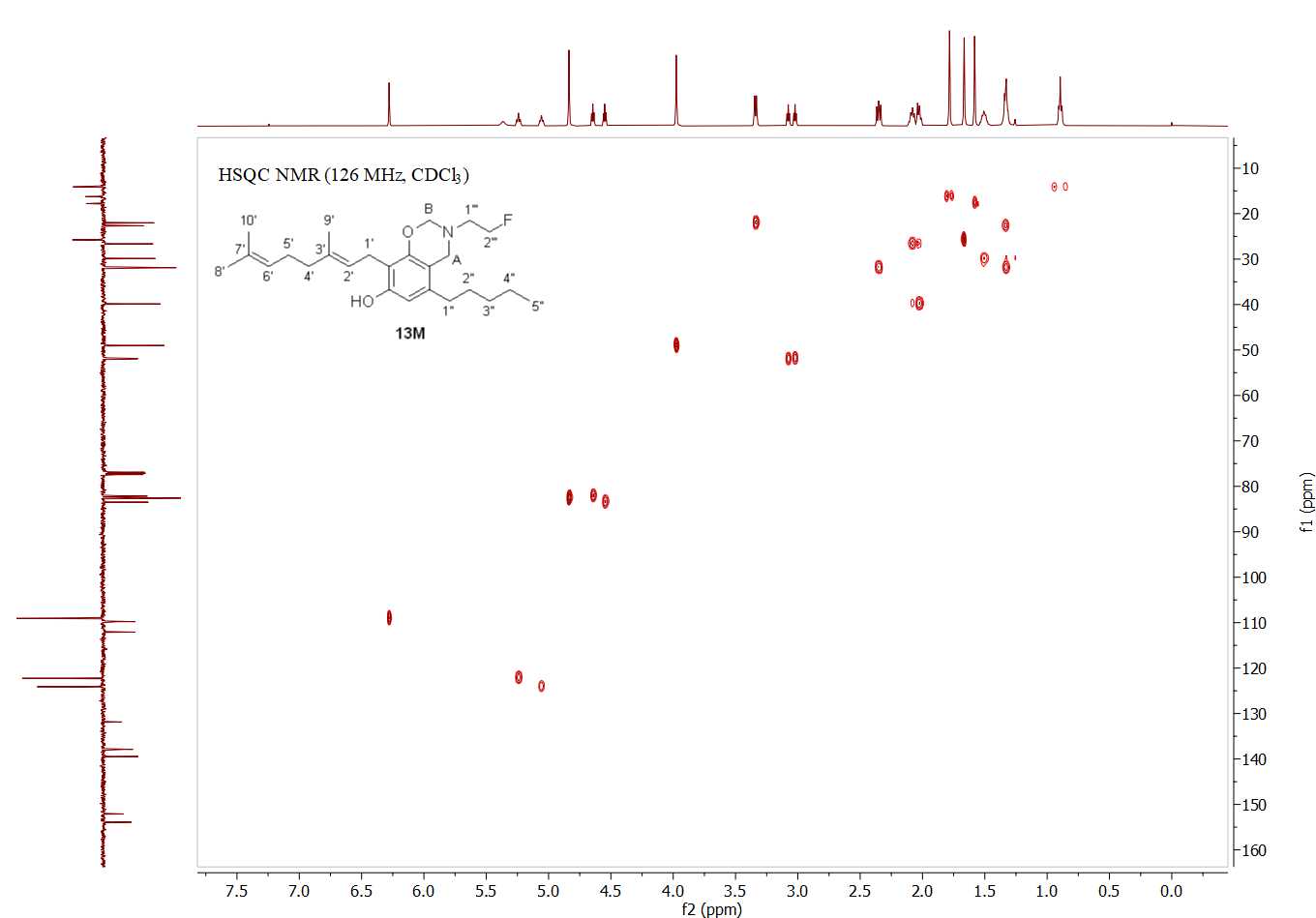


**
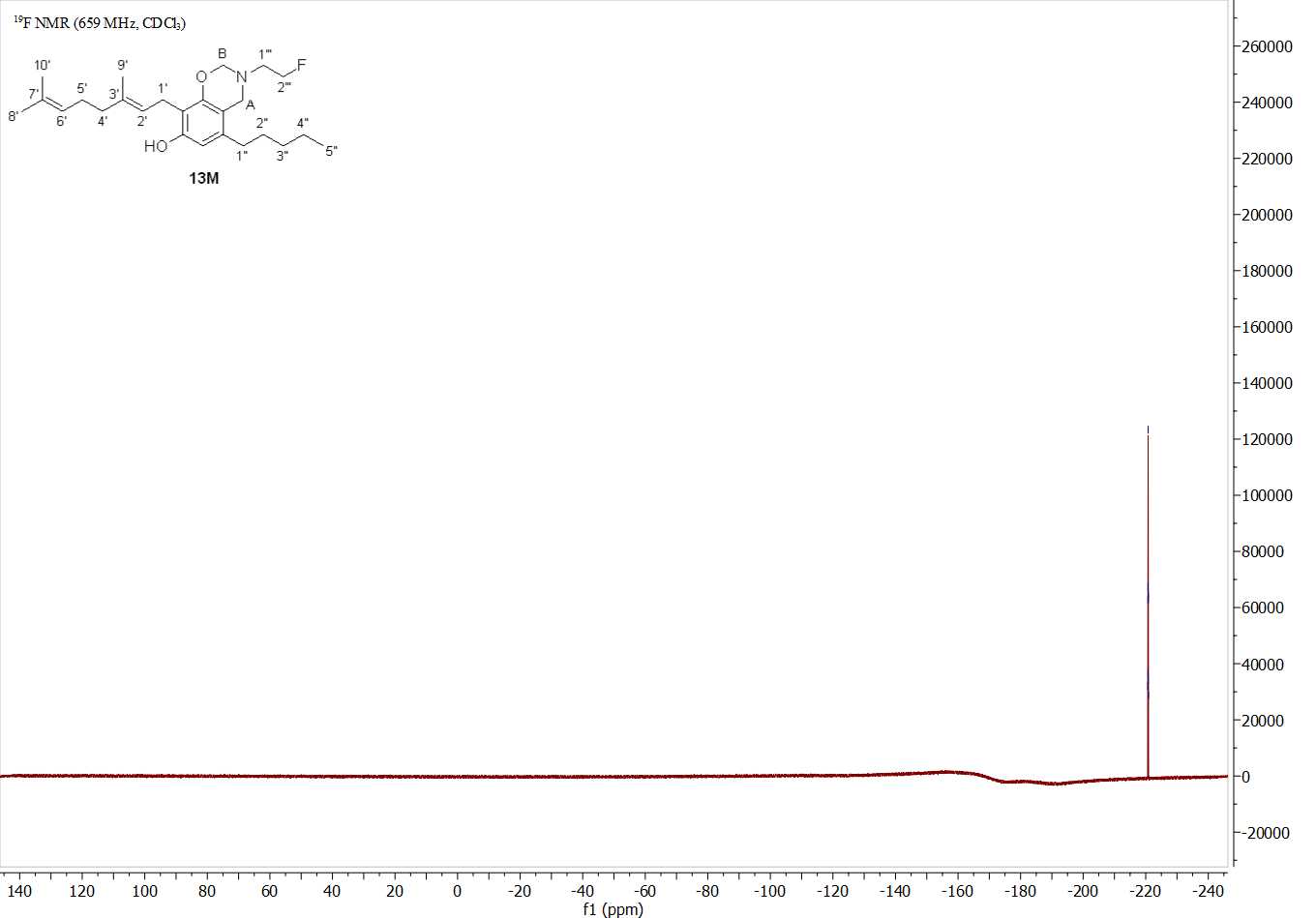
**

**NMR spectra of compound 14M**


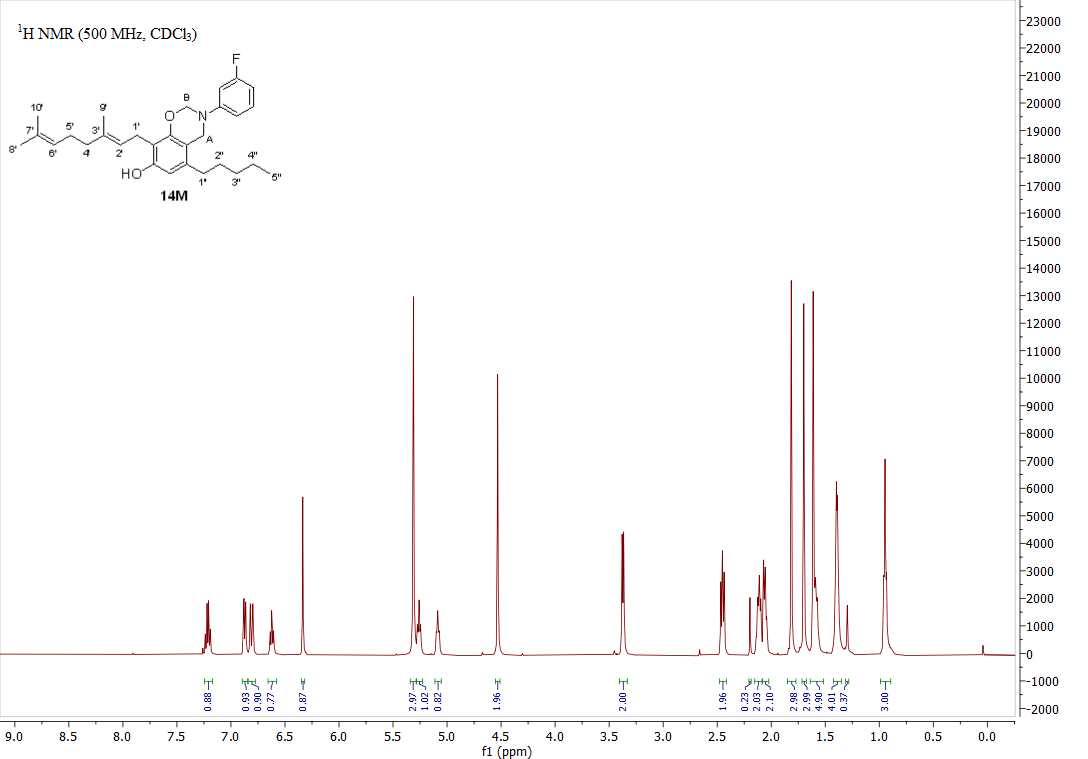


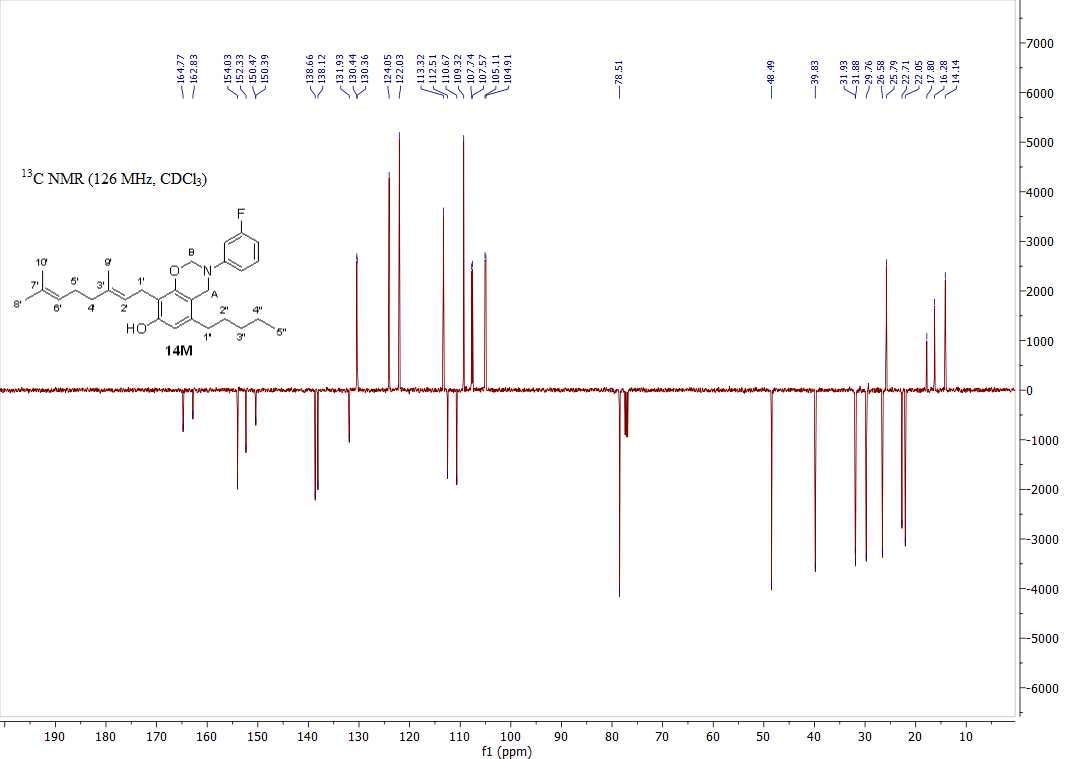


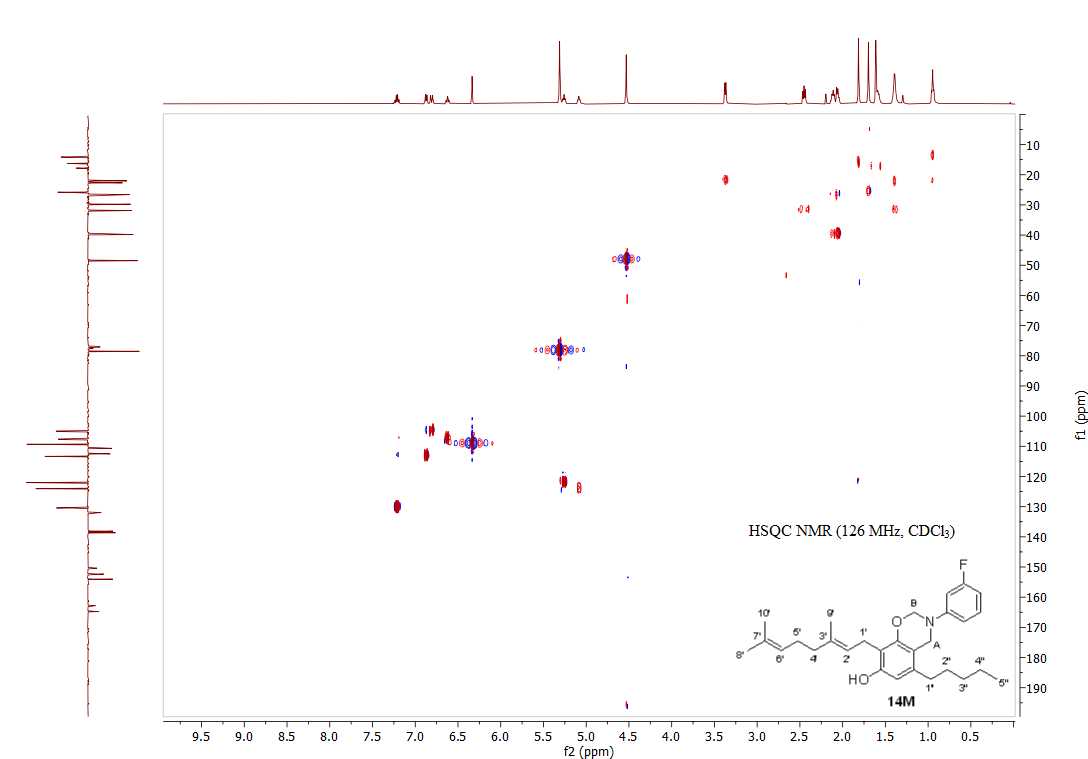


**
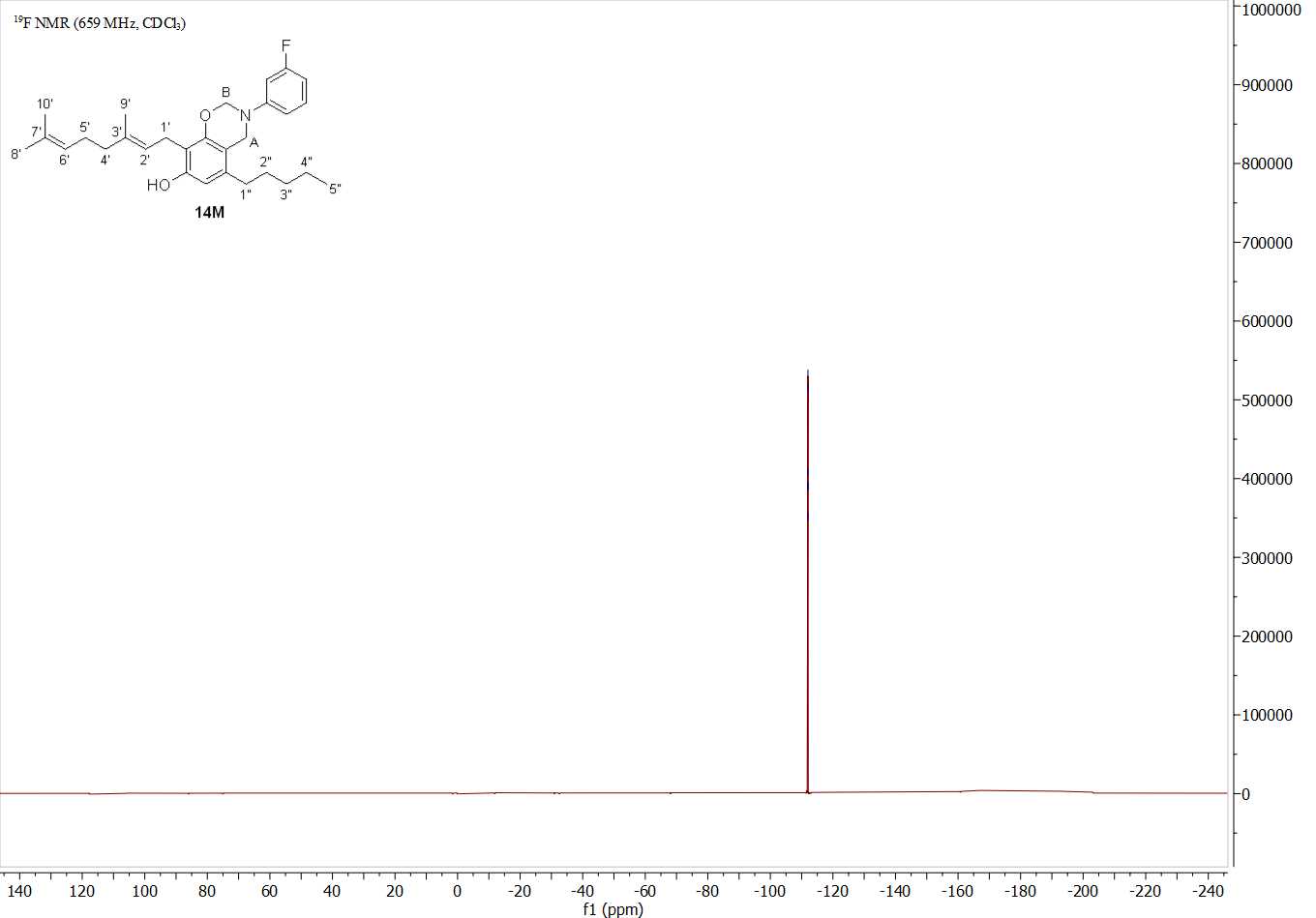
**

**NMR spectra of compound 15D**


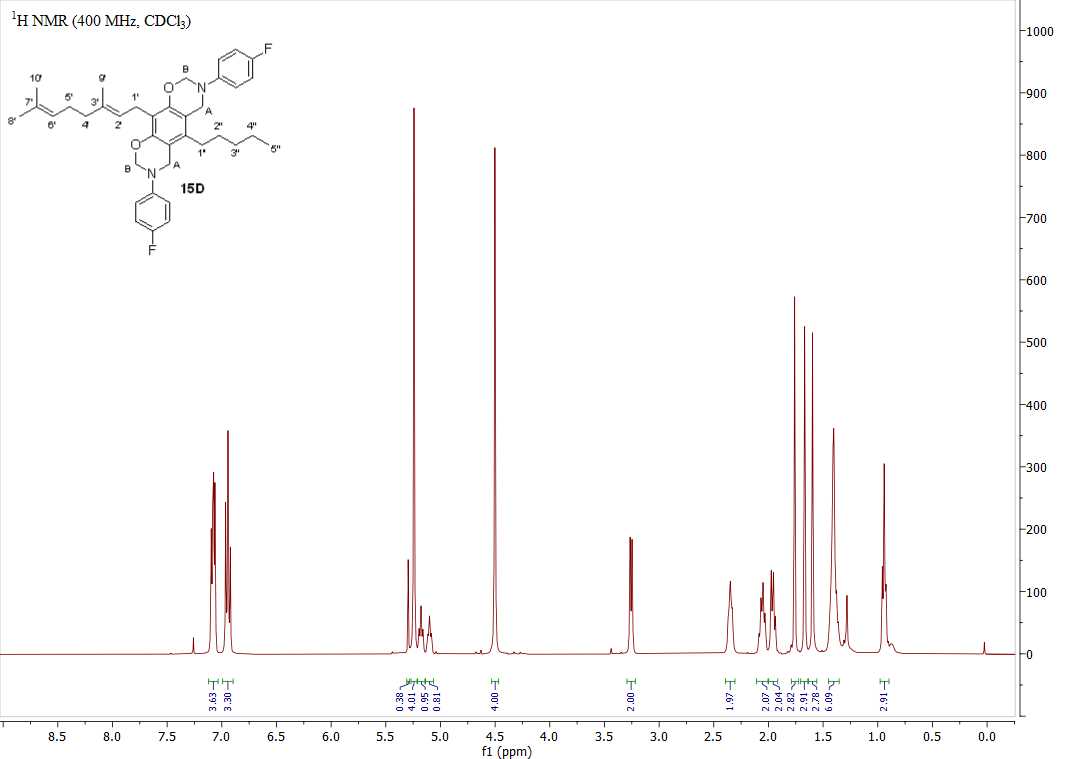


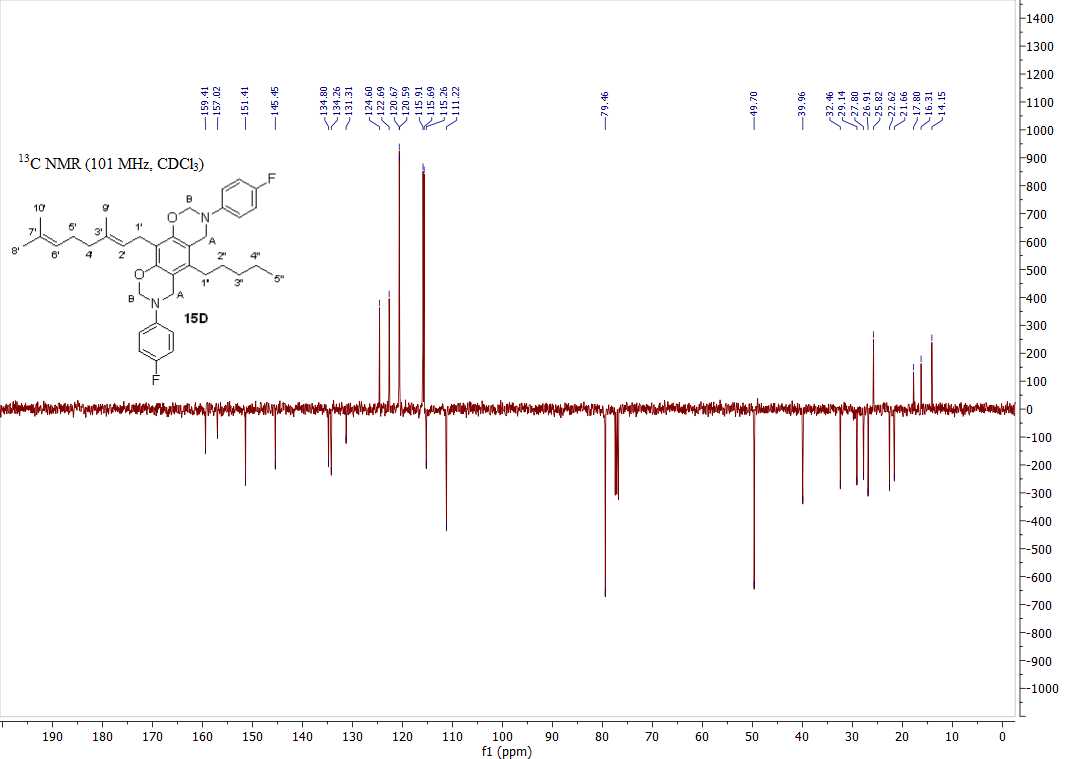


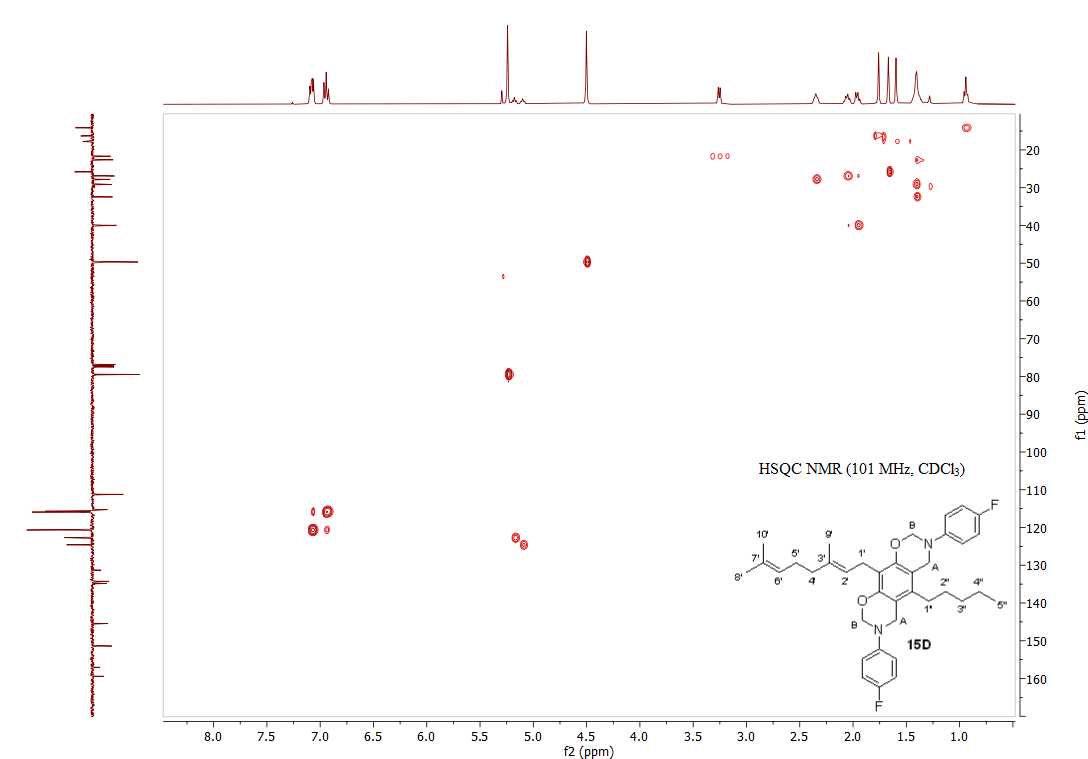


**
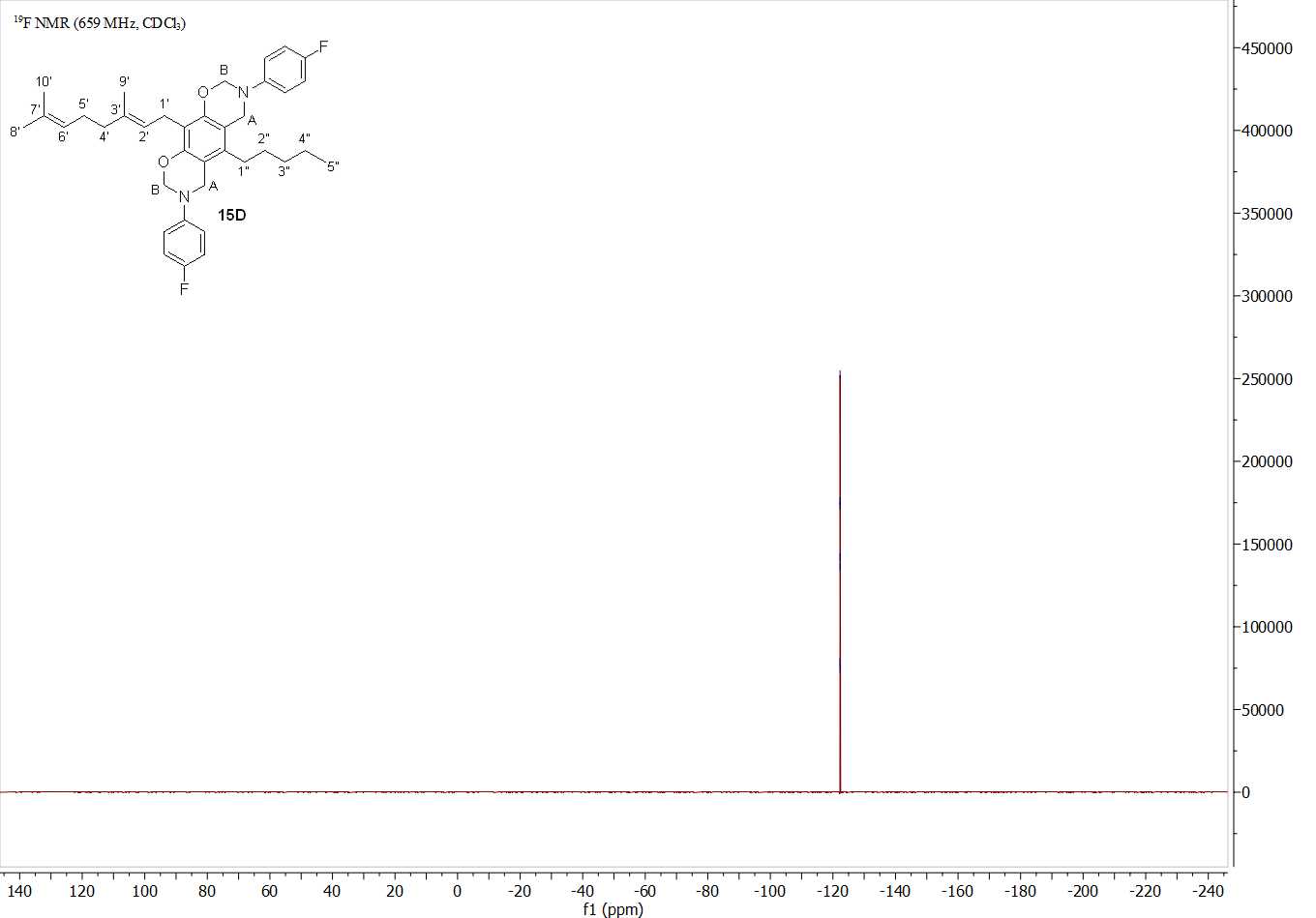
**

**NMR spectra of compound 15M**


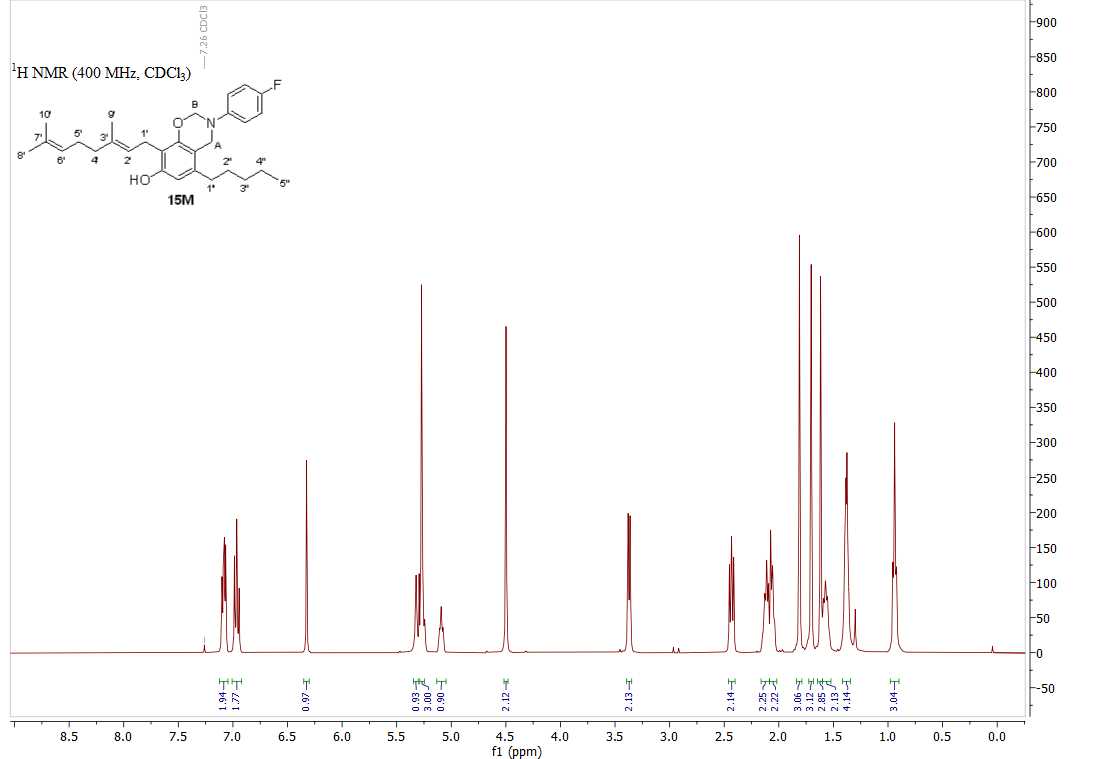


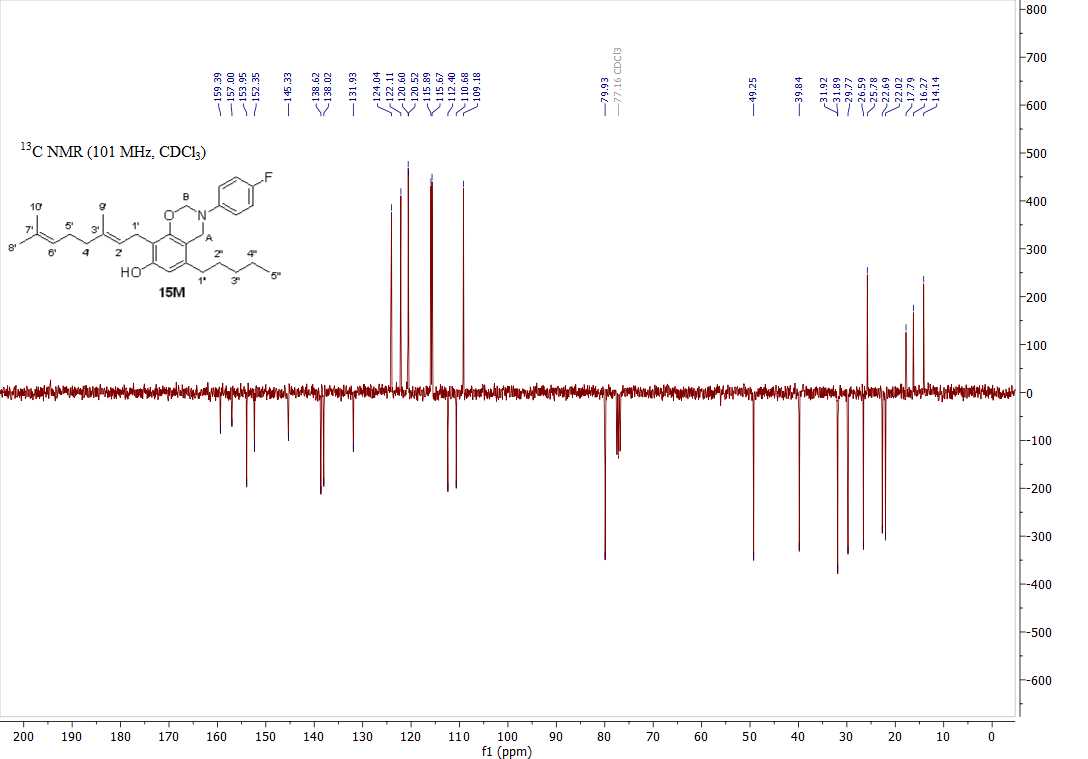


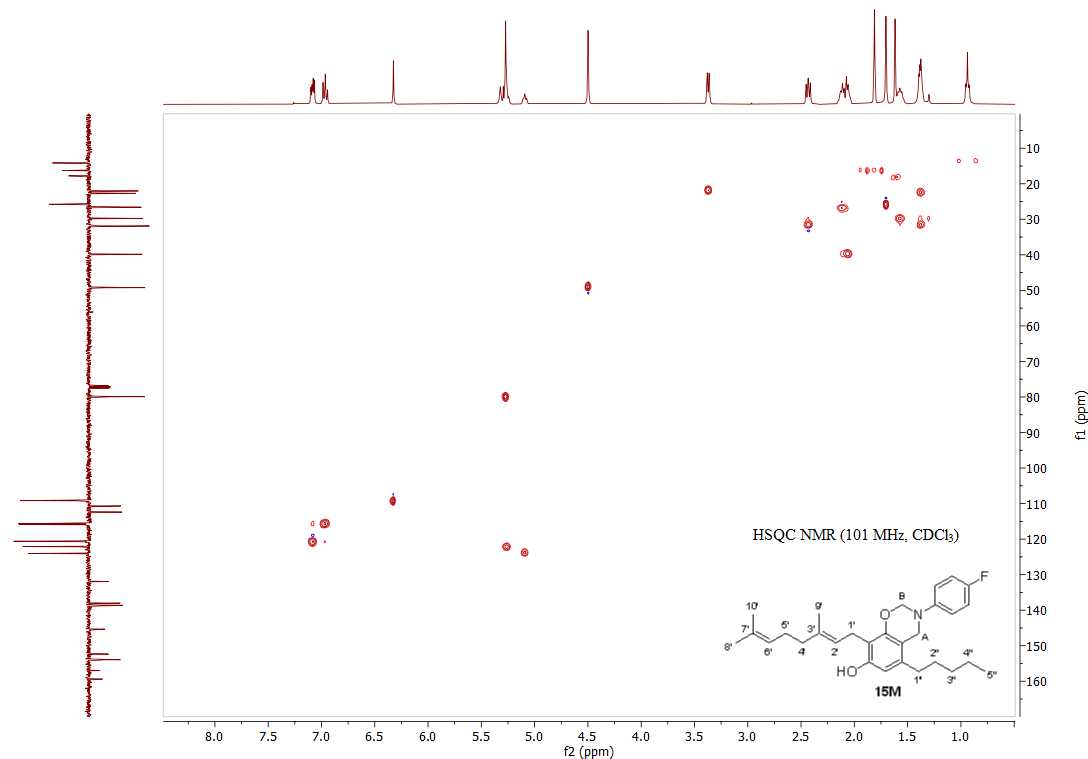


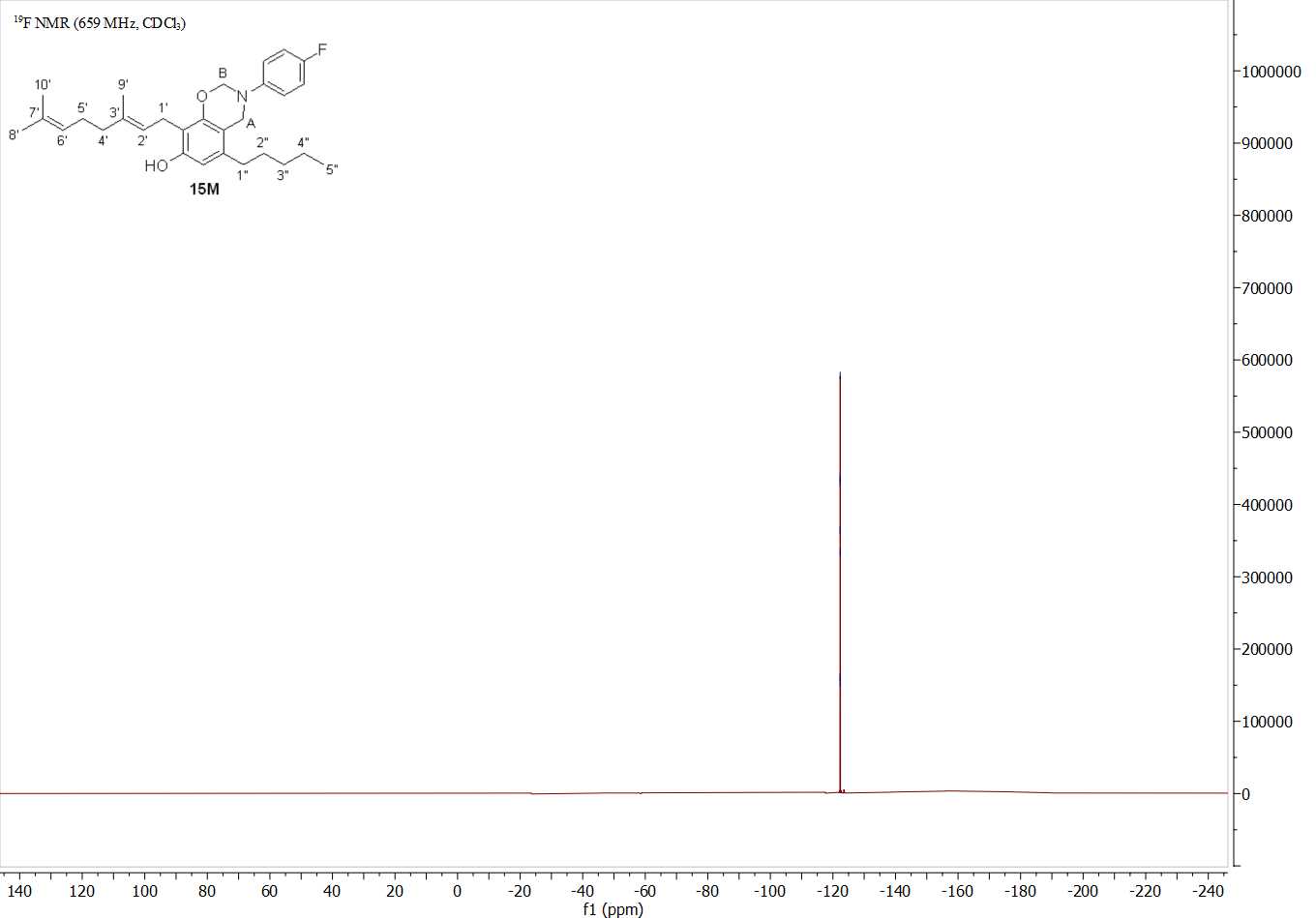

Supplement: Supplementary file 1 — Supplementary Material 1. [file 42238_2026_403_MOESM1_ESM.docx]
